# Azasugar inhibitors as pharmacological chaperones for Krabbe disease†
†Electronic supplementary information (ESI) available: Details of IGF, AGF, IGL and DIL syntheses including NMR spectra. Table containing X-ray data collection and refinement statistics. Dose dependence of thermal stabilization of GALC. Controls for DSF assays. IGF- and AGF-mediated stabilization of GALC is buffer and pH dependent. See DOI: 10.1039/c5sc00754b

‡
‡Data deposition: the atomic coordinates and structure factors have been deposited in the Protein Data Bank, http://www.pdb.org [PDB ID codes 4UFH (IGF), 4UFI (AGF), 4UFJ (IGL), 4UFK (DIL), 4UFL (DGN) and 4UFM (DGJ)].


**DOI:** 10.1039/c5sc00754b

**Published:** 2015-03-23

**Authors:** Chris H. Hill, Agnete H. Viuff, Samantha J. Spratley, Stéphane Salamone, Stig H. Christensen, Randy J. Read, Nigel W. Moriarty, Henrik H. Jensen, Janet E. Deane

**Affiliations:** a Department of Haematology , Cambridge Institute for Medical Research , University of Cambridge , Cambridge CB2 0XY , UK . Email: jed55@cam.ac.uk; b Department of Chemistry , Aarhus University , Langelandsgade 140, 8000 Aarhus C. , Denmark . Email: hhj@chem.au.dk; c Physical Biosciences Division , Lawrence Berkeley National Laboratory , Berkeley , CA 94720 , USA

## Abstract

Krabbe disease is a devastating neurodegenerative disorder characterized by rapid demyelination of nerve fibers. This disease is caused by defects in the lysosomal enzyme β-galactocerebrosidase (GALC), which hydrolyzes the terminal galactose from glycosphingolipids. These lipids are essential components of eukaryotic cell membranes: substrates of GALC include galactocerebroside, the primary lipid component of myelin, and psychosine, a cytotoxic metabolite. Mutations of GALC that cause misfolding of the protein may be responsive to pharmacological chaperone therapy (PCT), whereby small molecules are used to stabilize these mutant proteins, thus correcting trafficking defects and increasing residual catabolic activity in cells. Here we describe a new approach for the synthesis of *galacto*-configured azasugars and the characterization of their interaction with GALC using biophysical, biochemical and crystallographic methods. We identify that the global stabilization of GALC conferred by azasugar derivatives, measured by fluorescence-based thermal shift assays, is directly related to their binding affinity, measured by enzyme inhibition. X-ray crystal structures of these molecules bound in the GALC active site reveal which residues participate in stabilizing interactions, show how potency is achieved and illustrate the penalties of aza/iminosugar ring distortion. The structure–activity relationships described here identify the key physical properties required of pharmacological chaperones for Krabbe disease and highlight the potential of azasugars as stabilizing agents for future enzyme replacement therapies. This work lays the foundation for new drug-based treatments of Krabbe disease.

## Abstract 2

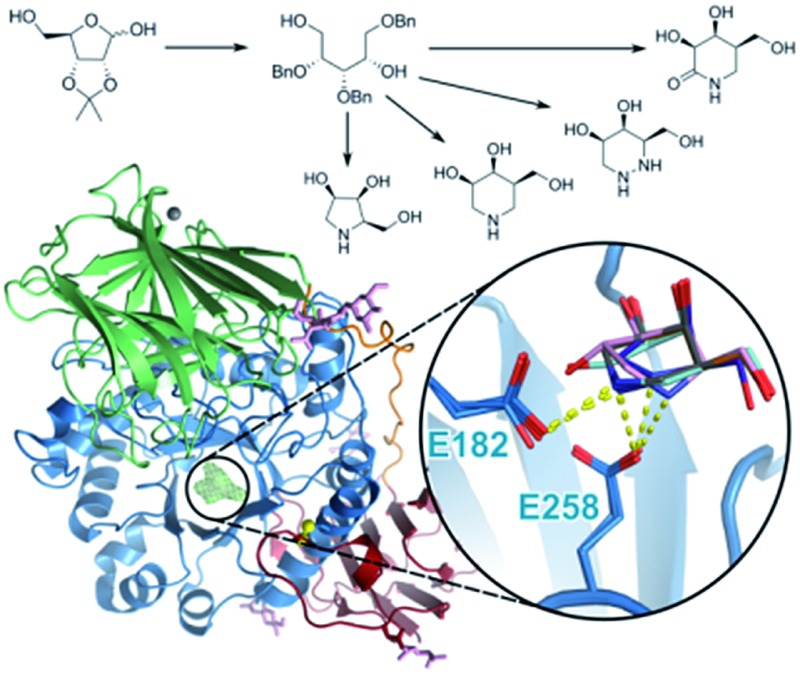
Modified azasugar molecules have been synthesized and characterized as excellent pharmacological chaperone candidates to treat the neurodegenerative disorder Krabbe disease.

## Introduction

The lysosomal enzyme β-galactocerebrosidase (GALC, also known as β-galactosylceramidase; glycosyl hydrolase family 59) is essential for the normal catabolism and recycling of galactosphingolipids. GALC catalyzes the removal of the terminal galactose moiety from substrates including galactosylceramide, the principal lipid component of myelin, and psychosine, a cytotoxic metabolite. Krabbe disease (also known as globoid cell leukodystrophy) is a rare, inherited autosomal recessive disorder caused by loss of GALC function, leading to devastating and ultimately fatal neurodegeneration.^1^ As well as its role in Krabbe disease pathogenesis, GALC function has been linked to cell division, cancer metabolism, primary open-angle glaucoma, necrotizing enterocolitis and maintenance of a haematopoietic stem cell niche.^2–6^

GALC folds in the endoplasmic reticulum (ER), where it is post-translationally modified by N-linked glycosylation. Following processing in the trans-Golgi network (TGN), GALC becomes the cargo for the cation-independent mannose-6-phosphate receptor which targets it to lysosomes either directly from the TGN or indirectly *via* secretion and re-uptake.^7–9^ Galactosphingolipid substrates are then hydrolyzed by GALC in the lysosome.

Removal of the galactosyl moieties by GALC occurs *via* a retaining two-step glycosidic bond hydrolysis reaction.^10^ X-ray crystal structures of GALC identified key catalytic residues and conformational changes of active site residues during catalytic cycling.^11^ In the first step, the carboxylate group of E258 acts as a nucleophile on the anomeric position, forming a covalent enzyme-intermediate complex and releasing the first product (ceramide or sphingosine). In the second step, E182, the general acid/base, deprotonates a water molecule, which attacks the anomeric carbon atom releasing the second product (galactose) with overall retention of anomeric configuration. GALC has been established to have optimal enzyme activity at pH 4.6 consistent with its site of action in the acidic lysosomal environment.^11^

More than 110 mutations have been identified that effect GALC mRNA processing or cause deletions, frameshifts and missense mutations.^12–20^ The most common mutation is a 30 kb deletion resulting in complete absence of functional protein.^16,21^ Other forms of Krabbe disease are caused by protein truncation, catalytic inactivity, misfolding, mistargeting and premature degradation.^22^ Loss of GALC function causes accumulation of the cytotoxic metabolite psychosine, leading to cell death and widespread demyelination throughout the central and peripheral nervous systems. There is no cure for Krabbe disease and most infants die before reaching two years of age. Currently the best available treatment is hematopoietic stem-cell transplantation, which is effective in pre-symptomatic individuals but carries a significant mortality risk.^23^ Peripheral symptoms of related lysosomal storage diseases can be relieved by enzyme replacement therapy, which involves regular infusions of recombinant enzyme. In Krabbe disease the critical pathology occurs in the central nervous system meaning this therapy is currently considered unsuitable due to the inability of administered enzyme to cross the blood-brain barrier.^24–26^

Pharmacological chaperone therapy (PCT) has recently emerged as an alternative strategy for treating diseases caused by partially defective proteins. In cases where mutant enzyme is trapped in the ER due to instability or misfolding, specific binding of a small molecule chaperone is hypothesized to stabilize the correctly-folded enzyme, allowing functional material to leave the ER, and decreasing removal of the protein by ER-associated degradation. Although not completely understood, several biochemical mechanisms for pharmacological chaperones (PCs) have been proposed including the acceleration of folding, slowing of unfolding, template-based induction of correct folding, and thermodynamic stabilization.^27^ To attain selectivity, PCs are often active-site-specific competitive inhibitors; hence the ideal PC would bind the enzyme in the ER, stabilize the protein, restore correct trafficking, then dissociate in lysosomes where the PC would be outcompeted by an excess of substrate. Restoration of just 10–15% of activity is sufficient to prevent disease.^28,29^

A recent attempt to identify new PCs for Krabbe disease measured the effects of 1280 compounds on GALC activity in patient fibroblasts.^30^ This approach was unable to identify any molecules with statistical significance, and was confounded by toxicity of some compounds. Another study reported impaired trafficking and reduced enzymatic activity in three missense GALC mutations.^22^ This study identified α-lobeline as a promising PCT candidate for the hyperglycosylation mutant D528N; however this compound was ineffective with other tested Krabbe disease mutations. Recent docking experiments with α-lobeline have predicted multiple binding sites, consistent with a non-specific chemical chaperone.^31^

For several related diseases, the development of PCT candidate molecules has been greatly accelerated by structural and mechanistic investigations of the glycosyl hydrolase enzymes involved.^32–34^ A comprehensive understanding of active site architecture has enabled the identification of carbohydrate mimetic compounds that bind and stabilize partially defective enzymes. Iminosugars and azasugars represent a particularly promising class of PCT candidate molecules with high solubility, excellent biodistribution and low toxicity:^35–37^ 1-deoxy-*galacto*-nojirimycin (DGJ) stabilizes α-galactosidase A and phase III clinical trials are underway to evaluate its efficacy in Fabry disease patients.^38,39^ Three related molecules, isofagomine,^40^ *N*-butyl-, and *N*-nonyl-deoxynojirimycin^34^ partially rescue enzyme activity in cell lines expressing acid β-glucocerebrosidase variants causing Gaucher disease.^41–43^

**Fig. 1:**
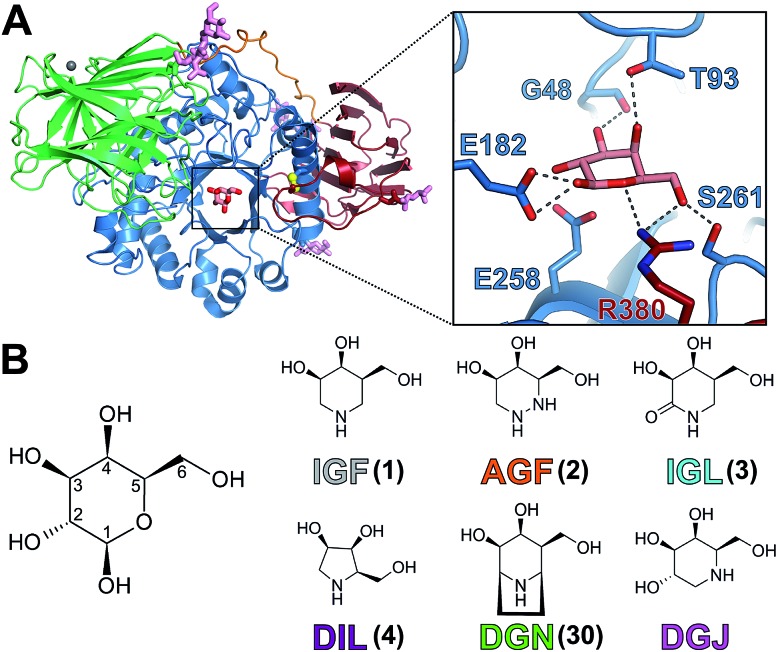
Structure of GALC and azasugar compounds. (A) Ribbon diagram of GALC illustrating the β-sandwich (red), TIM-barrel (blue) and lectin (green) domains with β-d-galactose in the active site and N-linked glycans (pink sticks). Hydrogen bonding interactions (dashed lines) between galactose and active site residues are shown (zoomed box). (B) Pyranose ring numbering of β-d-galactose illustrating the anomeric position and chemical diagrams of the molecules used in this study: iso-*galacto*-fagomine (IGF); aza-*galacto*-fagomine (AGF); iso-*galacto*-fagomine lactam (IGL); dideoxyiminolyxitol (DIL); deoxy-*galacto*-noeurostegine (DGN) and deoxy-*galacto*-nojirimycin (DGJ). Compounds are color coded as indicated throughout this study.

Our recent structures of GALC revealed the mode of binding of substrate, intermediate and product to the enzyme active site, accompanied by conformational changes of active site residues.^11,44^ An extensive and dynamic hydrogen bonding network orients the galactosyl group in the substrate binding pocket during the catalytic cycle (Fig. 1A). These insights provided us with an atomic framework for the design of small molecules that specifically bind the GALC active site. In parallel, we developed new strategies to synthesize several of these compounds from a common starting material. Here we present the synthesis of a series of azasugar derivatives and their biochemical, biophysical and structural characterization as potential new pharmacological chaperone candidates for Krabbe disease. We have identified significant differences in their ability to inhibit and stabilize GALC and highlight the critical importance of specific active site residues for PC binding.

## Results

### Synthesis of IGF and derivatives

**Fig. 2:**
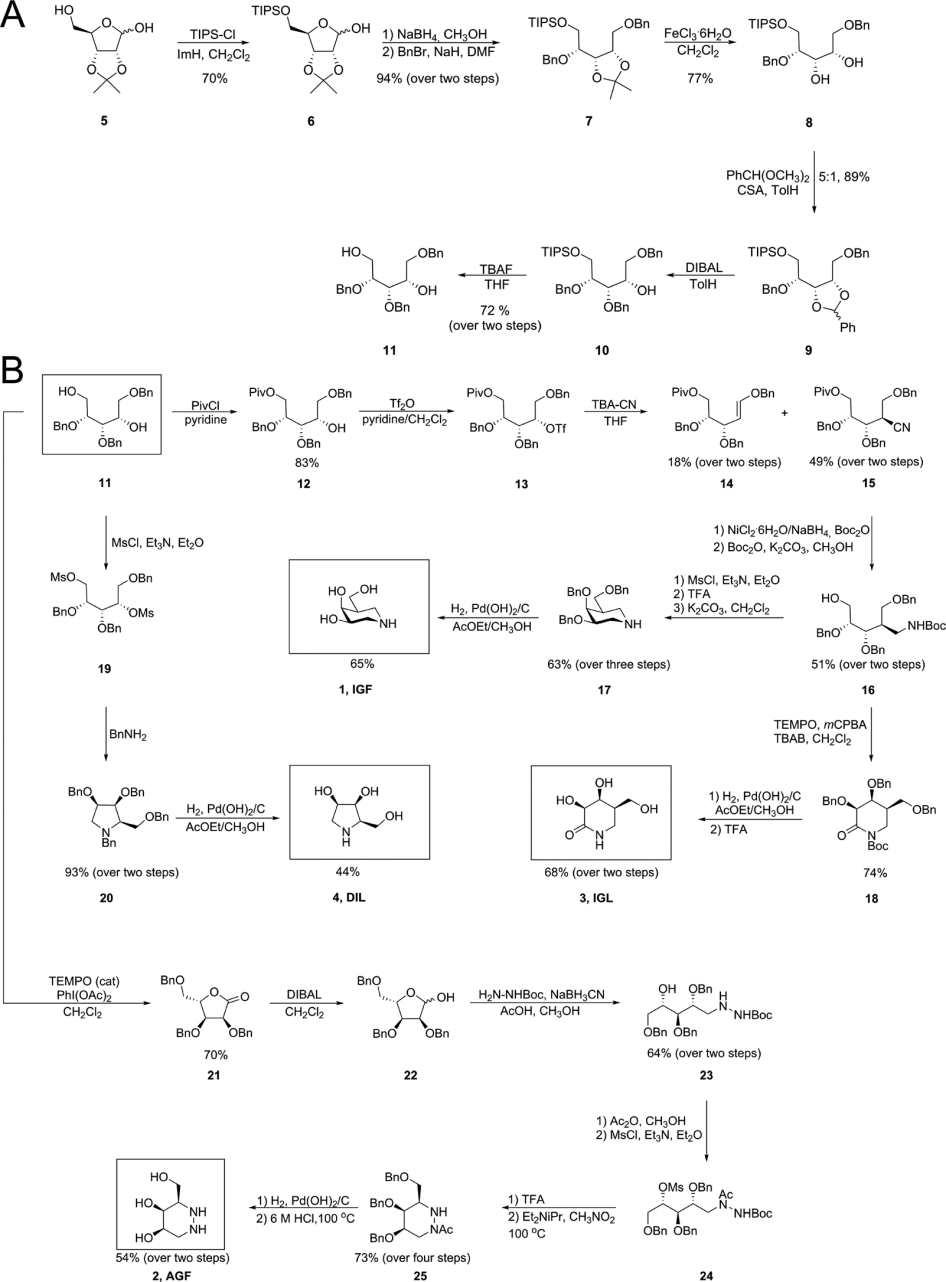
Synthesis of *galacto*-configured azasugars. (A) Synthesis of key intermediate **11** from 2,3-*O*-isopropylidene-d-ribofuranose (**5**). (B) Synthesis of IGF, IGL, DIL and AGF from a common intermediate (**11**).

A recent study looking at potential pharmacological chaperone molecules for GALC showed that derivatives of 1-deoxy-*galacto*-nojirimycin (DGJ, Fig. 1B) carrying a C-linked alkyl chain in the pseudo-anomeric position were weak inhibitors of GALC and only iso-*galacto*-fagomine (IGF) showed significant promise as a PC candidate based on inhibition of GALC.^45^ In general, β-galactosidases are often more potently inhibited by isofagomine-type compounds with nitrogen in place of the anomeric carbon as opposed to the nojirimycin-type counterpart with nitrogen in place of the *endo*-cyclic sugar oxygen.^46^ Our focus was therefore to synthesize and investigate a series of N-containing sugar mimetics that are known to potently inhibit β-galactosidases. For this study, in addition to DGJ (Carbosynth), IGF (**1**), aza-*galacto*-fagomine (AGF, **2**), iso-*galacto*-fagomine lactam (IGL, **3**), dideoxy-imino-lyxitol (DIL, **4**) and deoxy-*galacto*-noeurostegine (DGN, **30**) were chosen based on predicted compatibility with the GALC active site (Fig. 1B). Most of these sugar mimetics have previously been prepared from various starting materials, but here we demonstrate a method for preparing four out of five of these molecules from the same starting material. First, 2,3-*O*-isopropylidene-d-ribofuranose (**5**) underwent *O*-5 mono-silylation with TIPS-Cl to give **6** in 70% yield (Fig. 2A). The lactol functionality was then reduced by NaBH_4_ and the resulting diol benzylated with BnBr to give **7** in 94% over two steps. Selective removal of the isopropylidene group without affecting the TIPS-group proved challenging. However, treatment with iron(iii)chloride resulted in 77% of the desired diol **8**. The next step was formation of a benzylidene protecting group, which went uneventfully in 89% under standard transacetalization conditions with benzaldehyde dimethylacetal and camphorsulfonic acid. A reaction sequence was also attempted starting with d-ribose and forming a 2,3-benzylidene acetal. This approach, however, proved inferior to the one described from **5**. The benzylidene could be opened using DIBAL to give **10** with complete regioselectivity. Having a Piv group instead of the TIPS reduced the stereoselectivity of the reaction and gave a yield of 76% as a 1 : 4.8 mixture in favour of **12** using triethyl silane and TiCl_4_ (reaction not shown). Next, the TIPS group of **10** was removed by TBAF to give diol **11**, which was a common intermediate for the preparation of sugar mimetics **1–4**.

For the synthesis of IGF (**1**)^45,47–52^ the primary alcohol of **11** was selectively protected as a pivaloyl ester with PivCl to give **12** in 83% yield (Fig. 2B). Also formed (not shown) and easily separable from **12** was the di-*O*-pivaloylated congener of **12** in 6% yield. The remaining free alcohol of **12** underwent triflation with triflic anhydride and displacement with inversion of stereochemistry with tetrabutylammonium cyanide (TBA-CN) to afford nitrile **15** in 49% yield alongside the eliminated alkene **14** (18%). Several protocols for reduction of the nitrile function were investigated but the NiCl_2_·6H_2_O/NaBH_4_ system in the presence of di-*tert*-butyl dicarbonate^53^ was found to be the best. This resulted in a mixture of the protected and the free amine which was treated once again with di-*tert*-butyl dicarbonate under conditions that would also cleave the pivaloyl ester. This gave **16** in 51% yield over two steps. The liberated primary alcohol was next mesylated with mesyl chloride before removal of the Boc-group with trifluoroacetic acid (TFA), then the compound was cyclized by heating in the presence of base to give the benzylated IGF **17** in 63% yield over three steps. The benzyl ethers were finally removed by catalytic hydrogenolysis over Pearlman's catalyst to give IGF (**1**) in 65% yield.

Iso-*galacto*-fagomine lactam (IGL, **3**)^54^ was prepared from primary alcohol **16** by first TEMPO-oxidation to lactam **18** in 74% yield followed by protecting group removal of first benzyl ethers using catalytic hydrogenolysis and then Boc-removal by TFA to give IGL (**3**) in 68% yield over two steps. Pyrrolidine DIL **4** was prepared from the diol **11** through a mesylation, cyclization with benzylamine^55^ and protecting group removal sequence as shown in Fig. 2B.

The final compound (AGF, **2**)^56^ was also prepared from key diol intermediate **11** (Fig. 2B). First, TEMPO-oxidation gave lactone **21** in 70% yield, which later could be reduced to l-ribofuranose derivative **22** by DIBAL. Reductive amination with *tert*-butyl carbazate next afforded hydrazine **23** in 64% yield from lactone **21**. As inspired by the synthesis of azafagomine from l-xylose,^57^ the second N-atom of hydrazide **23** was chemoselectively acetylated with acetic anhydride and the remaining secondary alcohol mesylated to give **24**. Boc-removal by TFA followed by cyclization in nitromethane gave hexahydropyridazine **25** in 73% over four steps from **23**. The protecting groups were then removed by catalytic hydrogenolysis over Pearlman's catalyst followed by hydrazide hydrolysis to give AGF (**2**) in 54% yield over two steps.

### Azasugar derivatives are competitive inhibitors of GALC

**Fig. 3:**
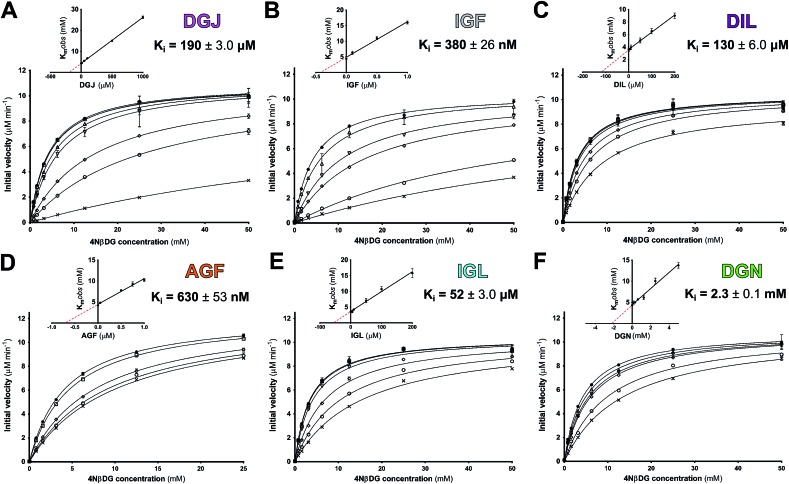
Competitive inhibition kinetics of *galacto*-configured azasugars. Plots of GALC initial velocity *vs.* substrate concentration with (A) DGJ, (B) IGF, (C) DIL, (D) AGF, (E) IGL and (F) DGN at concentrations encompassing the *K*_i_ (see ESI†). *Inset* plots of *K*_m^obs^_ *vs.* concentration showing –*K*_i_ as the *X*-intercept. SEM error bars are shown.

Inhibitory kinetics were determined for all molecules at the optimal pH of GALC, pH 4.6, using enzyme activity assays monitoring the processing of chromogenic substrate 4-nitrophenyl-β-d-galactopyranoside (4NβDG) as described previously.^11^ These assays were performed under steady-state conditions to allow determination of inhibitory constants (*K*_i_ values). All molecules were competitive inhibitors of GALC (Fig. 3) indicating that they specifically bind the active site and inhibited GALC with *K*_i_ values ranging from 190 μM down to 380 nM. Despite the similar chemical structures of the molecules tested, piperidine IGF and hydrazine AGF were both greater than 80 times more potent inhibitors of GALC than pyrrolidine DIL and lactam IGL. However, DIL and IGL were still two- and four-fold better than DGJ as inhibitors of GALC, respectively. Although the azasugar portion of nortropane DGN and IGF are very similar, DGN was a very weak inhibitor with a *K*_i_ of only 2.3 mM (Fig. 3F). The weakness of this inhibition is demonstrated by a *K*_i_ of 7.2 mM for the reaction product galactose.

### GALC is stabilized by binding azasugars

**Fig. 4:**
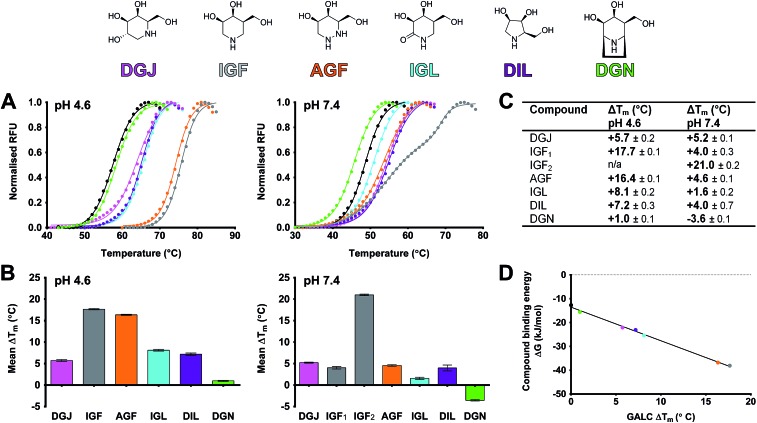
Stabilization of GALC by azasugar compounds. (A) Melt curves of GALC alone (black) and with 5.0 mM compound (chemical diagrams and coloring as above). Experiments were performed in sodium acetate, pH 4.6 and PBS, pH 7.4. (B) Bar graph showing the mean Δ*T*_m_ ± SEM conferred by 5.0 mM compound at pH 4.6 and pH 7.4. Experiments were performed in triplicate. (C) Values for mean Δ*T*_m_ ± SEM are tabulated. (D) Relationship between free energy of compound binding (Δ*G*) and GALC stabilization Δ*T*_m_ at pH 4.6. Δ*G* values for each compound were calculated from the measured *K*_i_ (Fig. 3) using the equation Δ*G* = *RT* ln *K*_i_, where *R* is the gas constant (8.31 J K^–1^ mol^–1^) and *T* is absolute temperature (310 K).

Candidate molecules for pharmacological chaperone activity need to not only bind GALC but should increase its global stability.^58,59^ Differential scanning fluorimetry (DSF) was used to monitor the thermal denaturation of GALC in the absence and presence of PCT candidates.^60^ The melting temperature (*T*_m_) is the inflection point of the sigmoidal melt curve and increased *T*_m_ indicates increased global stability. DSF experiments were carried out at two pH values equivalent to the two different cellular compartments where GALC folds and is active: the ER (pH 7.4) and the lysosome (pH 4.6), respectively (Fig. 4A). Notably, GALC alone is more stable at pH 4.6 (*T*_m_ = 57.3 °C) than pH 7.4 (*T*_m_ = 50.5 °C) a feature likely to be common amongst proteins optimized to function in acidic cellular compartments. All azasugars tested increase the global stability of wild-type GALC at both pHs except nortropane DGN, which destabilized GALC at pH 7.4 (Fig. 4A–C). DSF experiments performed with several concentrations of each azasugar revealed that stabilization is dose-dependent. Concentrations of 50 μM increase GALC *T*_m_, whilst higher concentrations (up to 10 mM) confer more than 20 °C stabilization to the wild-type enzyme (Fig. S1†).

At pH 4.6, 5 mM DGJ, pyrrolidine DIL and lactam IGL confer significant stabilization of GALC increasing the *T*_m_ of the wild-type enzyme by 6–8 degrees, while piperidine IGF and hydrazine AGF increase the *T*_m_ by an additional 8–9 degrees. The greater stabilization of GALC by IGF and AGF *versus* DGJ, DIL and IGL is consistent with their significantly greater inhibition of GALC. Indeed, the use of DSF measurements to monitor binding strength is validated by the linear relationship of *T*_m_ with Δ*G* calculated using the *K*_i_ values determined at pH 4.6 (Fig. 4D). Thus, despite not being able to measure *K*_i_ values at pH 7.4 due to negligible activity of GALC at this pH^11^ we can reliably monitor binding using the *T*_m_ from DSF measurements. At pH 7.4 the stability conferred by these azasugar compounds ranges from 1.6 to 5.2 degrees except for IGF. At this pH, IGF confers a double sigmoid melt curve (Fig. 4A) suggesting two populations, one of which confers significantly greater thermal stability (Δ*T*_m1_ = 4 °C *versus* Δ*T*_m2_ = 21 °C) and therefore higher binding affinity. The double melt curve observed for IGF is only apparent at high concentrations at pH 7.4 (discussed below).

Non-azasugars such as galactose and 2-deoxy-galactose confer no stabilization to GALC even at high concentrations (50 mM, Fig. S2†). Specificity for galactose-configured inhibitors was confirmed by testing stability in the presence of up to 10 mM of the glucose-configured 1-deoxynojirimycin (DNJ), which was unable to stabilize GALC at either pH (Fig. S2†). No change in *T*_m_ was observed at either pH for α-lobeline (data not shown).

### Azasugars bind specifically to the active site of GALC

**Fig. 5:**
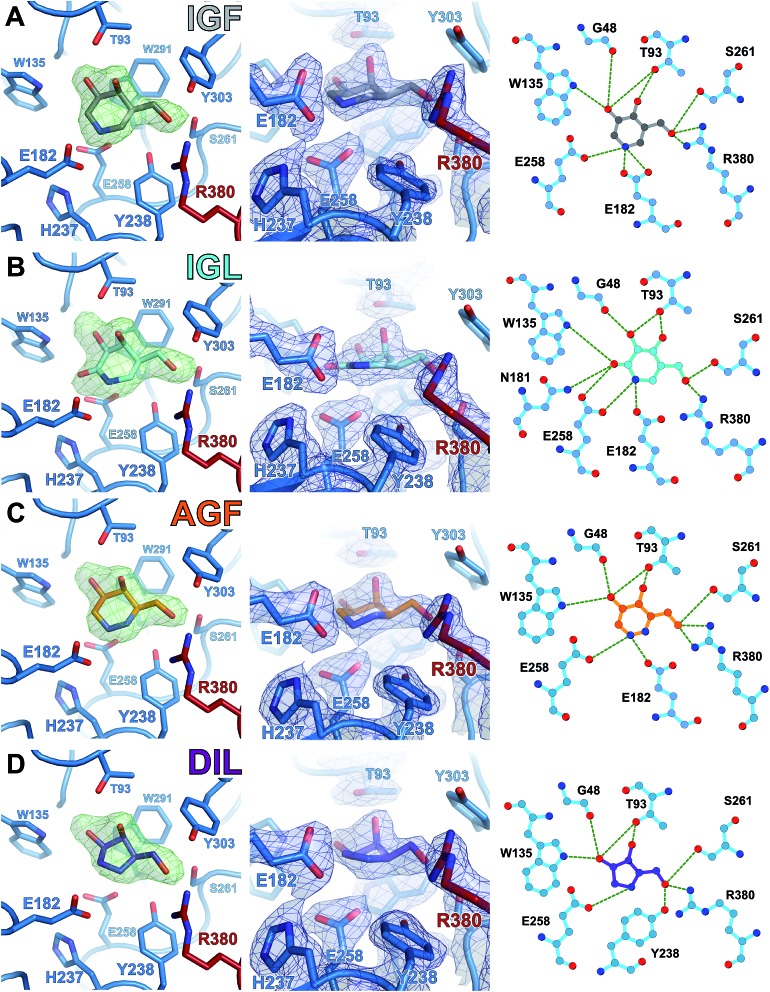
Structures of chaperone molecules bound in the GALC active site. X-ray crystal structures of (A) IGF, (B) IGL, (C) AGF and (D) DIL bound in the GALC active site. Compounds are colored as indicated. (*Left panel*) Unbiased difference electron density maps (*F*_O_ – *F*_C_, 3.0*σ*, green) before ligand modelling at the active site of GALC. (*Centre panel*) Detail of the GALC active site with bound ligand, showing active site residues (sticks) and refined 2*mF*_O_ – *DF*_C_ electron density contoured at 0.25 e Å^–3^ (blue). (*Right panel*) Schematic representation of hydrogen bond interactions (green dashed lines) between GALC and ligands.

**Fig. 6:**
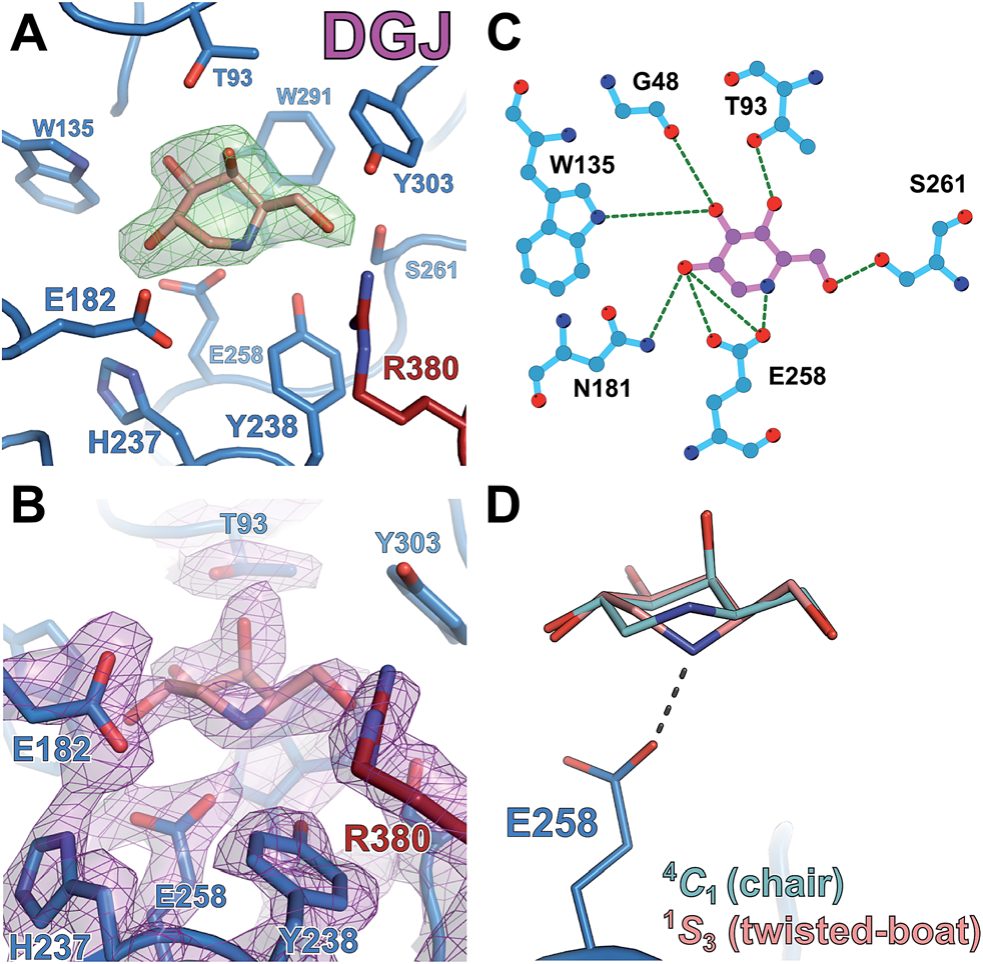
DGJ binds the active site in a distorted ^1^ *S*_3_ conformation. (A) Unbiased difference electron density map (*F*_O_ – *F*_C_, 3.0*σ*, green) before DGJ ligand refinement. (B) GALC active site with bound ligand, showing active site residues (sticks) and refined, feature-enhanced electron density map (0.65 e Å^–3^, purple). (C) Schematic representation of hydrogen bond interactions (green lines) between GALC and DGJ. (D) Adoption of the ^1^ *S*_3_ twisted-boat conformation allows DGJ to form a hydrogen bond with the nucleophile E258.

**Fig. 7:**
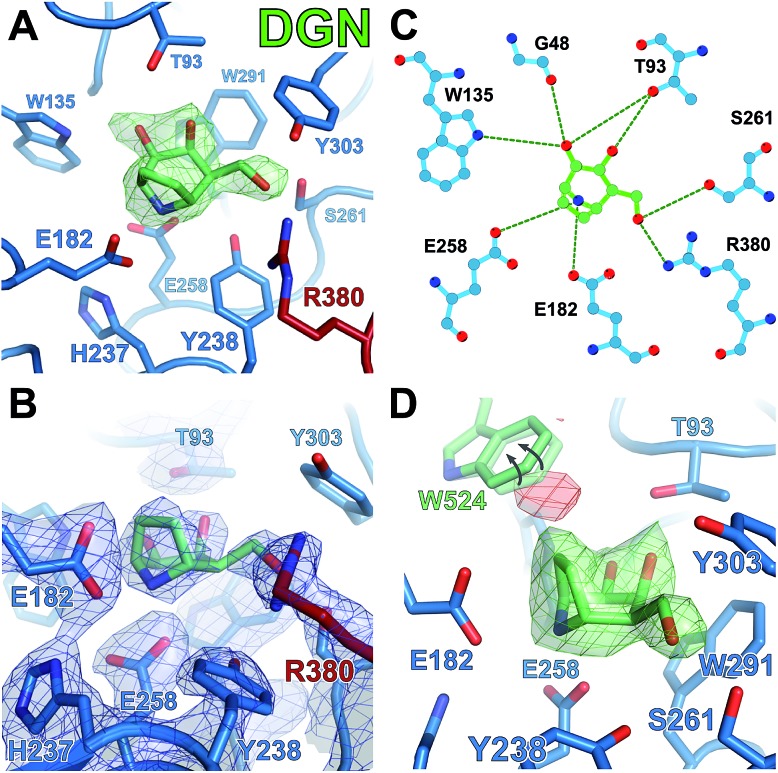
DGN destabilizes GALC due to a steric clash in the active site. (A) Unbiased difference electron density map (*F*_O_ – *F*_C_, 3.0*σ*, green) before DGN ligand refinement. (B) GALC active site with bound ligand, showing active site residues (sticks) and refined 2*mF*_O_ – *DF*_C_ electron density map (0.25 e Å^–3^, blue). (C) Schematic representation of hydrogen bond interactions (green lines) between GALC and DGN. (D) Unbiased difference electron density (*F*_O_ – *F*_C_; +3.0*σ* green, –3.0*σ* red) illustrating the steric clash between the ethylene bridge of DGN and W524 of the GALC lectin domain.

To gain atomic insight into the differences in inhibition and stabilization of GALC by these molecules, GALC crystals were soaked with each of the relevant compounds and X-ray diffraction data were collected (ESI Table 1†). All molecules bind in the active site of GALC and the conformations of each ligand were determined by modelling into unbiased *F*_O_ – *F*_C_ electron density (Fig. 5, left panels; Fig. 6A and 7A). All molecules form extensive hydrogen bonding networks with several GALC active site residues (Fig. 5, central and right panels; Fig. 6B and C and Fig. 7B and C). Careful analysis of the electron density maps verified that there are no secondary binding sites for any of the small molecules, ruling out allosteric contributions to stabilization.

All compounds bind in the same orientation as reaction product β-d-galactose,^11^ forming similar hydrogen bonding interactions between ligand 3-OH, 4-OH and 6-OH substituents, and side chains of active site residues W135, G48, T93, S261 and R380. DSF measurements with galactose confirm that these interactions alone are unable to confer stabilization to GALC (Fig. S2†). Only lactam IGL and DGJ possess a functional group on the ring corresponding to C2 of the substrate and in both these structures this group is able to form additional hydrogen bonds with N181 and E258 (Fig. 5B and 6C). Despite these additional interactions DGJ and IGL do not confer greater global stabilization than pyrrolidine DIL. These data combined suggest that formation of hydrogen bonds is not the primary factor contributing to the global stabilization of GALC.

A unique structural feature absent from galactose but shared by all tested compounds is the formation of a hydrogen bond/salt bridge between the heterocyclic nitrogen atom and a side chain O^ε^ atom of active site nucleophile E258. Due to the restraints on the ligand ring conformation for DIL and IGL this hydrogen bond is longer, and thus weaker, for these molecules (2.9 and 3.0 Å respectively, compared with ∼2.6 Å for IGF; Fig. 5). Despite the alternative position of the inhibitor nitrogen of DGJ, this molecule can maintain an interaction with E258 by adopting a ^1^ *S*_3_ twisted-boat conformation within the active site (Fig. 6D). This conformation is not the lowest energy conformation for DGJ in solution but was confirmed by analysis of feature-enhanced maps (FEM) that allow for interpretation of both strong and very weak signals at the same contouring level (Fig. 6B). Furthermore, quantum chemical calculations of the ligand and active site residues were used to generate minimized geometries and determine absolute energy differences between different local minima. This analysis revealed that within the context of the active site, the ^1^ *S*_3_ twisted-boat conformation is favoured by 14 kcal mol^–1^ over the chair conformation for DGJ.

Hydrazine AGF, with two nitrogen atoms in the ring, is potentially capable of forming hydrogen bonds with E258 *via* either nitrogen. Guided by the unbiased difference density and by the FEM maps, AGF was modelled in the ^4^ *C*_1_ chair conformation, very similar to that seen for piperidine IGF (Fig. 5A and C). Like IGF and AGF, IGL (Fig. 5B) contains a nitrogen atom at the C1 position equivalent to the anomeric carbon of the natural substrate. In these three compounds, this atom is capable of forming direct interactions with both E182 and E258. However the planar lactam group present in IGL restricts the ring conformation such that it cannot adopt the ^4^ *C*_1_ conformation resulting in weaker binding due to the longer bonding/interaction distances. Due to the position of the nitrogen in DGJ and the orientation of DIL in the active site neither of these molecules can form direct hydrogen bonds with E182 in the active site.

In order to understand how nortropane DGN may be destabilizing GALC, crystals were also soaked with this small molecule. Interestingly, DGN can bind the active site of GALC but in order to fit the binding pocket, residue W524 must alter conformation (Fig. 7D). W524 belongs to the lectin domain, helps form the shape of the binding pocket and does not normally move even to accommodate substrate possessing a bulky aglycon group.^11^ The ethylene bridge is designed to stabilize the ^4^ *C*_1_ conformation but due to its orientation in the tight active site, it forces the ring nitrogen down into very close proximity (2.3 Å) to the O^ε^ atom of E258. The combination of this steric clash and active site movements induces a distortion of the ring towards an *E*_1_ conformation.

To examine whether other residues in the active site can move to accommodate different iminosugars, DNJ, the *gluco*-configured congener of DGJ, was tested for active site binding. Despite overnight crystal soaking with 20 mM DNJ, no interpretable difference density was observed in the active site. This result is in agreement with the lack of thermal stabilization conferred by 50 mM DNJ in solution (Fig. S2†). The previously identified PCT candidate α-lobeline was also soaked into GALC crystals, but was not observed to bind (data not shown).

### GALC stabilization is dependent on the active site nucleophile

**Fig. 8:**
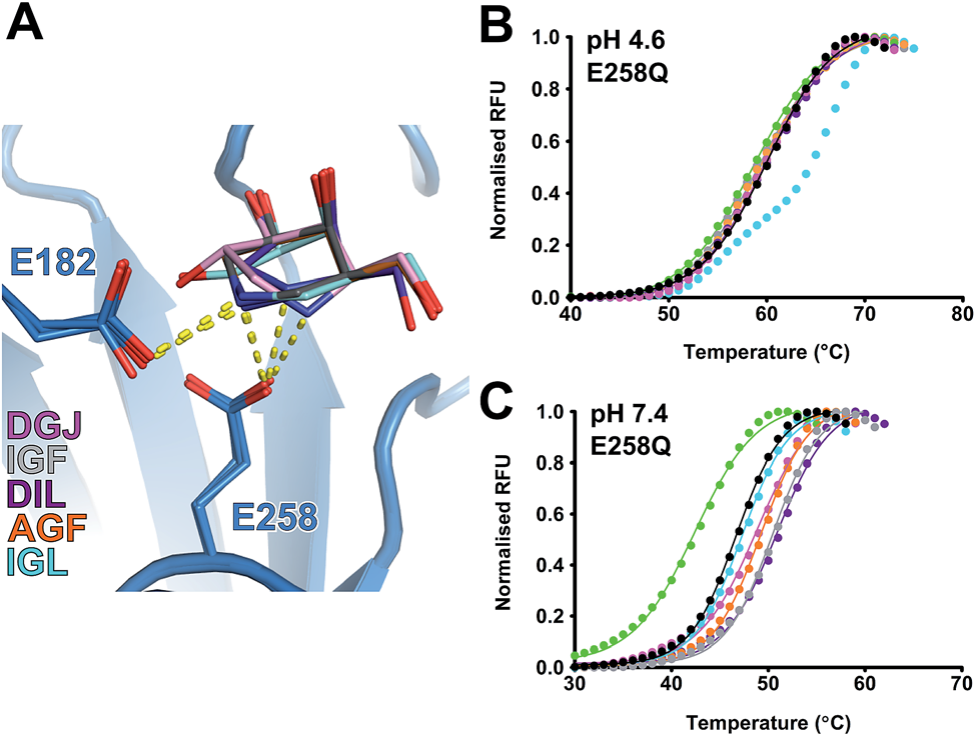
Azasugar interaction with E258 is essential for optimal GALC stabilization. (A) Alignment of azasugar compounds bound in the GALC active site. Refined structures are overlaid, colored as indicated. Hydrogen bonds to catalytic residues E258 and E182 are shown (yellow lines). (B and C) Overlaid melt curves of GALC E258Q alone (black) and with 10 mM compound (colored as above). Experiments were performed in (B) sodium acetate, pH 4.6 and (C) PBS, pH 7.4.

All molecules form stabilizing hydrogen bonds with the side chain of E258 in the active site (Fig. 8A). E258 is the catalytic nucleophile and thus possesses a negative charge at both pH 7.4 and 4.6. At low pH, most of the small molecules are protonated and so possess a positive charge. Thus E258 O^ε2^ could be forming a tight electrostatic interaction with the charged nitrogen in the ring of these molecules. Mutation of E258 to glutamine allows for the maintenance of a hydrogen bond but not an electrostatic interaction. We expressed and purified the E258Q mutant and repeated the DSF experiments at both pH 4.6 and pH 7.4. The importance of this interaction between E258 and the candidate chaperone molecules is highlighted by the complete loss of stabilization of GALC E258Q at pH 4.6 by all molecules except lactam IGL, which is incapable of forming a salt bridge (Fig. 8B). At pH 7.4, the global stability conferred by piperidine IGF to the E258Q mutant has lost the double sigmoid features seen for the wild-type GALC at pH 7.4 (Fig. 4A and 8C). Specifically, the higher *T*_m2_ of IGF is abolished indicating that it is the interaction with E258 that is mediating the greater stabilization at this pH. To further understand the nature of the GALC–IGF interaction that is conferring the double melt curve we carried out DSF experiments at a range of pH values. The different melting temperatures (*T*_m1_ and *T*_m2_) are pH dependent and the biphasic nature of the melt curve alters with pH (Fig. S3†). At higher pH, *T*_m2_ is no longer apparent with IGF only conferring a modest thermal stabilization to GALC, while at lower pH, *T*_m2_ is the most apparent species. Between pH 6.8 and pH 8.8 IGF transitions from a highly stabilizing molecule to a minimally stabilizing molecule. This suggests that the critical stabilizing property of IGF is an electrostatic interaction between the positively charged nitrogen in the IGF ring and the negatively charged nucleophile E258 in the active site.

Buffer strength and ionic charge alter the biphasic nature of GALC stabilization by IGF (Fig. S4†). Negatively charged ions in the buffer reduce IGF-mediated stabilization of GALC potentially by sequestering the positively charged IGF molecules in solution. These data support the hypothesis that it is the charged IGF molecules that confer greatest stabilization at pH 7.4 while the uncharged species contributes relatively little stabilization. The p*K*_aH_ of IGF is 8.8 so the majority of the IGF molecules (96%) will possess a positive charge at pH 7.4. However, hydrazine AGF has a p*K*_aH_ of 5.7 meaning that there will be far fewer molecules (2%) possessing a positive charge at pH 7.4.^61^ For AGF, the phosphate buffer was masking this smaller stabilization effect and DSF experiments in Tris buffer identify a biphasic melt curve for AGF that, in a similar way to IGF, is pH dependent (Fig. S5†). This identifies that it is the positively charged species that is mediating the greater stabilization of GALC and that even a small proportion of molecules can confer a significant effect. However, it also illustrates that the buffer components, specifically negatively charged ions, have the potential to mask this effect.

## Discussion

### Characteristics of different azasugar PC candidates

Here we present the synthesis of a series of azasugars from a common intermediate and their characterization as potential pharmacological chaperone candidates for Krabbe disease. We have determined accurate *K*_i_ values and validated DSF measurements as a measure of global stabilization of GALC. We have identified significant differences in the ability of each molecule to inhibit and stabilize GALC and dissected the role of specific chemical groups in order to determine the mechanisms by which this is conferred. We demonstrate the critical role of the catalytic nucleophile E258 in the mechanism of GALC stabilization by azasugar PC candidates. Mutation of E258 to glutamine results in almost complete lack of stabilization at pH 4.6 and pH 7.4. Hydrazine AGF and piperidine IGF-mediated GALC stabilization is primarily conferred by electrostatic interactions between the positively charged heterocyclic nitrogen atom and the negatively charged side chain of E258. The residual stabilization observed of E258Q at pH 7.4 is very likely mediated by an electrostatic interaction with the catalytic acid/base E182 which would be negatively charged at pH 7.4 unlike at pH 4.6 where it has to operate close to its p*K*_a_. Stabilization of GALC at different pH-values and under different buffer conditions highlights the importance of these electrostatic interactions as the sequestration of charged IGF and AGF species by buffer components modifies the stabilization profile of GALC. These data highlight both the importance of the p*K*_aH_ of the stabilizing ligand and the local ionic environment for the efficacy of these molecules as PC candidates.

Despite lactam IGL possessing a heterocyclic nitrogen in the equivalent position as IGF and AGF, this molecule does not confer equivalent stabilization to GALC. Thus, the inability to possess a formal positive charge on this nitrogen and therefore the lack of a stabilizing salt bridge reduces the potential of IGL as a PC candidate. Our data also identifies that it is not only charge but also the position of this charge in the active site that is crucial for stabilization of GALC. Both pyrrolidine DIL and DGJ possess a positive charge on the heterocyclic nitrogen at pH 4.6 but neither of these molecules confers equivalent stabilization as IGF and AGF. Thus position of the ring nitrogen directly adjacent to the negatively charged catalytic residues is critically important for achieving the tightest binding, and the maximum possible stabilization effect.

Of the aza/iminosugars tested, DGJ and nortropane DGN are the weakest inhibitors. Crystallographic data and quantum chemical calculations provide evidence that both must alter conformation from the low energy ^4^ *C*_1_ chair in order to bind the GALC active site. These conformational changes may act as a barrier to tight binding and thus contribute to the higher *K*_i_ for these molecules. For DGN, binding to GALC requires remodelling of the active site pocket *via* movement of W524. Despite this apparent flexibility of the binding pocket there was a considerable penalty to this binding as illustrated by the almost complete inability of DGN to inhibit GALC activity and its inability to stabilize, and indeed sometimes destabilize GALC. However, this flexibility of the active site is limited as glucose-based analogs do not inhibit, stabilize or bind GALC, identifying that specificity for galactose-based molecules, conferred by W291 near the sugar C4 atom,^44^ is retained.

### Future development and applications

In order to carry out the range of structural and biochemical assays described in this study we have used mouse GALC as a proxy for the human protein. The overall sequence identity between mouse and human GALC is 83% and all active site residues are fully conserved making conclusions drawn from this work directly relevant to human Krabbe disease. The structural and biochemical data described here provides a clear understanding of the mechanism of binding to the active site and how this interaction confers stabilization to the enzyme. Previous work with glucocerebrosidase had identified noeurostegine as a potential PC for Gaucher's disease conferring increased activity equivalent to that of isofagomine.^41^ However, due to the novel domain architecture of GALC, DGN clashes with the C-terminal lectin domain that forms part of the binding pocket, meaning the ethylene bridge modification will not be beneficial for future PCT development. Similarly, fluorine derivatives described previously as glucosidase inhibitors will decrease the p*K*_aH_ of the amine group and thus reduce their capacity to bind GALC at the higher pH of the ER.^62^ Additional aglycon groups are unlikely to confer significant enhancement to the chaperoning properties of these molecules due to the shallow shape of the binding pocket, as was observed in recent work with DGJ derivatives.^45^ This work has identified those characteristics that are required for PC specificity and pH-dependent affinity for GALC.

PCT relies on residual enzyme activity of the mutated protein and patients for whom this is not the case, such as those homozygous for the 30 kb deletion, will require alternative approaches. In the twitcher mouse model of Krabbe disease, which phenotypically mimics the 30 kb deletion, peripheral and intracerebral administration of mGALC significantly increased lifespan, improved motor function and reduced psychosine accumulation.^63,64^ Although ERT has not yet been clinically developed for Krabbe disease, recent studies have explored strategies for improving delivery of peripherally infused enzyme to the brain and CNS.^65,66^ Future development of ERT for Krabbe disease will benefit from the identification of PC candidates as equivalent molecules have been shown to possess considerable value as stabilizing agents for administered recombinant enzymes in Gaucher and Pompe disease.^67–69^ Furthermore, PCs have also been proposed to reduce hypersensitive immune responses induced by administration of protein denatured during storage.^70,71^ Thus in addition to their potential as PC candidates, the small molecules characterized in this work may prove valuable in future ERT development programmes using the wild-type GALC.

## Experimental section

### Small molecule synthesis

For full experimental description of syntheses of iso-*galacto*-fagomine (IGF), aza-*galacto*-fagomine (AGF), iso-*galacto*-fagomine lactam (IGL) and dideoxy-imino-lyxitol (DIL), see ESI.† The synthesis of 2-deoxy-*galacto*-noeurostegine (DGN) will be published elsewhere.

### Small-molecule inhibition kinetics: *K*_i_ determination

GALC protein was expressed and purified as described previously in ref. 11 and 44. Steady-state kinetic experiments were performed as described in ref. 11, using 3.18 nM GALC and small molecules at six concentrations covering a range that encompassed their inhibitory strength. *K*_i_ values were obtained from plots of initial velocity against substrate concentration by curve-fitting a competitive inhibition model using GraphPad Prism version 5.0. –*K*_i_ is represented graphically as the *X*-intercept of the linear plot of *K*_m^obs^_ against small molecule concentration.

### Differential scanning fluorimetry (DSF)

DSF experiments were performed in 48-well plates, 50 μL reactions comprised 5.0 μg GALC and 5× SyPRO Orange dye in either PBS pH 7.4, 20 mM Tris pH 7.4 or 20 mM sodium acetate pH 4.6. Small molecules were added to final concentration of 10, 5.0, 1.0, 0.5, 0.1 and 0.05 mM. The melting curve was performed using a Bio-Rad MiniOpticon RT-PCR thermal cycler between 20 °C and 95 °C in 1 °C steps, with 20 s equilibration time per step. SyPRO fluorescence was monitored on the HEX channel. The melting temperature (*T*_m_) was the inflexion point of the sigmoidal curve obtained by curve fitting using DSF analysis scripts^60^ and GraphPad Prism version 5.0. Stabilization is expressed as the Δ*T*_m_ compared to a protein-only control. All experiments were performed in triplicate.

### GALC crystallization and small molecule soaks

GALC protein was crystallized as described previously.^11^ Small molecules were dissolved in water at 100 mM and diluted to 20 mM in crystallization reservoir solution. Soaks were started by the addition of 0.5 μL ligand solution directly to the crystallization drop. Immediately after starting the soak, 0.5 μL perfluoropolyether oil (Hampton Research) was layered over the drop as a cryo-protectant. Crystals were soaked for between 30 seconds and 5 minutes before removal through the oil and flash-cooling in liquid nitrogen. For small molecules that did not appear to bind in the crystal structures (α-lobeline and DNJ) soaks were repeated for 1 hour and overnight.

### X-ray data collection, structure determination and refinement

Diffraction datasets were recorded on beamlines I04-1 and I02 at Diamond Light Source using Pilatus 2M and 6M detectors (Dectris). Data were collected at 100 K at *λ* = 0.98 Å. Data collection statistics are in ESI Table 1.† Diffraction data were integrated and scaled using MOSFLM/AIMLESS^72,73^ or XDS/XSCALE^74^ *via* the xia2 automated data processing pipeline.^75^ Resolution cut-off was decided by CC1/2 value of >0.5 and *I*/*σI* of >1.5 in the outer resolution shell. The same set of “FreeR” reflections were excluded from refinement as those used previously when refining the un-liganded GALC structure. Unbiased electron density maps of GALC–ligand complexes were generated after rigid-body refinement using phenix.refine^76–78^ to position the un-liganded GALC model (PDB ID ; 3ZR5) in the unit cell. PDB files and CIF restraints for ligands were generated from SMILES strings using eLBOW^79^ or GRADE (Global Phasing). All further refinement was performed iteratively using COOT^80^ and phenix.refine. Model geometry was evaluated with MolProbity^81^ throughout the refinement process. Feature enhanced maps (FEM) were calculated using Phenix.^82^ Structural figures were rendered using PyMOL (Schrödinger LLC). For clarity, electron density maps are displayed within 2.0 Å of highlighted residues. Hydrogen bonding representations were created using LIGPLOT+.^83^

### Quantum chemistry (QC) calculations

For QC calculations a reduced model consisting of the refined ligand and the binding site was used. A python script created a PDB file containing protein residues within 5 Å of the ligand and a GAMESS^84,85^ input file. This was implemented directly and using python scripts with wrappers available in eLBOW.^79^ QC is an all electron method so hydrogens were added to the model and their geometry was optimized using the B3LYP/6-31(d,p) method and basis set. These geometries were used with all atoms of the ligand and the hydrogens of the reduced model were optimized. The resulting geometries were compared with refined models to validate configuration and orientation.

## Conclusion

In summary, we have identified the mechanism by which a series of azasugar molecules bind to and stabilize the lysosomal hydrolase GALC. These molecules bind specifically in the active site pocket where both the position of the ring nitrogen and its positive charge determine increased stabilization of GALC. Our data confirm that IGF and AGF are the optimal scaffolds for exploiting this charge-mediated stabilization of GALC and that these molecules have great potential for future PCT and ERT for human Krabbe disease.
