# Supplementary material for: Azasugar inhibitors as pharmacological chaperones for Krabbe disease
Source: Chem Sci. 2015 Mar 23;6(5):3075–86. doi: 10.1039/c5sc00754b (PMC4445328; doi:10.1039/c5sc00754b)
Supplement: Supplementary file 1 [file SC-006-C5SC00754B-s001.pdf]

Electronic Supplementary Material (ESI) for Chemical Science.
This journal is © The Royal Society of Chemistry 2015

The Role of Azasugar Inhibitors as Pharmacological Chaperones for Krabbe Disease

Chris H. Hill*[1], Agnete H. Viuff*[2], Sam J. Spratley[1], Stéphane Salamone[2], Stig H. Christensen[2], Randy J. Read[1], Nigel W. Moriarty[3], Henrik H. Jensen[#2] and Janet E. Deane[#1]

Affiliations:

[1] Department of Haematology, Cambridge Institute for Medical Research, University of Cambridge, Cambridge CB2 0XY, UK

[2] Department of Chemistry, Aarhus University, Langelandsgade 140, 8000 Aarhus C., Denmark

[3] Physical Biosciences Division, Lawrence Berkeley National Laboratory, Berkeley, CA 94720 USA

*Joint first authors, # Joint corresponding authors

Content

General methods S1

Apparatus S2

Abbreviations S2

Synthesis S2

NMR spectra S18

Small-molecule inhibition kinetics S49

Supporting Table S1 .... S50

Figure S1-S5 .... S51

References S56

General methods

All reactions with air- and moisture sensitive compounds were conducted in an atmosphere of nitrogen or argon. Dichloromethane, toluene, acetonitrile and THF were dried over aluminum oxide via an MBraun SPS-800 solvent purification system, other dry solvents were dried over molecular sieves. Evaporation of solvents was done under reduced pressure at 40 °C. Flash column chromatography was carried out with a Merck silica gel (230-400 Mesh). TLC analysis was carried out on silica gel on aluminum foil (Merck Kieselgel 60 F_{254}). TLC plates were visualized by using ceric sulfate/ammonium molybdate in 10 % H_2SO_4 or ninhydrin in *n*-butanol/acetic acid and successive heating to dryness.

Apparatus

NMR spectra were recorded on a Varian Mercury 400 spectrometer. ^{1}H-, gCOSY- and gHMQC-NMR were recorded at 400 MHz and ^{13}C NMR and DEPT-135 at 100 MHz. Chemical shifts (δ) are given in ppm relative to the residual solvent signals ($CDCl_3$ (δ 7.26 ppm for proton and 77.16 ppm for carbon resonances) and D_2O (δ 4.79 ppm for proton). Assignment of NMR spectra is based on gCOSY, gHMQC and DEPT-135 experiments. Mass spectra were recorded on a Micromass LC-TOF spectrometer with positive electronspray ionization. Melting points were measured on a Büchi B-450, and are not corrected. Optical rotations were measured on an ADP440+ polarimeter and reported in units of deg·cm^{2}·g^{-1}. Concentrations are given in g/100mL.

Abbreviations

Aq.	Aqueous
Boc	*tert*-butyloxycarbonyl
DIBAl	Diisobutylaluminum hydride
DIPEA	*N,N*-Diisopropylethylamine
DMF	*N,N*-dimethylformamide
Ms	Methanesulfonyl
m-CPBA	*meta*-chloroperoxybenzoic acid
Piv	Pivaloyl
Sat.	Saturated
TBAF	Tetra-*n*-butylammonium fluoride
TBACN	Tetra-*n*-butylammonium cyanide
TEMPO	(2,2,6,6-Tetramethyl-piperidin-1-yl)oxyl
TFA	Trifluoroacetic acid
Tf	Trifluoromethanesulfonyl
THF	Tetrahydrofuran
TIPS	Triisopropylsilyl

Synthesis

2,3-*O*-isopropylidene-5-*O*-triisopropylsilyl-D-ribofuranose (6)

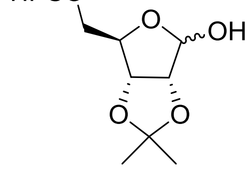

6

The isopropylidene protected D-ribose **5** (335 mg, 1.76 mmol) was dissolved in anhydrous dichloromethane (1.3 mL) and imidazole (184 mg, 2.70 mmol, 1.5 equiv.) and TIPSCl (0.42 mL, 1.96 mmol, 1.1 equiv.) were added at 0 °C. The reaction was stirred at 0 °C for 3h before allowed to warm to room temperature. After 1h

30 min, more TIPSCl (0.2 mL) was added and the mixture stirred overnight. The reaction was then quenched by addition of sat. aq. NH_4Cl and the aqueous layer extracted with dichloromethane. The combined organic phases were dried over $MgSO_4$, filtered and concentrated under reduced pressure. The crude product was purified by flash column chromatography (pentane/ethyl acetate 12:1) to give the product **6** (427 mg, 70%) as a 1:5.9 mixture of anomers.

R_f (pentane/ethyl acetate 15:1) 0.28; Data for major anomer: ^{1}H-NMR (400 MHz, $CDCl_3$): δ_H 5.27 (d, $J_{1,2}$ 11.6 Hz, 1H, H1), 4.80 (d, $J_{3,4}$ 5.9 Hz, 1H, H3), 4.74 (d, 1H, O*H*), 4.52 (d, 1H, H2), 4.33 (bt, 1H, H4), 3.83 (d, *J* 2.3 Hz, 2H, H5), 1.47 (s, 3H, C*H*$_3$), 1.31 (s, 3H, C*H*$_3$), 1.09 – 1.02 (m, 21H, C*H*(CH_3)$_2$, (C*H*$_3$)). ^{13}C-NMR (100 MHz, $CDCl_3$) δ_C 112.1, 103.4, 87.6, 87.3, 81.6, 77.5, 77.2, 76.8, 65.2, 26.6, 25.0, 18.0, 18.0, 11.9. HRMS(ESI): calcd. for $C_{17}H_{34}O_5SiNa$ 369.2063; found 369.2073.

1,4-di-*O*-benzyl-2,3-*O*-isopropylidene-5-*O*-triisopropylsilyl-D-ribitol (7)

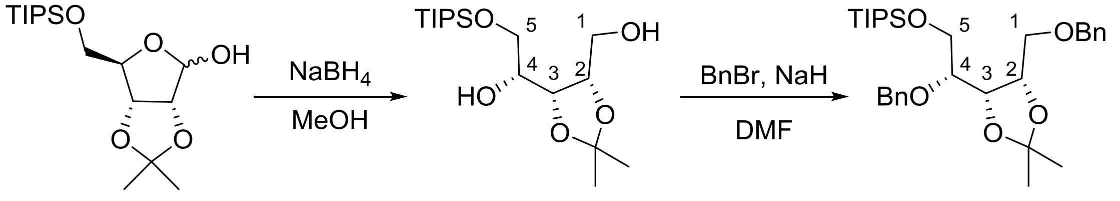

6 **26** **7**

2,3,-*O*-isopropylidene-5-*O*-triisopropylsilyl-D-ribitol (26)

Furanose **6** (4.11 g, 11.9 mmol) was dissolved under stirring in anhydrous methanol (40 mL) at 0 °C. Sodium borohydride (821 mg, 21.7 mmol, 1.8 equiv.) was added to the solution and the reaction mixture stirred at room temperature for 2h 30 min. The reaction was quenched by addition of a small piece of dry ice and stirred for 10 minutes. The solvent was removed under reduced pressure and the residue re-dissolved in ethyl acetate, washed with brine, dried over $MgSO_4$, filtered and concentrated under reduced pressure. This yielded the desired crude product **26** (4.01 g) as a colorless oil.

R_f (pentane/ethyl acetate 6:1) 0.19; $[\alpha]_D^{293K}$ +21.8 (*c* 1.0, $CHCl_3$); ^{1}H-NMR (400 MHz, $CDCl_3$): δ_H 4.36 (dt, 1H, $J_{1b,2}$ 11.3 Hz, $J_{2,1a}$ = $J_{2,3}$ 5.6 Hz, H2), 4.09 (dd, $J_{3,4}$ 9.3 Hz, 1H, H3), 3.93 (dd, 1H, $J_{5a,5b}$ 9.7 Hz, $J_{4,5a}$ 3.2 Hz, H5a), 3.92 - 3.89 (m, 1H, H1a), 3.83 - 3.72 (m, 3H, H1b, H4 and H5b), 3.18 (dd, 1H, $J_{1',OH}$ 9.0 Hz, $J_{1,OH}$ 4.9 Hz, O*H*1), 3.12 (d, 1H, $J_{4,OH}$ 4.5 Hz, O*H*4), 1.39 (s, 3H, C*H*$_3$), 1.32 (s, 3H, C*H*$_3$), 1.12 - 1.06 (m, 21 H, C*H*$_3$, -C*H*(CH_3)$_2$). ^{13}C-NMR (100 MHz, $CDCl_3$) δ_C 108.6 (*C*(CH_3)$_2$), 77.8 (C2), 76.7 (C3), 69.7 (C4), 64.7 (C5), 61.0 (C1), 28.0 (*C*H$_3$), 25.3 (*C*H$_3$), 18.0 (*C*H$_3$), 12.0 (*C*H(CH_3)$_2$). HRMS(ESI) calcd. for $C_{17}H_{36}O_5SiNa$ 371.2230; found 371.2232.

1,4-di-*O*-benzyl-2,3-*O*-isopropylidene-5-*O*-triisopropylsilyl-D-ribitol (7)

The crude diol **26** (4.01 g, 11.5 mmol) was dissolved in anhydrous DMF (70 mL) and cooled to 0 °C. BnBr (4.1 mL, 34.5 mmol, 3 equiv.) and NaH (60 %, 1.05 g, 26.3 mmol, 2.3 equiv.) were added under a nitrogen atmosphere. The reaction was stirred for 3 hours while slowly heating to room temperature. The reaction mixture was then diluted with ethyl acetate, washed with water, dried over $MgSO_4$, filtered and concentrated under reduced pressure. The crude product was purified by dry column vacuum chromatography[1] (0-9 % ethyl acetate in heptane over 10 steps) to yield the product **7** (6.09 g, 94 % over 2 steps)

R_f (pentane/ethyl acetate 30:1) 0.28; $[\alpha]_D^{293K}$ -18.4 (*c* 1.0, $CHCl_3$); ^{1}H-NMR (400 MHz, $CDCl_3$): δ_H 7.30 -7.12 (m, 10H, Ar*H*), 4.84 (d, 1H, *J* 11.1 Hz, C*H*HPh), 4.54 (d, 1H, *J* 12.4 Hz, C*H*HPh), 4.45 (d, 1H, CH*H*Ph), 4.39 - 4.34 (m, 1H, H2), 4.32 (d, 1H, CH*H*Ph), 4.13 (dd, 1H, $J_{3,4}$ 8.8 Hz, $J_{3,2}$ 6.2 Hz, H3), 4.09 (dd, 1H, $J_{5a,5b}$ 11.1 Hz, $J_{5a,4}$ 2.4 Hz, H5a), 3.82 (dd, 1H, $J_{5b,4}$ 5.3 Hz, H5b), 3.69 (dd, 1H, $J_{1a,1b}$ 10.2 Hz, $J_{1a,2}$ 2.9 Hz, H1a), 3.55 (ddd, 1H, H4), 3.47 (dd, 1H, H1b), 1.42 (s, 3H, CH_3), 1.30 (s, 3H, CH_3), 1.13 - 0.98 (m, 21 H, C*H*(CH_3)$_2$, CH_3). ^{13}C-NMR (100 MHz, $CDCl_3$): δ_C 138.4 (Ar), 138.2 (Ar), 128.4 - 127.9 (Ar), 127.8 (Ar), 127.8 (Ar), 108.5 (*C*(CH_3)$_2$), 78.5 (C4), 76.9 (C2), 75.0 (C3), 73.5 (*C*H_2Ph), 72.0 (*C*H_2Ph), 69.2 (C1), 64.2 (C5), 28.0 (*C*H_3), 25.5 (*C*H_3), 18.1 (*C*H_3), 12.4 (*C*H(CH_3)$_2$). HRMS(ESI) calcd. for $C_{31}H_{48}O_5SiNa$ 551.3169; found 551.3169.

1,4-di-*O*-benzyl-5-*O*-triisopropylsilyl-D-ribitol (8)

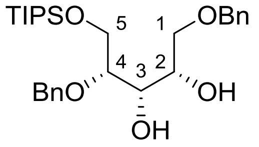

8

The isopropylidene protected compound **7** (472 mg, 890 µmol) was dissolved in dichloromethane (20 mL) and cooled to 0 °C before $FeCl_3 \cdot 6H_2O$ (805 mg, 2.98 mmol, 3.4 equiv.) was added. The mixture was stirred at 0 °C under a nitrogen atmosphere for 3 h before the reaction was quenched with sat. aq. $NaHCO_3$. The aqueous phase was extracted three times with dichloromethane. The combined organic phases were dried over $MgSO_4$, filtered and concentrated under reduced pressure. The resulting oil was purified by flash column chromatography (pentane/ethyll acetate 3:1 -> EtOAc) yielding the product **8** (335 mg, 77 %) as a clear oil.

R_f (pentane/ethyl acetate 6:1) 0.21; $[\alpha]_D^{293K}$ -20.6 (*c* 1.0, $CHCl_3$); ^{1}H-NMR (400 MHz, $CDCl_3$): δ_H 7.38-7. 27 (m, 10H, Ar*H*), 4.74 (d, 1H, *J* 11.7 Hz, C*H*HPh), 4.59 (d, 1H, CH*H*Ph), 4.56 (d, 1H, *J* 11.7 Hz, C*H*HPh), 4.52 (d, 1H, CH*H*Ph), 4.08 (dd, 1H, $J_{1a,1b}$ 10.7 Hz, $J_{1a,2}$ 4.8 Hz, H1a), 3.99 – 3.88 (m, 3H, H2, H3, H4), 3.73-3.61 (m, 3H, H1b, H5a, H5b), 1.19-1.01 (m, 21 H, CH_3, C*H*(CH_3)$_2$). ^{13}C-NMR (100 MHz, $CDCl_3$): δ_C 138.2 (Ar), 137.9 (Ar), 128.3 - 127.6 (Ar), 79.8, 73.4, 72.6, 72.3, 71.8, 70.9, 63.7, 17.9 (*C*H_3), 11.8 (*C*H(CH_3)$_2$). HRMS(ESI): calcd. for $C_{28}H_{44}O_5SiNa$ 511.2856; found 511.2852.

1,4-di-*O*-benzyl-2,3-*O*-benzylidene-5-*O*-triisopropylsilyl-D-ribitol (9)

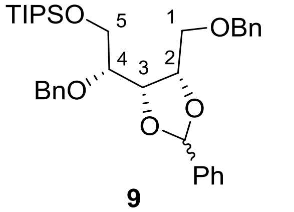

9

Diol **8** (477 mg, 976 µmol) was dissolved in anhydrous toluene (4.4 mL) under a nitrogen atmosphere before dry 10-camphorsulfonic acid (3.60 mg, 15.5 µmol, 0.16 equiv.) and benzaldehyde dimethyl acetal (0.16 mL, 1.07 mmol, 1.1 equiv.) were added. The mixture was stirred at 50 °C and 200 mbar on a rotary evaporator for 50 min. before the reaction was quenched with sat. aq. $NaHCO_3$, extracted once with dichloromethane, dried over $MgSO_4$, filtered and concentrated under reduced pressure. The crude product was purified by

flash column chromatography (pentane/ethyl acetate 40:1) to yield the product **9** as a 1:5 diastereomeric mixture (651 mg, 89 %), that could be partly separated by flash column chromatography.

Data for major diastereoisomer: R_f (pentane/ethyl acetate 40:1) 0.14; $[\alpha]_D^{293K}$ -24.6 (*c* 1.0, $CHCl_3$); ^{1}H-NMR (400 MHz, $CDCl_3$): δ_H 7.55-7.46 (m, 2H, Ar*H*), 7.39-7.19 (m, 13H, Ar*H*), 5.81 (s, 1H, C*H*Ph), 4.90 (d, 1H, *J* 11.2 Hz, C*H*HPh), 4.56 (d, 1H, *J* 12.2 Hz, C*H*HPh), 4.50 (d, 1H, CH*H*Ph), 4.48 (ddd, 1H, $J_{1b,2}$ 7.4 Hz, $J_{2,3}$ 6.4 Hz, $J_{1a,2}$ 3.0 Hz, H2), 4.41 (d, 1H, CH*H*Ph), 4.27 (dd, 1H, $J_{3,4}$ 8.8 Hz, H3), 4.15 (dd, 1H, $J_{5a,5b}$ 11.1 Hz, $J_{4,5a}$ 2.3 Hz, H5a), 3.89 (dd, 1H, $J_{4,5b}$ 5.3 Hz, H5b), 3.86 (dd, 1H, $J_{1a,1b}$ 10.5 Hz, H1b), 3.74 (ddd, 1H, H4), 3.64 (dd, 1H, H1), 1.15-1.01 (m, 21H, C*H*$_3$, C*H*(CH_3)$_2$). ^{13}C NMR (100 MHz, $CDCl_3$): δ_H 138.3 (Ar), 138.1 (Ar), 137.5 (Ar), 129.2-126.8 (Ar), 103.4 (Ph*C*H), 78.5 (C4), 78.1 (C2), 76.1 (C3), 73.5 (*C*H$_2$Ph), 72.1 (*C*H$_2$Ph), 69.1 (C1), 64.1 (C5), 18.1 (*C*H$_3$), 11.9 (*C*H(CH_3)$_2$). HRMS(ESI): calcd. for $C_{35}H_{48}O_5SiNa$ 599.3169; found 599.3175.

Data for minor diastereoisomer: R_f (pentane/ethyl acetate 40:1) 0.19. $[\alpha]_D^{293K}$ -16.8 (*c* 1.0, $CHCl_3$) ; ^{1}H-NMR (400 MHz, $CDCl_3$): δ_H 7.46-7.42 (m, 2H, Ar*H*), 7.37-7.20 (m, 13H, Ar*H*), 6.18 (s, 1H, C*H*Ph), 4.87 (d, 1H, *J* 11.2 Hz, C*H*HPh), 4.55 (s, 2H, C*H*$_2$Ph), 4.53 (dd, 1H, $J_{1a,2}$ 6.3 Hz, $J_{2,3}$ 3.0 Hz, H2), 4.37 (d, 1H, *J* 11.2 Hz, C*H*$_2$Ph), 4.28 (dd, 1H, $J_{3,4}$ 8.8 Hz, $J_{4,5b}$ 6.1 Hz, H4), 4.17 (dd, 1H, $J_{5a,5b}$ 11.1 Hz, $J_{4,5}$ 2.3 Hz, H5a), 3.91 (dd, 1H, H5b), 3.77 (dd, 1H, $J_{1a,1b}$ 8.9 Hz, $J_{1a,2}$ 7.4 Hz, H1a), 3.77 (dd, 1H, H3), 3.69 (dd, 1H, H1b), 1.12-0.99 (m, 21H, C*H*$_3$, C*H*(CH_3)$_2$). ^{13}C-NMR (100 MHz, $CDCl_3$): δ_C 139.5 (Ar), 138.4 (Ar), 138.2 (Ar), 128.9-126.3 (Ar), 103.2 (Ph*C*H), 78.4 (C3), 77.8 (C2), 75.4 (C4), 73.6 (*C*H$_2$Ph), 72.1 (*C*H$_2$Ph), 68.8 (C1), 64.1 (C5), 18.2 (*C*H$_3$), 12.1 (*C*H(CH_3)$_2$).

1,3,4-tri-*O*-benzyl-5-*O*-triisopropylsilyl-D-ribitol (10)

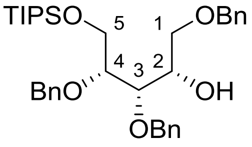

10

Benzylidene acetal **9** (901 mg, 1.57 mmol) was dissolved in anhydrous dichloromethane (11 mL), and the solution was cooled to 0 °C under a nitrogen atmosphere. DIBAL (1.0 M solution in toluene, 2.4 mL, 2.4 mmol, 1.5 equiv.) was added in a dropwise fashion. The mixture was stirred at 0 °C for 3h 30 min and then added more DIBAL (0.8 mL). Stirred overnight at 4 °C and before a few drops of methanol was added and the solution diluted with dichloromethane and washed with a Rochelle's salt solution. The organic phase was dried over $MgSO_4$, filtered and concentrated under reduced pressure to yield the crude product **10** (801 mg) as colorless oil.

R_f (pentane/ethyl acetate 12:1) 0.33; $[\alpha]_D^{293K}$ -2.2 (*c* 1.0, $CHCl_3$); ^{1}H-NMR (400 MHz, $CDCl_3$): δ_H 7.36-7.20 (m, 15 H, Ar*H*), 4.78 (d, 1H, *J* 11.7 Hz, C*H*HPh), 4.68 (d, 1H, *J* 11.4 Hz, C*H*HPh), 4.63 (d, 1H, CH*H*Ph), 4.56 (d, 1H, CH*H*Ph), 4.53 (d, 1H, *J* 12.0 Hz, C*H*HPh), 4.49 (d, 1H, CH*H*Ph), 4.08-3.99 (m, 2H, H1, H5a), 3.89-3.81 (m, 2H, H5b, H4), 3.77 (dd, 1H, *J* 6.8 Hz, 3.8 Hz, H3), 3.63-3.59 (m, 2H, H1a, H2), 2.93 (bd, 1H, $J_{OH,2}$ 4.8 Hz, 1H, O*H*), 1.11-1.02 (m, 21H, C*H*$_3$, C*H*(CH_3)$_2$). ^{13}C-NMR (100 MHz, $CDCl_3$): δ_C 138.6 (Ar), 138.5 (Ar), 138.2 (Ar), 128.4-127.6 (Ar), 81.0 (C4), 79.1 (C3), 73.6 (*C*H$_2$Ph), 73.4 (*C*H$_2$Ph), 73.0 (*C*H$_2$Ph), 71.3, 71.1 (C2, C1), 63.5 (C5), 18.1 (*C*H$_3$), 12.0 (*C*H(CH_3)$_2$). HRMS(ESI): calcd. for $C_{35}H_{50}O_5SiNa$ 601.3325; found 601.3331.

1,3,4-tri-*O*-benzyl-D-ribitol (11)

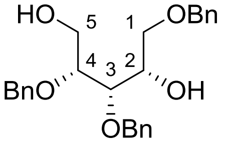

11

The crude TIPS-ether **10** (801 mg, 1.38 mmol) was dissolved in anhydrous THF (4 mL) and added TBAF 1 M in THF (1.4 mL, 1.4 mmol, 1 equiv.) under a nitrogen atmosphere. The reaction was stirred at room temperature overnight, and then quenched with water, diluted with ethyl acetate and washed with water and brine. The combined organic phases were dried over $MgSO_4$, filtered and concentrated under reduced pressure. The crude product was purified by flash column chromatography (pentane/ethyl acetate 3:2) to yield the product **11** as a colorless oil (481 mg, 72 % over 2 steps).

R_f (pentane/ethyl acetate 3:2) 0.33; $[\alpha]_D^{293K}$ -14.4 (*c* 1.0, $CHCl_3$); ^{1}H-NMR (400 MHz, $CDCl_3$) δ_H 7.32-7.15 (m, 15H, Ar*H*), 4.66 (d, 1H, *J* 11.2 Hz, C*H*HPh), 4.58 (d, 1H, *J* 11.6 Hz, C*H*HPh), 4.53 (d, 1H, CH*H*Ph), 4.52 (d, 1H, CH*H*Ph), 4.46 (d, 1H, *J* 12.0 Hz, C*H*HPh), 4.42 (d, 1H, CH*H*Ph), 3.94 (td, 1H, *J* 6.0 Hz, *J* 3.7 Hz, H2), 3.77 (dd, *J* 8.8 Hz, *J* 4.1 Hz, 2H, H5), 3.74 – 3.67 (m, H3, H4), 3.57-3.48 (m, 2H, H1), 2.26 (bs, 1H, O*H*). ^{13}C-NMR (100 MHz, $CDCl_3$) δ_C 138.1 (Ar), 138.1 (Ar), 137.9 (Ar), 128.6 (Ar), 128.5 (Ar), 128.5 (Ar), 128.2 (Ar), 128.0 (Ar), 128.0 (Ar), 127.9 (Ar), 79.5 (C3/C4), 79.4 (C4/C3), 74.1 (*C*H_2Ph), 73.5 (*C*H_2Ph), 72.1 (*C*H_2Ph), 71.2 (C1), 70.7 (C2), 61.1 (C5). HRMS(ESI): calcd. for $C_{26}H_{30}O_5Na$ 445.1991; found 445.1989.

1,3,4-tri-*O*-benzyl-5-*O*-pivaloyl-D-ribitol (12) and 1,3,4-tri-*O*-benzyl-2,5-di-*O*-pivaloyl-D-ribitol (30)

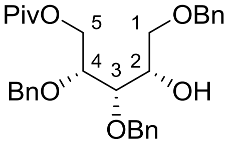

12

and

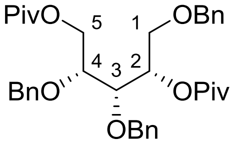

30

Diol **11** (2.80 g, 6.64 mmol) was dissolved in anhydrous pyridine (7.6 mL) and cooled to 0 °C. Pivaloyl chloride (0.82 mL, 6.66 mmol, 1 equiv.) was then added over 5 min under a nitrogen atmosphere. After 3 hours, more pivaloyl chloride (0.16 mL, 1.30 mmol, 0.2 equiv.) was added and after additional 30 min the reaction was quenched with water and stirred for 15 min. The mixture was then extracted with ethyl acetate, washed with 1M aq. hydrochloric acid, sat. aq. $NaHCO_3$ and brine, dried over $MgSO_4$, filtered and concentrated under reduced pressure. The crude product was purified by flash column chromatography (pentane/ethyl acetate 8:1) to yield the product **12** (2.80 g, 83 %) and the di-pivaloylated side product (**30**) (223 mg, 6 %).

R_f (pentane/ethyl acetate 8:1) 0.34; $[\alpha]_D^{293K}$ -6.1 (*c 1.0*, $CHCl_3$); ^{1}H-NMR (400 MHz, $CDCl_3$): δ_H 7.36-7.24 (m, 15H, Ar*H*), 4.72 (d, 1H, *J* 11.4 Hz, 2xC*H*HPh), 4.65-4.53 (m, 3H, C*H_2*Ph, H5a), 4.52 (d, *J* 11.9 Hz, 1H, C*H*HPh), 4.48 (d, 1H, CH*H*Ph), 4.25 (dd, 1H, $J_{5a,5b}$ 12.1 Hz, $J_{4,5b}$ 6.0 Hz, H5b), 4.01 (ddd, $J_{2,3}$ 6.9, $J_{2,1a}$ 5.4, $J_{2,1b}$ 3.8 Hz, 1H, H2), 3.96 (ddd, $J_{4,3}$ 4.4, $J_{4,5a}$ 3.0 Hz, 1H, H4), 3.76 (dd, 1H, H3), 3.61 (d, 1H, H1a), 3.61 (d, 1H, H1b), 2.76 (bs, 1H, O*H*), 1.22 (s, 9H, C*H_3*). ^{13}C-NMR (100 MHz, $CDCl_3$): δ_C 178.5 (*C*=O), 138.1 (Ar), 138.0 (Ar), 128.5-127.7 (Ar),

79.0 (C3), 78.2 (C4), 74.0 (CH_2Ph), 73.4 (CH_2Ph), 72.4 (CH_2Ph), 71.1 (C1), 70.8 (C2), 63.6 (C5), 38.9 ($C(CH_3)_3$), 27.3 ($C(CH_3)_3$). HRMS(ESI): calcd. for $C_{31}H_{38}O_6Na$ 529.2566; found 529.2507.

1,3,4-tri-*O*-benzyl-2,5-di-*O*-pivaloyl-D-ribitol (30)

R_f (pentane/ethyl acetate 8:1) 0.65. $[\alpha]_D^{293K}$ +6.5 (*c 1.0*, $CHCl_3$); ^{1}H-NMR (400 MHz, $CDCl_3$): δ_H 7.36-7.25 (m, 15H, Ar*H*), 5.44 (q, 1H, $J_{2,1} = J_{2,3}$ 4.9 Hz, H2), 4.73 (d, 1H, *J* 11.1 Hz, C*H*HPh), 4.68 (d, 1H, *J* 11.5 Hz, C*H*HPh), 4.62 (d, 1H, CH*H*Ph), 4.56 (dd, *J* 12.0 Hz, $J_{5a,4}$ 2.9 Hz, 1H, H5a), 4.54 (d, 1H, CH*H*Ph), 4.49 (d, 1H, *J* 12.0 Hz, C*H*HPh), 4.43 (d, 1H, CH*H*Ph), 4.19 (dd, 1H, $J_{5b,4}$ 5.4 Hz, H5b), 3.92 (dd, 1H, $J_{3,4}$ 5.6 Hz, H3), 3.81 (td, 1H, H4), 3.70-3.67 (m, 2H, H1), 1.22 (s, 9H, CH_3), 1.20 (s, 9H, CH_3). ^{13}C-NMR (100 MHz, $CDCl_3$): δ_C 178.4 (*C*=O), 177.5 (*C*=O), 138.3 (Ar), 138.1 (Ar), 128.5-127.7 (Ar), 78.1, 77.2, 74.0, 73.2, 72.3, 71.9, 68.6, 63.3, 38.9 ($C(CH_3)_3$), 38.9 ($C(CH_3)_3$), 27.4 (CH_3), 27.3 (CH_3). HRMS(ESI): calcd. for $C_{34}H_{46}O_7Na$ 613.3141; found 613.3148.

1,3,4-tri-*O*-benzyl-5-*O*-pivaloyl-2-*O*-triflyl-D-ribitol (13)

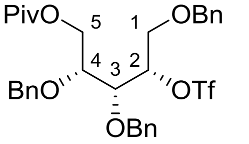

13

Alcohol **12** (1.63 g, 3.22 mmol) was dissolved in anhydrous dichloromethane (23 mL) and anhydrous pyridine (0.70 mL, 8.69 mmol, 2.7 equiv.) at 0 °C. After 10 minutes, triflic anhydride (0.70 mL, 4.16 mmol, 1.3 equiv.) was added and the reaction mixture stirred for 10 minutes at 0 °C before diluting with dichloromethane. The mixture was washed with cold 1M hydrochloric acid and sat. aq. $NaHCO_3$, dried over $MgSO_4$, filtered, and concentrated under reduced pressure to yield the product **13** (1.98 g). The crude product was used in the next step without further purification.

R_f (pentane/ ethyl acetate 8:1) 0.68. ^{1}H-NMR (400 MHz, $CDCl_3$): δ_H 7.33 – 7.07 (m, 15H; Ar*H*), 5.49 – 5.44 (m, 1H, H2), 4.75 (d, *J* 10.9 Hz, 1H, C*H*HPh), 4.68 (d, *J* 11.8 Hz, 1H, C*H*HPh), 4.68 (d, *J* 12.3 Hz, 1H), 4.52 (d, 1H, CH*H*Ph), 4.44 (s, 2H, C*H*$_2$Ph), 4.39 (d, 1H, CH*H*Ph), 4.04 (dd, *J* 12.2Hz, *J* 3.2 Hz, 1H), 3.98 (dd, *J* 8.3 Hz, *J* 1.9 Hz, 1H), 3.78 (dd, *J* 11.6 Hz, *J* 8.0 Hz, 1H), 3.58 (ddd, *J* 11.7 Hz, *J* 7.1 Hz, *J* 3.1 Hz, 2H), 1.20 (s, 9H, C*H*$_3$). ^{13}C-NMR (100 MHz, $CDCl_3$): δ_C 178.2 (*C*=O), 137.5 (Ar), 137.2 (Ar), 136.9 (Ar), 128.7-127.4 (CF_3, Ar), 89.2, 78.0, 75.8, 74.5, 73.4, 71.8, 67.6, 60.6, 27.3 (CH_3). ^{19}F-NMR (400 MHz, $CDCl_3$) δ_F -74.9.

1,3,4-tri-*O*-benzyl-2-cyano-2-deoxy-5-*O*-pivaloyl-D-arabinitol (15) and (4*R*,3*S*,*E*)-1,3,4-tri-*O*-benzyloxy-5-*O*-pivaloyl-pent-1-ene (14)

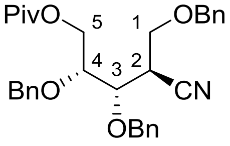

and

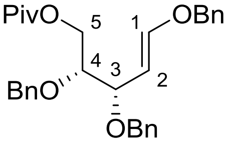

15 **14**

Crude triflate **13** (1.98 g) was dissolved in anhydrous THF (25 mL) and cooled on an ice bath before a solution of TBACN (1.58 g, 5.87 mmol, 1.9 equiv.) in anhydrous THF (12 mL) was added in a dropwise fashion under a

nitrogen atmosphere. After 1h and 15 minutes at 0 °C, the reaction was diluted with ethyl acetate, washed with sat. aq. $NaHCO_3$ and brine, dried over $MgSO_4$, filtered and concentrated under reduced pressure. The product was purified by flash column chromatography (pentane/ethyl acetate 15:1) to yield the product **15** (813 mg, 49 % over 2 steps) and the elimination by-product **14** (306 mg, 18 % over 2 steps).

Data for nitrile (**15)**:

R_f (pentane/ethyl acetate 15:1) 0.23; $[\alpha]_D^{293K}$ -0.4 (*c* 1.0, $CHCl_3$); ^{1}H-NMR (400 MHz, $CDCl_3$): δ_H 7.27-7.17 (m, 15H, Ar*H*), 4.76 (d, 1H, *J* 11.0 Hz, C*H*HPh), 4.72 (dd, 1H, $J_{5a,5b}$ 12.3 Hz, $J_{4,5}$ 2.9 Hz, H5a), 4.66 (d, 1H, *J* 11.3 Hz, C*H*HPh), 4.57 (d, 1H, CH*H*Ph), 4.56 (d, 1H, C*H*HPh), 4.51 (d, 1H, *J* 11.9 Hz, C*H*HPh), 4.45 (d, 1H, CH*H*Ph), 4.09 (dd, 1H, $J_{4,5b}$ 3.6 Hz, 1H, H5b), 3.91 (dd, 1H, $J_{3,4}$ 7.9 Hz, $J_{2,3}$ 2.9 Hz, H3), 3.85 – 3.79 (m, 1H, H4), 3.71 – 3.66 (m, 2H, H1), 3.46 – 3.37 (m, 1H, H2), 1.23 (s, 9H, C*H*$_3$). ^{13}C NMR (100 MHz, $CDCl_3$): δ_C 178.1 (*C*=O), 137.6 (Ar), 137.3 (Ar), 137.2 (Ar), 128.6-127.9 (Ar), 118.1 (*C*N), 77.8, 74.9, 74.7, 73.3, 72.7, 66.7, 61.3, 39.0 (*C*(CH_3)$_3$), 35.6 (C2), 27.4 (*C*H$_3$). HRMS(ESI): calcd. for $C_{32}H_{37}NO_5Na$ 538.2569; found 538.2568.

Data for alkene (**14**):

R_f (pentane/ethyl acetate 12:1) 0.80; $[\alpha]_D^{293K}$ +18.6 (*c 1.0*, $CHCl_3$); ^{1}H-NMR (400 MHz, $CDCl_3$): δ_H 7.48 – 7.22 (m, 15H, Ar*H*), 6.51 (d, $J_{1,2}$ 12.8 Hz, 1H, H1), 4.89 (dd, $J_{2,3}$ 8.9 Hz, 1H, H2), 4.79 (s, 2H, C*H*$_2$Ph), 4.68 (s, 2H, C*H*$_2$Ph), 4.60 (d, *J* 12.0 Hz, 1H, C*H*HPh), 4.33 (d, 1H, CH*H*Ph), 4.28 (dd, $J_{5a,5b}$ 11.7, $J_{5a,4}$ 3.9 Hz, 1H, H5a), 4.13 (dd, $J_{5b,4}$ 6.2 Hz, 1H, H5b), 3.82 – 3.73 (m, 2H, H3, H4), 1.17 (s, 9H). ^{13}C-NMR (100 MHz, $CDCl_3$): δ_C 178.4 (*C*=O), 150.8 (C1), 138.6 (Ar), 138.5 (Ar), 136.7 (Ar), 128.7-127.6 (Ar), 101.1 (C2), 79.7, 77.5 (C3, C4), 73.3 (*C*H$_2$Ph), 71.3 (CH$_2$Ph), 69.5 (*C*H$_2$Ph), 64.3 (C5), 38.9 (*C*(CH_3)$_3$), 27.3 (*C*H$_3$). HRMS(ESI): calcd. for $C_{31}H_{36}O_5Na$ 511.2455; found 511.2461

2,3,5-*O*-benzyl-4-(((*tert*-butoxycarbonyl)amino)methyl)-4-deoxy-D-arabinitol (16)

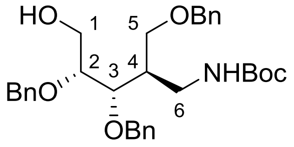

16

Nitrile **15** (636 mg, 1.23 mmol) was dissolved in anhydrous methanol (12.5 mL) and cooled to 0 °C before $NiCl_2{\cdot}6H_2O$ (1.17 g, 4.93 mmol, 4 equiv.) and Boc_2O (462 mg, 2.12 mmol, 1.7 equiv.) were added under a nitrogen atmosphere, giving a green solution. $NaBH_4$ (1.17 g, 30.8 mmol, 25 eq) was then added slowly, turning the solution black. The mixture was allowed to heat to room temperature and stirred overnight. The next morning the mixture was concentrated under reduced pressure, taken up in ethyl acetate, filtered through Celite® and concentrated. The crude mixture was then dissolved in anhydrous methanol (6.2 mL) and K_2CO_3 (401 mg, 2.90 mmol, 2.4 equiv.) and Boc_2O (266 mg, 1.22 mmol, 1 equiv.) were added before the mixture was heated to 40 °C. The reaction was left for 44 hours at 40 °C before being cooled to room temperature, diluted with diethyl ether, washed with brine, dried over $MgSO_4$, filtered and concentrated. The crude product was purified by flash column chromatography (pentane/ethyl acetate 3:1) to give the de-pivaloylated product **16** (334 mg, 51 % over 2 steps).

R_f (pentane/ethyl acetate 4:1) 0.17; ^{1}H-NMR (400 MHz, $CDCl_3$): δ_H 7.39-7.20 (m, 15H, Ar*H*), 5.11 (bs, 1H, N*H*), 4.69 (d, 1H, *J* 11.1 Hz, C*H*HPh), 4.65 (d, 1H, *J* 11.6 Hz, C*H*HPh), 4.59 (d, 1H, CH*H*Ph), 4.55 (d, 1H, CH*H*Ph), 4.47 (d, 1H, *J* 11.9 Hz, C*H*HPh), 4.38 (d, 1H, CH*H*Ph), 3.86 (dd, 1H, *J* 6.2 Hz, *J* 4.2 Hz, H3), 3.80 (dd, 1H, $J_{1a,1b}$ 12.0 Hz, $J_{1a,2}$ 4.0 Hz, H1a), 3.73 (dd, 1H, $J_{1b,2}$ 3.9 Hz, H1b), 3.65-3.59 (m, 1H, H2), 3.48 (d, 2H, $J_{5,4}$ 6.0 Hz, H5), 3.39 – 3.31 (m, 1H, H6a), 3.26-3.17 (m, 1H, H6b), 2.18-2.13 (m, 1H, H4), 1.39 (s, 9H, C*H*$_3$). ^{13}C-NMR (100 MHz, $CDCl_3$) δ_C 156.2 (*C*=O), 138.2 (Ar), 138.1 (Ar), 138.0 (Ar), 128.6 (Ar), 128.5 (Ar), 128.5 (Ar), 128.1 (Ar), 128.0 (Ar), 128.0 (Ar), 127.9 (Ar), 127.8 (Ar), 79.6 (C2), 78.9 (*C*(CH_3)$_3$), 78.2 (C3), 74.4 (*C*H_2Ph), 73.4 (*C*H_2Ph), 72.1 (*C*H_2Ph), 70.5 (C5), 60.8 (C1), 40.3 (C4), 39.7 (C6), 28.5 (*C*H_3). HRMS(ESI): calcd. for $C_{32}H_{41}NO_6H$ 536.3012; found 536.3009.

(3*R*,4*S*,5*R*)-3,4-di-*O*-benzyl-5-(benzyloxymethyl)piperidine (17)

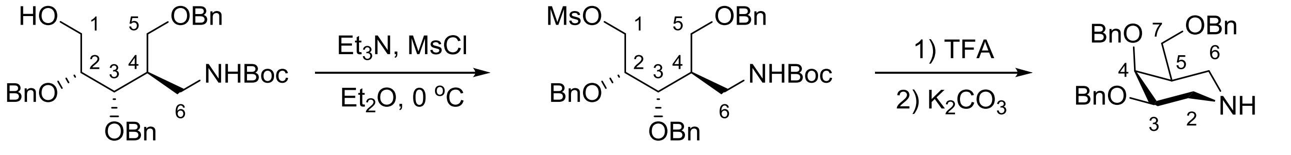

16 **27** **17**

2,3,5-tri-*O*-benzyl-4-(((*tert*-butoxycarbonyl)amino)methyl)-4-deoxy-1-*O*-mesyl-D-arabinitol (27)

The alcohol **16** (75 mg 140 µmol) was dissolved in anhydrous diethyl ether (2.3 mL) and cooled to 0 °C. Triethyl amine (40 µL, 287 µmol, 2.1 eq) was added followed by slow addition of mesyl chloride (20 µL, 257 µmol, 1.8 eq) under a nitrogen atmosphere. After 25 min the reaction was quenched with water and diluted with ethyl acetate. The organic phase was washed with water and brine, dried over $MgSO_4$, filtered and concentrated under reduced pressure to give the crude product **27** (84 mg) which was used directly in the next step without further purification.

R_f (pentane/ethyl acetate 3:2) 0.71; ^{1}H-NMR (400 MHz, $CDCl_3$) δ_H 7.38 – 7.23 (m, 15H, Ar*H*), 5.01 (s, 1H, H1), 4.67 (d, *J* 11.3 Hz, 1H, C*H*HPh), 4.66 (d, 1H, CH*H*Ph), 4.60 - 4.50 (m, 3H), 4.47 (d, *J* 11.8 Hz, 1H, C*H*HPh), 4.41 (d, 1H, CH*H*Ph), 4.36 (dd, *J* 11.2 Hz, *J* 4.5 Hz, 1H), 3.85 (d, *J* 3.5 Hz, 1H), 3.52 (t, *J* 5.4 Hz, 1H), 3.40 – 3.31 (m, 1H), 3.27 – 3.17 (m, 1H), 2.91 (s, 3H), 2.16 (bs, 1H, C*H*$_3SO_3$-), 1.69 (bs, 1H), 1.42 (s, 9H, C*H*$_3$). ^{13}C-NMR (100 MHz, $CDCl_3$) δ_c 156.1 (*C*=O), 138.0 (Ar), 137.9 (Ar), 137.6 (Ar), 128.6 (Ar), 128.6 (Ar), 128.6 (Ar), 128.3 (Ar), 128.2 (Ar), 128.0 (Ar), 128.0 (Ar), 127.9 (Ar), 79.1, 78.1, 77.8, 77.8, 74.5, 73.4, 72.8, 70.1, 69.0, 40.6, 39.8, 37.6, 29.8, 28.6 (CH_3). HRMS(ESI): calcd. for $C_{33}H_{43}NO_8Na$ 636.2602 found 636.2609

(3R,4S,5R)-3,4-di-*O*-benzyl-5-(benzyloxymethyl)piperidine (17)

The mesylate **27** (140 µmol) was mixed with trifluoroacetic acid (1.2 mL) under nitrogen atmosphere. After 10 min the mixture was concentrated under reduced pressure, redissolved in dichloromethane and sat. aq. $NaHCO_3$. The mixture was stirred until no more gas was evolved and the aqueous phase was then extracted with dichloromethane. The combined organic phases were dried over $MgSO_4$ and filtered. The extracts were added K_2CO_3 and heated to reflux for 4h to induce ring closing. The mixture was then cooled to room temperature, filtered and concentrated under reduced pressure. The crude product was purified by flash column chromatography (EtOAc/Et_3N/MeOH 80:4:1) to give the product **17** (37 mg, 63 % over 3 steps)

R$_f$ (MeOH/Et_3N/ethyl acetate 1:2:40) 0.22; ^{1}H-NMR (400 MHz, $CDCl_3$): δ_H 7.44-6.97 (m, 15H, Ar*H*), 4.84 (d, *J* 11.5 Hz, 1H, C*H*HPh), 4.57 (d, *J* 12.1 Hz, 1H, C*H*HPh), 4.52 (d, 1H, CH*H*Ph), 4.49 (d, 1H, CH*H*Ph), 4.37 (s, 2H,

CH_2Ar), 3.98 (s, 1H, H4), 3.50-3.28 (m, 3H, *H*3, H6/H7), 2.97 (dd, *J* 9.7 Hz, *J* 11.9 Hz, 1H, H2a), 2.87 (d, *J* 8.9 Hz, 1H, H2b), 2.72-2.54 (m, 2H, H6/H7), 1.90 (bs, 1H), 1.78 (bs, 1H), 1.19 (s, 1H, H5). ^{13}C-NMR (100 MHz, $CDCl_3$): δ_C 139.5 (Ar), 138.9 (Ar), 138.5 (Ar), 128.5 (Ar), 128.5 (Ar), 128.3 (Ar), 127.9 (Ar), 127.8 (Ar), 127.7 (Ar), 127.6 (Ar), 127.4 (Ar), 127.4 (Ar), 79.1 (C3), 74.9 (C4), 73.7 (*C*H_2Ph), 73.3 (*C*H_2Ph), 71.1 (*C*H_2Ph), 69.2 (C7/C6), 45.8 (C2), 43.8 (C7/C6), 42.4 (C5). HRMS(ESI): calcd. for $C_{27}H_{31}NO_3H$ 418.2377 found 418.2387

Isogalactofagomine(1)

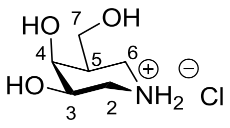

1

The benzyl protected compound **17** (13 mg, 31 µmol) was dissolved in methanol/ethyl acetate 1:1 (1 mL) and added a catalytic amount of Pearlman's catalyst (20 %). The reaction flask was flushed with hydrogen gas three times and the reaction stirred under hydrogen atmosphere. After 30 min, two drops of concentrated aq. HCl were added and the reaction left overnight. The next day the reaction was filtered through a pad of Celite® and concentrated under reduced pressure. The crude product was taken up in water and washed with diethyl ether. Purified by flash column chromatography and ion exchange chromatography (Amberlite IR 120 H^+) and then added 1 M aq. HCl and concentrated to give the product **1** (3.7 mg, 65%)

R_f (ethanol/NH_4OH (25%) 4:1) 0.31; $[\alpha]_D^{293K}$ +5.1 (c 1.0, EtOH); ^{1}H-NMR (400 MHz, D_2O): δ_H 4.15 (bs, 1H, H4), 3.97 (ddd, $J_{3,2ax}$ 11.5 Hz, $J_{3,2eq}$ 4.8 Hz, $J_{3,4}$ 2.6 Hz, 1H, H3), 3.73 (dd, $J_{7a,7b}$ 11.3 Hz, $J_{7b,5}$ 6.7 Hz, 1H, H7b), 3.63 (dd, $J_{7a,5}$ 7.2 Hz, 1H, H7a), 3.27 (dd, $J_{2eq,2ax}$ 15.6 Hz, 1H, $H2_{eq}$), 3.31 (dd, $J_{6ax,6eq}$ 12.2 Hz, $J_{5,6eq}$ 4.2 Hz, 1H, $H6_{eq}$) 3.09 (t, $J_{6ax,5}$ 11.8Hz, 1H, $H6_{ax}$), 2.95 (t, 1H, $H2_{ax}$), 2.20 – 2.09 (m, 1H, H5). ^{13}C-NMR (100 MHz, D_2O): δ_C 66.0, 65.7, 60.0, 42.0, 39.7, 39.1. HRMS(ESI): calcd. for $C_6H_{13}NO_3H$ 148.0968 found 148.1009

NMR data is in accordance with previously reported values[2]

(3*R*,4*S*,5*R*)-1-(*tert*-butoxycarbonyl)- 5-(benzyloxymethyl)-3,4-di-*O*-benzyl-piperidin-2-one (18)

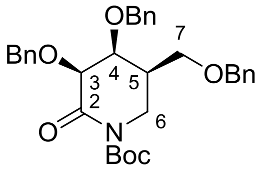

18

The alcohol **16** (334 mg, 624 µmol), TEMPO (66 mg, 424 µmol, 0.62 eq) and tetra-butylammonium bromide (66 mg, 206 µmol, 0.33 eq) was dissolved in dichloromethane (34 mL) and added *m*-CPBA (245 mg, 1.42 mmol, 2.3 eq). The reaction was completed immediately after the addition and was then washed with sat. aq. $NaHCO_3$, the organic phase dried over $MgSO_4$, filtered and concentrated under reduced pressure. The crude product was purified by flash column chromatography (pentane/ethyl acetate 8:1) to give the lactam **18** as a white solid (244 mg, 74 %)

R_f (pentane/ethyl acetate 8:1) 0.28; $[\alpha]_D^{293K}$ - 69.2 (c 1.0, $CHCl_3$); Mp 93.1-94.8°C; ^{1}H-NMR (400 MHz, $CDCl_3$): δ_H 7.49 – 7.16 (m, 15H, ArH), 5.18 (d, *J* 12.0 Hz, 1H, C*H*HPh), 4.98 (d, *J* 11.6 Hz, 1H, C*H*HPh), 4.65 (d, 1H, CH*H*Ph), 4.56 (d, 1H, CH*H*Ph), 4.38 (s, 2H, CH_2Ph), 4.14 (bs, 1H, H4), 3.98 (d, $J_{3,4}$ 2.3 Hz, 1H, H3), 3.59 (dd, *J* 12.2 Hz, *J* 5.9 Hz, 1H, H6b), 3.53 – 3.38 (m, 2H, H6a, H7a), 3.34 (dd, *J* 8.9 Hz, *J* 6.1 Hz, 1H, H7b), 2.30 (m, 1H, H5), 1.51 (s, 9H, CH_3). ^{13}C-NMR (100 MHz, $CDCl_3$): δ_C 171.3 (*C*=O), 152.8 (Ar), 138.6 (Ar), 138.3 (Ar), 137.9 (Ar), 128.6 (Ar), 128.5 (Ar), 128.3 (Ar), 128.2 (Ar), 127.9 (Ar), 127.9 (Ar), 127.8 (Ar), 127.8 (Ar), 127.7 (Ar), 83.1 (*C*(CH_3)$_3$), 81.2 (C3), 75.2 (C4), 74.3 (*C*H_2Ph), 73.9 (*C*H_2Ph), 73.5 (*C*H_2Ph), 68.5 (C7), 45.0 (C6), 38.1 (C5), 28.1 (*C*H_3). HRMS(ESI): calcd. for $C_{32}H_{37}NO_6Na$ 554.2513 found 554.2514.

Isogalactofagomine lactam (**3**)

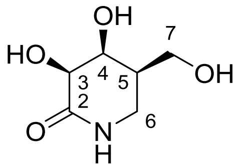

3

The protected lactam **18** (150 mg, 282 µmol) was dissolved in ethyl acetate/methanol 1:1 (8.6 mL) and added Pearlman's catalyst (20 %) (39.5 mg, 56 µmol, 20 %). The flask was flushed with hydrogen gas and the reaction stirred under hydrogen atmosphere for 1h 30 min. The mixture was then filtered through Celite®, concentrated under reduced pressure, redissolved in trifluoroacetic acid (1.9 mL) and stirred at room temperature for 15 min before being concentrated again. The crude mixture was purified by flash column chromatography (NH_4OH (25 %)/ethanol 1:3) to give the product **3** as a slightly yellow solid (30.7 mg, 68 % over 2 steps)

R_f (NH_4OH (25 %)/ethanol 2:1) 0.53; $[\alpha]_D^{293K}$ +8.6 (c 1.0, H_2O); ^{1}H-NMR (400 MHz, D_2O) δ_H 4.27 (dd, $J_{3,4}$ 3.1 Hz, $J_{4,5}$ 1.4 Hz, 1H, H4), 4.23 (d, 1H, H3), 3.75 (dd, $J_{7a,7b}$ 11.1 Hz, $J_{5,7a}$ 7.4 Hz, 1H, H7a), 3.62 (dd, $J_{5,7b}$ 6.8 Hz, 1H, H7b), 3.36 (dd, $J_{6eq,6az}$ 12.4 Hz, $J_{6eq,5}$ 6.5 Hz, 1H, H6eq), 3.14 (t, $J_{6eq,6az} = J_{6ax,5}$ 12.0 Hz, 1H, H6ax), 2.41 – 2.28 (m, 1H, H5). ^{13}C-NMR (100 MHz, D_2O) δ_C 173.8 (C2), 69.7 (C3/C4), 67.2 (C3/C4), 60.5 (C7), 39.6 (C5/C6), 38.0 (C5/C6). HRMS(ESI): calcd. for $C_6H_{11}NO_4Na$ 184.0586 found 184.0578.

NMR data were in accordance with literature values[3]

2,3,5-tri-*O*-benzyl-1,4-di-*O*-mesyl-D-ribitol (19)

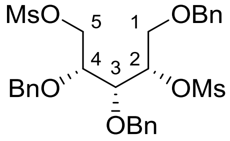

19

The diol **11** (112 mg, 265 µmol) was dissolved in anhydrous dichloromethane (4.4 mL) and anhydrous triethylamine (0.22 mL, 1.58 mmol, 6 eq) and a catalytic amount of 4-(*N,N*-dimethylamino)pyridine were added under a nitrogen atmosphere. Methanesulfonyl chloride (60 µL, 772 µmol, 2.9 eq) was slowly added at room temperature and after stirring for 20 min, the reaction was diluted with dichloromethane, washed

with sat. aq. NH_4Cl and brine, dried over $MgSO_4$, filtered and concentrated under reduced pressure to give the crude product **19** (155 mg).

*R*f (pentane/ethyl acetate 3:2) 0.67; $[\alpha]_D^{293K}$ – 3.4 (c 1.0, $CHCl_3$); ^{1}H-NMR (400 MHz, $CDCl_3$): δ_H 7.38 - 7.24 (m, 15H, Ar*H*), 5.14 (dt, $J_{4,5a}$ 7.7 Hz, $J_{4,3}$ = $J_{4,5b}$ 3.1 Hz, 1H, H4), 4.76 (d, *J* 11.0 Hz, 1H, C*H*HPh), 4.71 (d, *J* 11.4 Hz, 1H, C*H*HPh), 4.60 (d, 1H, CH*H*Ph), 4.55 (dd, $J_{1a,1b}$ 11.4 Hz, $J_{1a,2}$ 2.7 Hz, 1H, H1a), 4.50 (d, 1H, CH*H*Ph), 4.45 (s, 2H, C*H*HPh), 4.33 (dd, $J_{1a,1b}$ 11.4 Hz, $J_{1b,2}$ 4.1 Hz, 1H, H1b), 3.92 (dd, $J_{3,2}$ 7.3 Hz, $J_{3,4}$ 2.9 Hz, 1H, H3), 3.77 (d, $J_{2,3}$ 6.9 Hz, 1H, H2), 3.74 (dd, $J_{5a,5b}$ 11.0 Hz, $J_{4,5a}$ 7.8 Hz, 1H, H5a), 3.60 (dd, $J_{5a,5b}$ 11.0 Hz, $J_{4,5b}$ 3.3 Hz, 1H, H5b), 3.01 (s, 3H, C$H_3$$SO_3$), 2.95 (s, 3H, C$H_3$$SO_3$). ^{13}C-NMR (100 MHz, $CDCl_3$): δ 137.4 (Ar), 137.1 (Ar), 136.9 (Ar), 128.7 (Ar), 128.6 (Ar), 128.6 (Ar), 128.5 (Ar), 128.4 (Ar), 128.3 (Ar), 128.1 (Ar), 127.9 (Ar), 81.6 (C4), 77.5 (C3), 76.1 (C2), 74.1 (*C*H_2Ph), 73.5 (*C*H_2Ph), 72.5 (*C*H_2Ph), 68.8 (C5), 67.7 (C1), 38.7 (*C*H_3SO_3), 37.7 (*C*H_3SO_3). HRMS(ESI): calcd. for $C_{28}H_{34}O_9S_2Na$ 601.1536 found 601.1538

(2*R*,3*S*,4*R*)-1-*N*-benzyl-3,4-di-*O*-benzyl-2-((benzyloxy)methyl)pyrrolidine (20)

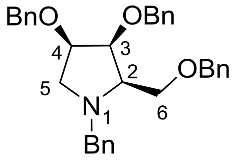

20

The crude dimesylate **19** (155 mg) was mixed with benzylamine (1.3 mL) under a nitrogen atmosphere. The mixture was stirred at 70 °C overnight and then cooled to room temperature, diluted with ethyl acetate and washed with water and brine. The organic phase was then dried over $MgSO_4$, filtered and concentrated under reduced pressure. Excess benzylamine was removed by co-evaporation with *p*-xylene. The crude compound was purified by flash column chromatography (pentane/ethyl acetate 10:1) to give the product **20** (99.3 mg, 93 % over 2 steps)

*R*f (pentane/ethyl acetate 10:1) 0.38; $[\alpha]_D^{293K}$ – 33.0 (c 1.0, $CHCl_3$); ^{1}H-NMR (400 MHz, $CDCl_3$): δ_H 7.50 – 7.15 (m, 15H, Ar*H*), 4.73 (d, *J* 12.0 Hz, 1H, C*H*HPh), 4.60 (d, *J* 12.1 Hz, 1H, C*H*HPh), 4.51 (s, 2H, CH_2Ph), 4.49 (s, 2H, CH_2Ph), 4.06 (t, $J_{3,4}$ = $J_{3,2}$ 6.0 Hz, 1H, H3), 4.04 (d, *J* 13.9 Hz, 1H, NC*H*HPh), 3.94 (dt, $J_{4,5b}$ 5.9 Hz, $J_{4,5a}$ 4.9 Hz, 1H, H4), 3.90 (dd, $J_{6a,6b}$ 9.4 Hz, $J_{6a,2}$ 6.0 Hz, 1H, H6a), 3.67 (dd, $J_{6b,2}$ 6.0 Hz, 1H, H6b), 3.59 (d, 1H, NCH*H*Ph), 3.17 (q, 1H, H2), 3.07 (dd, $J_{5a,5b}$ 10.6 Hz, 1H, H5a), 2.56 (dd, 1H, H5b). ^{13}C-NMR (100 MHz, $CDCl_3$): δ 139.4 (Ar), 138.9 (Ar), 138.8 (Ar), 138.7 (Ar), 128.9 (Ar), 128.8 (Ar), 128.4 (Ar), 128.4 (Ar), 128.3 (Ar), 128.2 (Ar), 127.9 (Ar), 127.8 (Ar), 127.7 (Ar), 127.6 (Ar), 127.5 (Ar), 127.5 (Ar), 126.9 (Ar), 79.0 (C3), 77.5 (C4), 73.5 (*C*H_2Ph), 73.0 (*C*H_2Ph), 71.7 (*C*H_2Ph), 70.7 (C6), 64.5 (C2), 59.9 (N*C*H_2Ph), 55.0 (C5). HRMS(ESI): calcd. for $C_{33}H_{35}NO_3H$ 494.2690 found 494.2692.

1,4-dideoxy-1,4-imino-D-lyxitol (4)

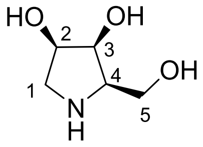

4

The benzyl protected pyrrolidine **20** (99.3 mg, 246 µmol) was dissolved in a methanol/ethyl acetate 1:1 (8 mL) and added 2 drops of concentrated aq. HCl. Pearlman's catalyst (20 %) (15.2 mg, 22 µmol, 9%) was added and the flask evacuated 3 times with hydrogen. The reaction was stirred overnight, added more catalyst (11.9 mg) and stirred overnight again. The next day more catalyst was added (12.3 mg) and after 5 more hours the reaction was filtered through a pad of Celite® and concentrated. The crude compound was purified by flash column chromatography (dichloromethane/ethanol/methanol/NH_4OH (25 %) 5:2:2:1) to give the product **4** (18.3 mg, 44 %)

R_f (methanol/NH_4OH (25 %) 9:1) 0.27; $[\alpha]_D^{293K}$ +29.6 (c 1.0, H_2O) Litt.: +20.8 (Dangerfield 2008); ^{1}H-NMR (400 MHz, D_2O): δ_H 4.48 (td, $J_{2,1a} = J_{2,1b}$ 7.4 Hz, $J_{4,2}$ 4.1 Hz, 1H, H2), 4.33 (t, $J_{3,4}$ 4.1 Hz, 1H, H3), 3.98 (dd, $J_{5a,5b}$ 12.2 Hz, $J_{5a,4}$ 4.8 Hz, 1H H5a), 3.88 (dd, $J_{5b,4}$ 8.5 Hz, 1H, H5b), 3.73 (dt, 1H, H4), 3.52 (dd, $J_{1a,1b}$ 12.1 Hz, 1H, H1a), 3.20 (dd, 1H, H1b). ^{13}C-NMR (100 MHz, D_2O): δ_C 69.9 (C2), 69.7 (C3), 62.4 (C4), 57.6 (C5), 47.0 (C1). HRMS(ESI): calcd. for $C_5H_{11}NO_3H$ 134.0812 found 134.0812

NMR data were in accordance with literature values[4]

2,3,5-tri-*O*-benzyl-L-ribono-1,4-lactone (21)

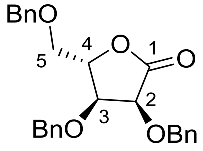

21

The diol **11** (117 mg, 276 µmol) was dissolved in anhydrous dichloromethane (1.5 mL) and iodobenzene diacetate (283 mg, 879 µmol, 3.2 eq) and TEMPO (10.0 mg, 64 µmol, 23 %) were added to give a weak orange solution. After 4 h 10 min the reaction was quenched by adding 20 % aq. $Na_2S_2O_3$ and diluted with diethyl ether. The organic phase was washed with sat. aq. $NaHCO_3$ and brine, dried over $MgSO_4$, filtered and concentrated under reduced pressure. Purified by flash column chromatography (pentane/ethyl acetate 4:1) to give the product lactone **21** (81 mg, 70%) as a white solid.

R_f (pentane/ethyl acetate 4:1) 0.36; $[\alpha]_D^{293K}$ – 62.0 (c 1.0, $CHCl_3$); ^{1}H-NMR (400 MHz, $CDCl_3$): δ_H 7.41 – 7.22 (m, 13H, Ar*H*), 7.19 – 7.13 (m, 2H, Ar*H*), 4.95 (d, *J* 12.0 Hz, 1H, C*H*HPh), 4.74 (d, 1H, CH*H*Ph), 4.70 (d, *J* 11.9 Hz, 1H, C*H*HPh), 4.54 (d, 1H, CH*H*Ph), 4.54 (q, $J_{4,5a} = J_{4,5b} = J_{4,3}$ 2.4 Hz, 1H, H4), 4.49 (d, *J* 11.9 Hz, 1H, C*H*HPh), 4.41 (d, 11.9 Hz, 1H, CH*H*Ph), 4.39 (d, $J_{2,3}$ 5.7 Hz, 1H, H2), 4.09 (dd, 1H, H3), 3.66 (dd, $J_{5a,5b}$ 11.0 Hz, 1H, H5a), 3.55 (dd, H5b). ^{13}C-NMR (100 MHz, $CDCl_3$): δ_C 173.8 (C1), 137.3 (Ar), 137.19 (Ar), 137.0 (Ar), 128.6 (Ar), 128.6 (Ar), 128.6 (Ar), 128.3 (Ar), 128.2 (Ar), 128.1 (Ar), 128.1 (Ar), 127.7 (Ar), 81.9 (C4), 75.4 (C3), 73.8 (C2), 73.7 (CH_2Ph), 72.8 (CH_2Ph), 72.5 (CH_2Ph), 68.8(C5). HRMS(ESI): calcd. for $C_{26}H_{26}O_5Na$ 441.1672 found 441.1670

NMR data were in accordance with its antipode[5]

2,3,5-tri-*O*-benzyl-L-ribofuranose (**22**)

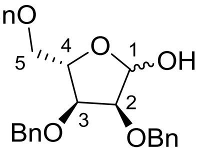

22

The lactone **21** (88.7 mg, 212 µmol) was dissolved in anhydrous dichloromethane (0.6 mL) and cooled to -78°C. DIBAL 1M in toluene (0.23 mL, 230 µmol, 1.1 eq) was added under a nitrogen atmosphere and the mixture stirred at-78°C for 1h 45 min. More DIBAL (0.1 mL) was then added and after an extra 15 min the reaction was quenched with water, extracted with dichloromethane, dried over $MgSO_4$, filtered and concentrated under reduced pressure. This gave the crude product **22** (80 mg) as a 1:1.6 anomeric mixture, which could be used in the next step without further purification.

R_f (pentane/ethyl acetate 3:1) 0.22; ^{1}H-NMR (400 MHz, $CDCl_3$) δ_H 7.41 – 7.20 (m, 54.2H, Ar*H*), 5.33 (d, *J* 3.9 Hz, 1H, H1 minor), 5.30 (d, *J* 4.1 Hz, 1.9H, H1 major), 4.75 – 4.55 (m, 13.5H), 4.53 (d, *J* 12.7 Hz, 3.2H), 4.46 (d, *J* 12.4 Hz, 3.2H), 4.41 – 4.35 (m, 3.4H), 4.31 (dt, *J* 6.0 Hz, *J* 2.8 Hz, 1.1H), 4.23 (dd, *J* 6.7 Hz, *J* 4.7 Hz, 1.2H), 4.19 (d, *J* 11.3 Hz, 1.9H), 4.02 – 3.95 (m, 4.2H), 3.86 (d, *J* 4.6 Hz, 1.2H), 3.67 (dd, *J* 10.4 Hz, *J* 2.8 Hz, 1.2H), 3.52 – 3.42 (m, 5.5H), 3.29 (d, *J* 7.0 Hz, 1.2H). Major isomer: ^{13}C-NMR (100 MHz, $CDCl_3$) δ_C 138.0 (Ar), 137.6 (Ar), 137.6 (Ar), 128.6 (Ar), 128.6 (Ar), 128.5 (Ar), 128.1 (Ar), 128.0 (Ar), 127.7 (Ar), 96.4 (C1), 81.2, 77.9, 77.8, 73.6, 73.0, 72.6, 70.1. Minor isomer: ^{13}C-NMR (100 MHz, $CDCl_3$) δ_C 137.9 (Ar), 137.8 (Ar), 137.4 (Ar), 128.6 (Ar), 128.2 (Ar), 128.1 (Ar), 128.0 (Ar), 127.9 (Ar), 127.9 (Ar), 100.5 (C1), 81.0, 80.9, 77.4, 77.3, 72.6, 72.4, 69.5. HRMS(ESI): calcd. for $C_{26}H_{28}O_5Na$ 443.1829 found 443.1830

1-(2-*tert*-butyloxycarbonyl)hydrazino-1-deoxy-2,3,5-tri-*O*-benzyl-L-ribitol (**23**)

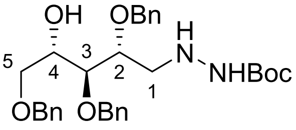

23

The crude lactol **22** (80 mg, 190 µmol) was dissolved in anhydrous methanol (1 mL) and added *tert*-butylcarbazate (55 mg, 418 µmol, 2.2 eq), $NaCNBH_4$ (46 mg, 734 µmol, 3.9 eq) and glacial acetic acid (20 µL, 347 µmol, 1.8 eq). The mixture was heated to 60 °C, stirred overnight and then cooled to room temperature and added sat. aq. $NaHCO_3$ and water. The aqueous phase was extracted with dichloromethane and the combined organic phases dried over $MgSO_4$, filtered and concentrated under reduced pressure. The crude product was purified by flash column chromatography (pentane/ethyl acetate 2:1) to give the product **23** (72.8 mg, 64 %)

R_f (pentane/ethyl acetate 2:1) 0.31; $[\alpha]_D^{293K}$ – 9.6 (c 1.0, $CHCl_3$); ^{1}H-NMR (400 MHz, $CDCl_3$) δ_H 7.32 – 7.15 (m, 15H, Ar*H*), 6.19 (bs, 1H,N*H*), 4.69 (d, *J* 11.4 Hz, 1H, C*H*HPh), 4.58 (d, *J* 11.7 Hz, 1H, C*H*HPh), 4.54 (d, 1H, CH*H*Ph), 4.51 (d, 1H, CH*H*Ph), 4.48 (d, *J* 11.9 Hz, 1H, C*H*HPh), 4.44 (d, 1H, CH*H*Ph), 3.92 – 3.86 (m, 1H, H4), 3.82 (dd,

$J_{5,6}$ 8.2 Hz, $J_{6,7b}$ 5.0 Hz, 1H, H4), 3.74 (dd, $J_{4,5}$ 3.1 Hz, 1H, H3), 3.58 (dd, $J_{3a,3b}$ 9.7 Hz, $J_{3a,4}$ 3.0 Hz, 1H, H1a), 3.51 (dd, $J_{3b,4}$ 5.8 Hz, 1H, H1b), 3.25 – 3.16 (m, 1H, H5a), 3.06 (dd, $J_{7a,7b}$ 12.7 Hz, 1H, H5b), 1.39 (s, 6H, C*H*$_3$). ^{13}C-NMR (100 MHz, $CDCl_3$) δ_C 156.7 (*C*=O), 138.5 (Ar), 138.3 (Ar), 138.1 (Ar), 128.6 (Ar), 128.5 (Ar), 128.4 (Ar), 128.1 (Ar), 128.0 (Ar), 128.0 (Ar), 127.9 (Ar), 127.8 (Ar), 127.8 (Ar), 127.7 (Ar), 80.5 (*C*(CH_3)$_3$), 79.4 (C4), 77.8 (C3), 73.8 (*C*H$_2$Ph), 73.5 (*C*H$_2$Ph), 71.8 (*C*H$_2$Ph), 71.6 (C1), 70.4 (C2), 51.0 (C5), 28.4 (*C*H$_3$). HRMS(ESI): calcd. for $C_{31}H_{46}N_2O_6H$ 537.2959 found 537.2968.

1-(1-Acetyl-2-*tert*-butyloxycarbonyl)hydrazino-1-deoxy-2,3,5-tri-*O*-benzyl-4-methanesulfonyl-L-ribitol (**24**)

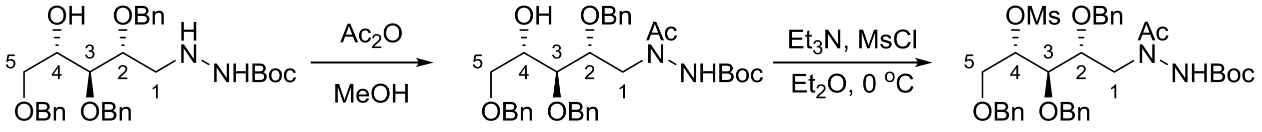

23 **28** **24**

1-(1-Acetyl-2-*tert*-butyloxycarbonyl)hydrazino-1-deoxy-2,3,5-tri-*O*-benzyl-L-ribitol (**28**)

The hydrazine **23** (620 mg, 1.15 mmol) was mixed with acetic anhydride (6 mL, 63.5 mmol, 55 eq) and methanol (60 mL) and stirred for 2 h at room temperature. The reaction was then quenched with sat. aq. $NaHCO_3$ and stirred for 1h. The methanol was removed under reduced pressure and the remaining aqueous phase was extracted with dichloromethane. The combined organic phases were washed with brine, dried over $MgSO_4$, filtered and concentrated under reduced pressure to give the crude product **28** (644 mg). The product was co-polar with the starting material.

R_f (dichloromethane/ethyl acetate 3:1) 0.27; ^{1}H-NMR (400 MHz, $CDCl_3$) δ_H 7.51 – 7.19 (m, 15H), 4.83 – 4.61 (m, 3H), 4.59 – 4.44 (m, 3H), 4.09 (bs, 1H), 3.92 (bs, 1H), 3.76 (dd, *J* 8.1 Hz, *J* 2.5 Hz, 1H), 3.70 – 3.55 (m, 2H), 2.99 (bs, 1H), 1.92 (bs, 3H), 1.44 (s, 9H). ^{13}C-NMR (100 MHz, $CDCl_3$) δ 154.4 (*C*=O), 138.2 (Ar), 138.0 (Ar), 128.7 (Ar), 128.6 (Ar), 128.6 (Ar), 128.5 (Ar), 128.5 (Ar), 128.3 (Ar), 128.1 (Ar), 128.0 (Ar), 127.9 (Ar), 127.8 (Ar), 81.8 (broad), 78.8 (broad), 74.0 (broad), 73.5, 72.0 (broad), 71.2, 70.3 (broad), 28.3 (*C*H$_3$), 20.6. HRMS(ESI): calcd. for $C_{33}H_{42}N_2O_7H$ 579.3065 found 579.3071

NMR signals are broad due to rotamers

1-(1-Acetyl-2-*tert*-butyloxycarbonyl)hydrazino-1-deoxy-2,3,5-tri-*O*-benzyl-4-methanesulfonyl-L-ribitol (**24**)

The crude alcohol **28** (1.15 mmol) was dissolved in anhydrous diethyl ether (18.4 mL) and cooled to 0 °C. Triethyl amine (0.37 mL, 2.65 mmol, 2.3 eq) and then slowly methanesulfonyl chloride (0.11 mL, 1.42 mmol, 1.2 eq) were added under a nitrogen atmosphere. The mixture was stirred for 2h 20 min (TLC analysis was performed in dichloromethane/ethyl acetate 3:1) and then quenched with water, diluted with ethyl acetate, washed with water and brine, dried over $MgSO_4$, filtered and concentrated under reduced pressure to give the crude product **24** (702 mg).

R_f (pentane/ethyl acetate 2:1) 0.25; ^{1}H-NMR (400 MHz, $CDCl_3$) δ_H 7.41 – 7.09 (m, 15H, ArH), 4.82 – 4.41 (m, 8H), 4.04 – 3.66 (m, 4H), 3.02 (s, 3H), 2.08 – 1.91 (m, 3H), 1.45 (s, 9H). HRMS(ESI): calcd. for $C_{34}H_{44}N_2O_9SH$ 657.2846 found 657.2846

NMR signals are broad due to rotamers

(3*R*,4*S*,5*R*)-1-*N*-acetyl-3-benzyloxymethyl-4,5-di-*O*-benzyl-4,5-dihydroxyhexahydropyrizadine (25)

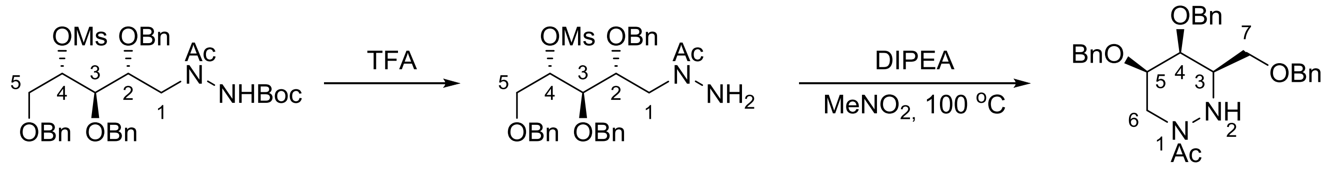

24 **29** **25**

1-(1-Acetyl)hydrazino-1-deoxy-2,3,5-tri-*O*-benzyl-4-methanesulfonyl-L-ribitol (29)

Trifluoroacetic acid (7.0 mL) was added to the crude Boc-protected amine **24** (1.15 mmol) under nitrogen atmosphere. The mixture was stirred for 2h 40min at room temperature and then concentrated under reduced pressure, redissolved in dichloromethane and sat. aq $NaHCO_3$ and the aqueous phase extracted with dichloromethane. The combined organic phases were dried over $MgSO_4$, filtered and concentrated under reduced pressure to give the crude product **29** (578 mg) as a 1:1.3 mixture of rotamers.

R_f (dichlromethane/ethyl acetate 4:1) 0.24; ^{1}H-NMR (400 MHz, $CDCl_3$) δ_H 7.40 – 7.17 (m, 35.3H, Ar*H*), 4.98 (q, *J* 4.9 Hz, 1.2H), 4.93 (td, *J* 5.7 Hz, *J* 3.7 Hz, 1H), 4.76 – 4.67 (m, 4.7H), 4.64 (d, *J* 11.6 Hz, 1.2H), 4.59 (d, *J* 11.5 Hz, 1H), 4.58 – 4.46 (m, 5.8H), 4.44 (d, *J* 11.5 Hz, 1H), 4.04 (dt, *J* 9.3 Hz, *J* 3.1 Hz, 1.1H), 4.00 – 3.91 (m, 3.5H), 3.86 – 3.74 (m, 6.5H), 3.69 (dd, *J* 14.4 Hz, *J* 7.8 Hz, 1.3H), 3.45 (dd, *J* 14.9 Hz, *J* 2.8 Hz, 1H), 3.04 (s, 3.5H, CH_3SO_3 major), 3.01 (s, 2.9H, CH_3SO_3 minor), 2.12 (s, 3.5H, C*H*$_3$), 2.10 (s, 2.7H, C*H*$_3$). ^{13}C-NMR (100 MHz, $CDCl_3$) δ_C 173.8, 170.3, 137.6, 137.5, 137.5, 137.4, 136.8, 128.7, 128.7, 128.6, 128.6, 128.6, 128.6, 128.5, 128.4, 128.3, 128.3, 128.2, 128.1, 128.1, 128.0, 128.0, 128.0, 127.5, 81.1, 80.8, 78.3, 78.3, 77.4, 77.1, 75.2, 74.2, 74.0, 73.6, 73.6, 73.0, 72.6, 69.0, 68.9, 52.3, 51.0, 38.9, 38.8, 29.8, 21.3, 20.9. HRMS(ESI): calcd. for $C_{29}H_{36}N_2O_7H$ 557.2321 found 557.2317

Complicated spectrum due to rotamers

(3*R*,4*S*,5*R*)-1-*N*-acetyl-3-benzyloxymethyl-4,5-di-*O*-benzyl-4,5-dihydroxyhexahydropyrizadine (25)

The crude mesylate **29** (1.15 mmol) was dissolved in anhydrous nitromethane (10 mL) and added DIPEA (0.6 mL, 3.51 mmol, 3 eq) under nitrogen atmosphere. The reaction was heated to 100 °C, stirred for 4h 45 min and then cooled to room temperature and concentrated under reduce pressure. The crude mixture was purified by flash column chromatography (dichloromethane/ethyl acetate 20:1 -> 4:1) to give the product **25** (388 mg, 73 % over 4 steps)

R_f (dichloromethane/ethyl acetate 20:1) 0.31; $[\alpha]_D^{293K}$ - 9.2 (c 1.0, $CHCl_3$); ^{1}H-NMR (400 MHz, $CDCl_3$) δ_H 7.43 – 7.15 (m, 15H, Ar*H*), 4.98 (d, *J* 11.0 Hz, 1H, C*H*HPh), 4.78 (bs, 1H, H6a), 4.71 (d, *J* 11.9 Hz, 1H, C*H*HPh), 4.61 (d, 1H, CH*H*Ph), 4.56 (d, 1H, CH*H*Ph), 4.52 (d, *J* 11.8 Hz, 1H, C*H*HPh), 4.47 (d, 1H, CH*H*Ph), 3.97 (s, 1H, H4), 3.68 (bs, 1H, N*H*), 3.58 – 3.48 (m, 2H, H7a, H5), 3.43 (t, $J_{7a,7b}$ = $J_{7b,3}$ 8.6 Hz, 1H, H7b), 3.03 (bs, 1H, H6b), 2.94 (bs, 1H, H3), 2.15 (s, 3H, C*H*$_3$). ^{13}C-NMR (100 MHz, $CDCl_3$) δ_C 172.6 (*C*=O), 138.7 (Ar), 138.2 (Ar), 137.9 (Ar), 128.6 (Ar), 128.6 (Ar), 128.4 (Ar), 128.2 (Ar), 128.0 (Ar), 128.0 (Ar), 127.9 (Ar), 127.5 (Ar), 76.3 (C5), 74.4 (*C*H$_2$Ar), 73.6 (*C*H$_2$Ar), 72.5 (C4), 71.0 (*C*H$_2$Ar), 67.6 (C7), 60.7 (C3), 40.9 (C6), 21.1 (*C*H$_3$). HRMS(ESI): calcd. for $C_{28}H_{32}N_2O_4H$ 461.2435 found 461.2432

Azagalactofagomine (2)

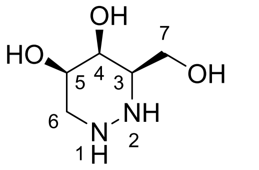

2

The protected compound **25** (139 mg, 842 µmol) was dissolved in ethanol (27 mL) and Pearlman's catalyst (20 %) (113 mg, 161 µmol, 19 %) together with 2 drops of concentrated aq. HCl were added. The mixture was flushed with hydrogen, stirred under a hydrogen atmosphere for 3h 20min and then concentrated under reduced pressure, redissolved in methanol, filtered through Celite® and concentrated again. The crude residue was added 6 M aq. HCl (37 mL) and heated to 100 °C in a closed flask. Left overnight and then concentrated under reduced pressure to give the crude product which was purified by ion exchange chromatography with Amberlite IR 120 H^+ (eluted with 2.5% NH_4OH)and flash column chromatography (EtOH/25% NH_4OH 1:0 -> 20:1) to give the product **2** (67.3 mg, 54 % over 2 steps) as a yellow oil

R_f (EtOH/25% NH_4OH 20:1) 0.18; $[\alpha]_D^{293K}$ + 9.0 (c 1.0, H_2O) Ref: +11.9 (Jensen & Bols 2001); ^{1}H-NMR (400 MHz, D_2O) δ_H 3.96 (bs, 1H, H4), 3.74 (ddd, $J_{H5,H6ax}$ 11.0 Hz, J 5.2 Hz, J 3.1 Hz, 1H, H5), 3.59 (d, $J_{7,3}$ 6.6 Hz, 2H, H7a, H7b), 2.91-2.82 (m, 2H, H3, H6eq), 2.76 (t, $J_{6ax,5}$ 11.5 Hz, 1H, H6ax). ^{13}C-NMR (100 MHz, D_2O) δ_C 67.6 (C4/C5), 66.4 (C4/C5), 60.8 (C3/C7), 60.2 (C3/C7), 46.2 (C6). HRMS(ESI): calcd. for $C_5H_{12}N_2O_3H$ 149.0921 found 149.0920

NMR data was in accordance with previously reported values[6]

NMR spectra

2,3-*O*-isopropylidene-5-*O*-triisopropylsilyl-D-ribofuranose (6):

^{1}H-NMR (400 MHz, $CDCl_3$)

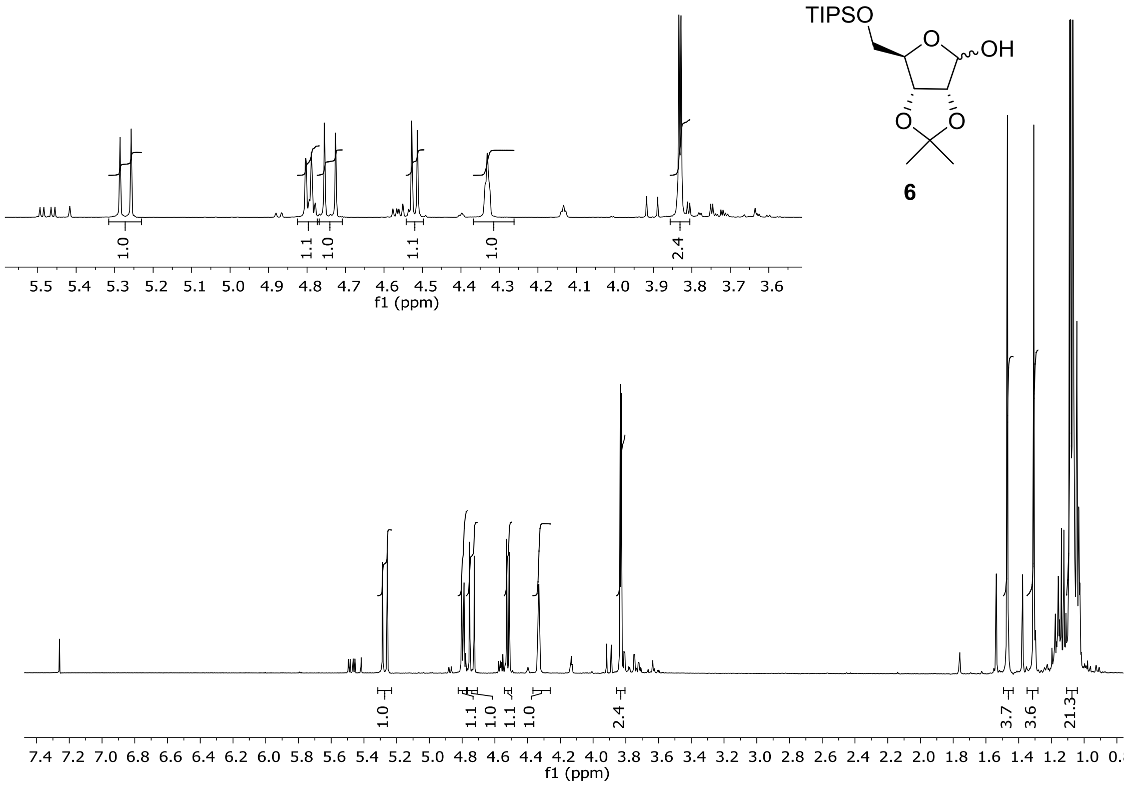

^{13}C-NMR (100 MHz, $CDCl_3$):

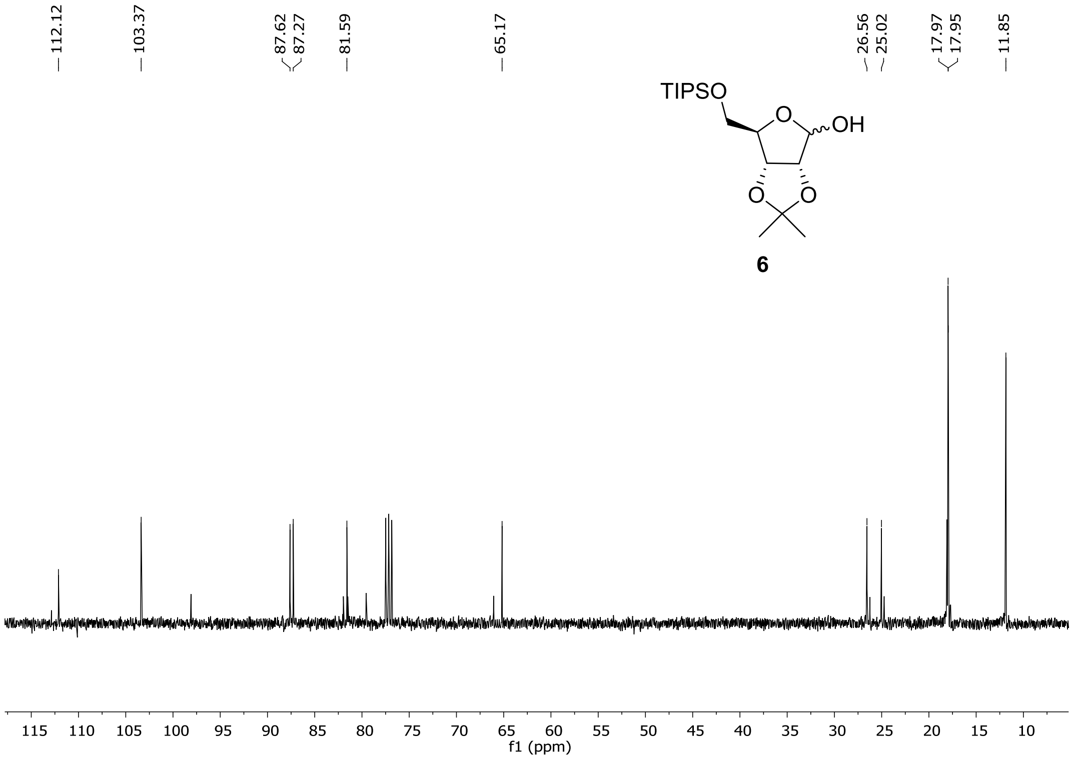

2,3,-*O*-isopropylidene-5-*O*-triisopropylsilyl-D-ribitol (26):

^{1}H-NMR (400 MHz, $CDCl_3$)

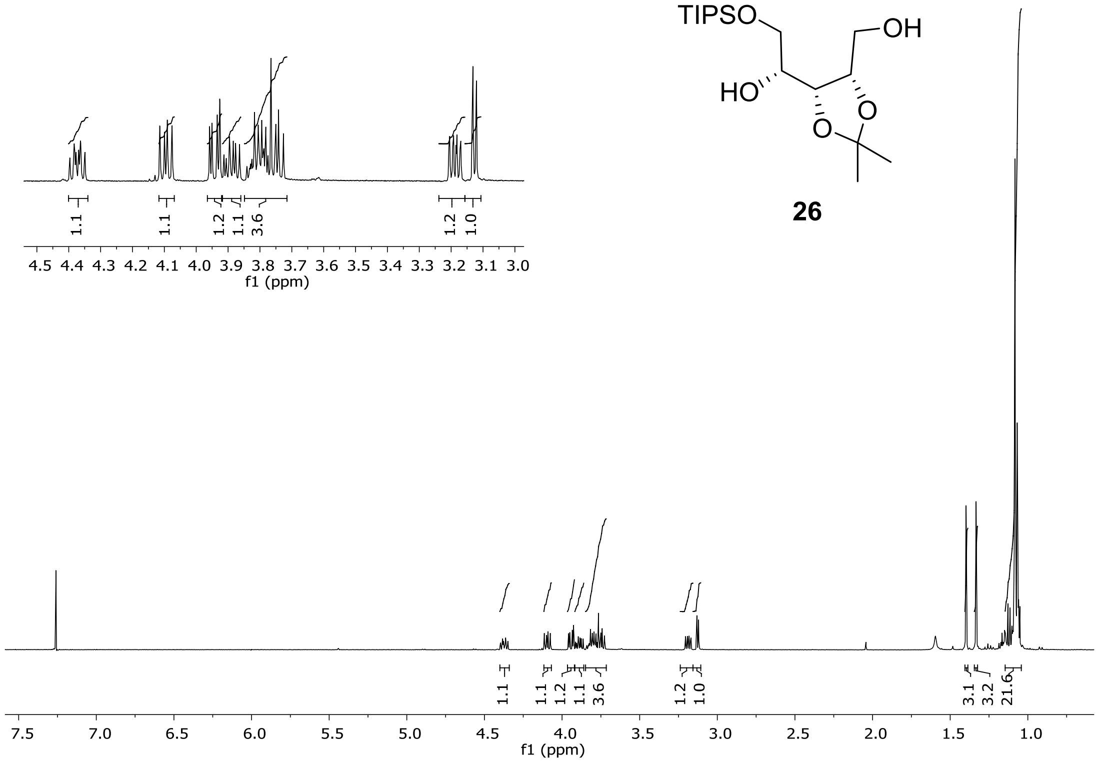

^{13}C-NMR (100 MHz, $CDCl_3$)

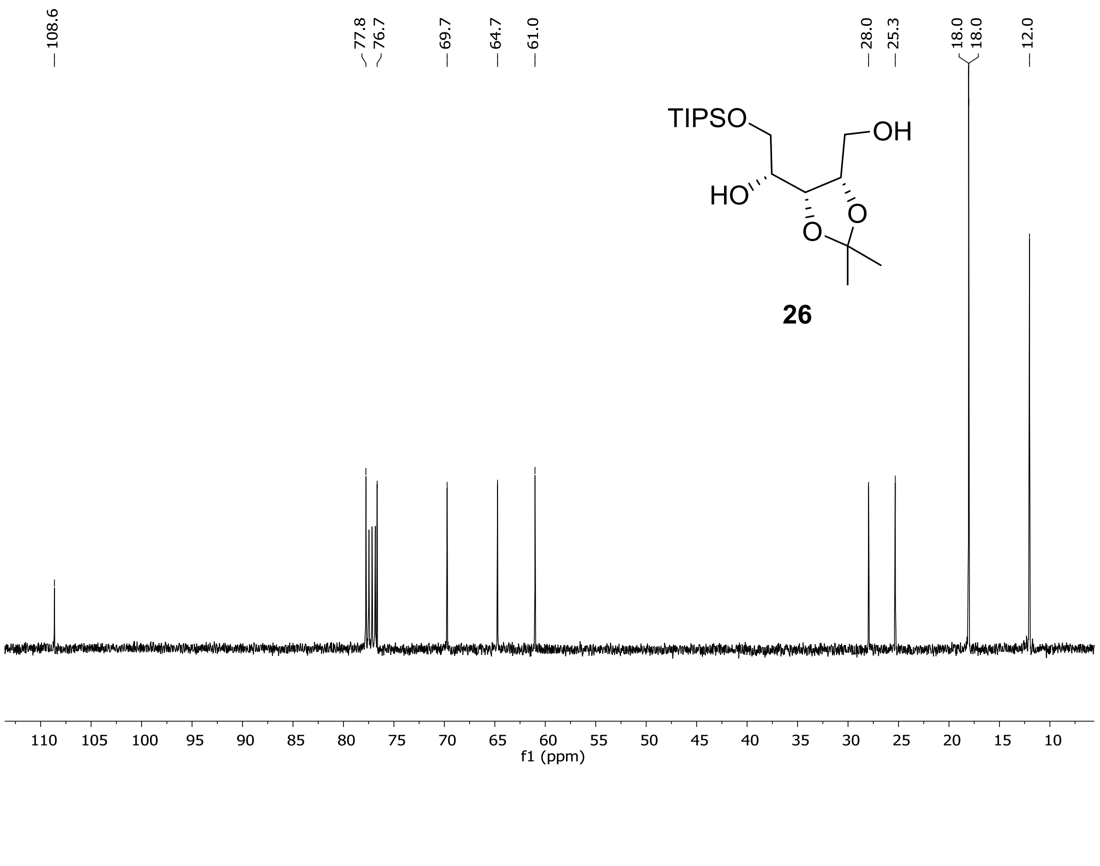

1,4-di-*O*-benzyl-2,3-*O*-isopropylidene-5-*O*-triisopropylsilyl-D-ribitol (7):

^{1}H-NMR (400 MHz, $CDCl_3$)

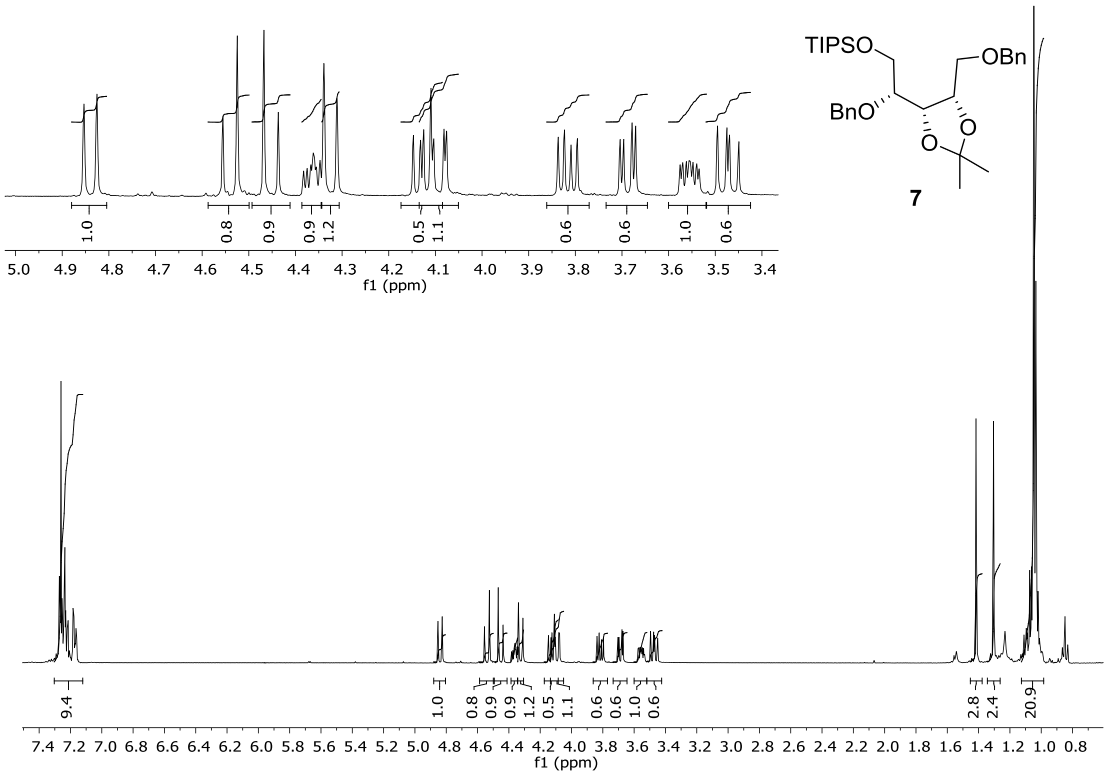

^{13}C-NMR (100 MHz, $CDCl_3$)

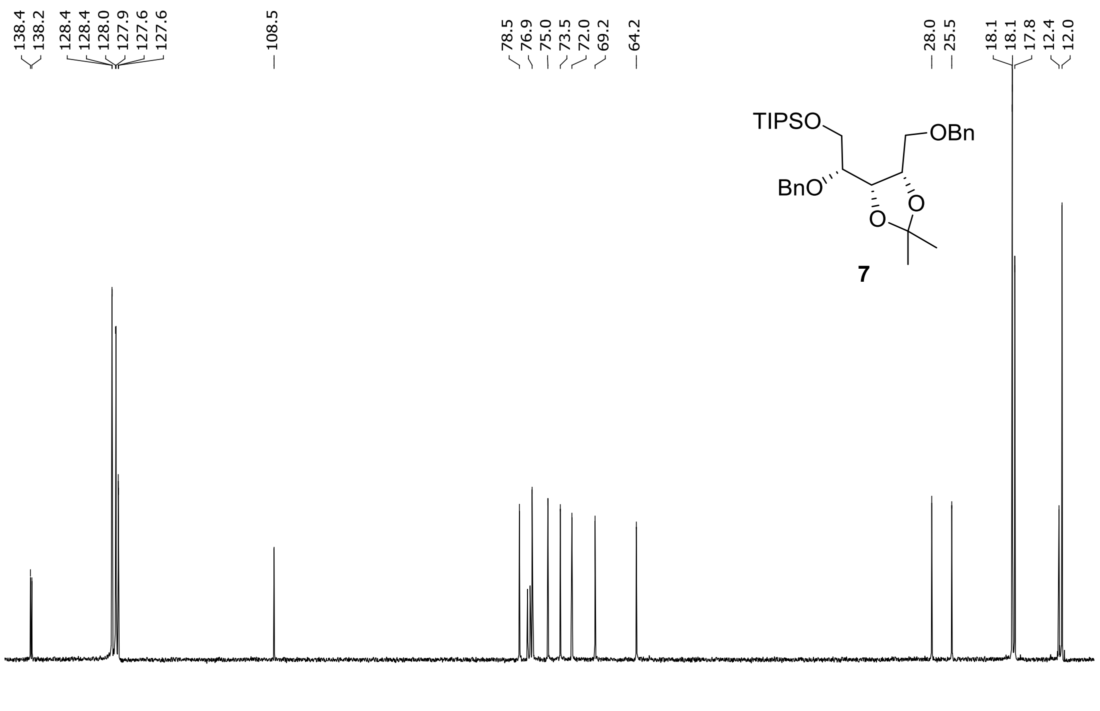

1,4-di-*O*-benzyl-5-*O*-triisopropylsilyl-D-ribitol (8):

^{1}H-NMR (400 MHz, $CDCl_3$)

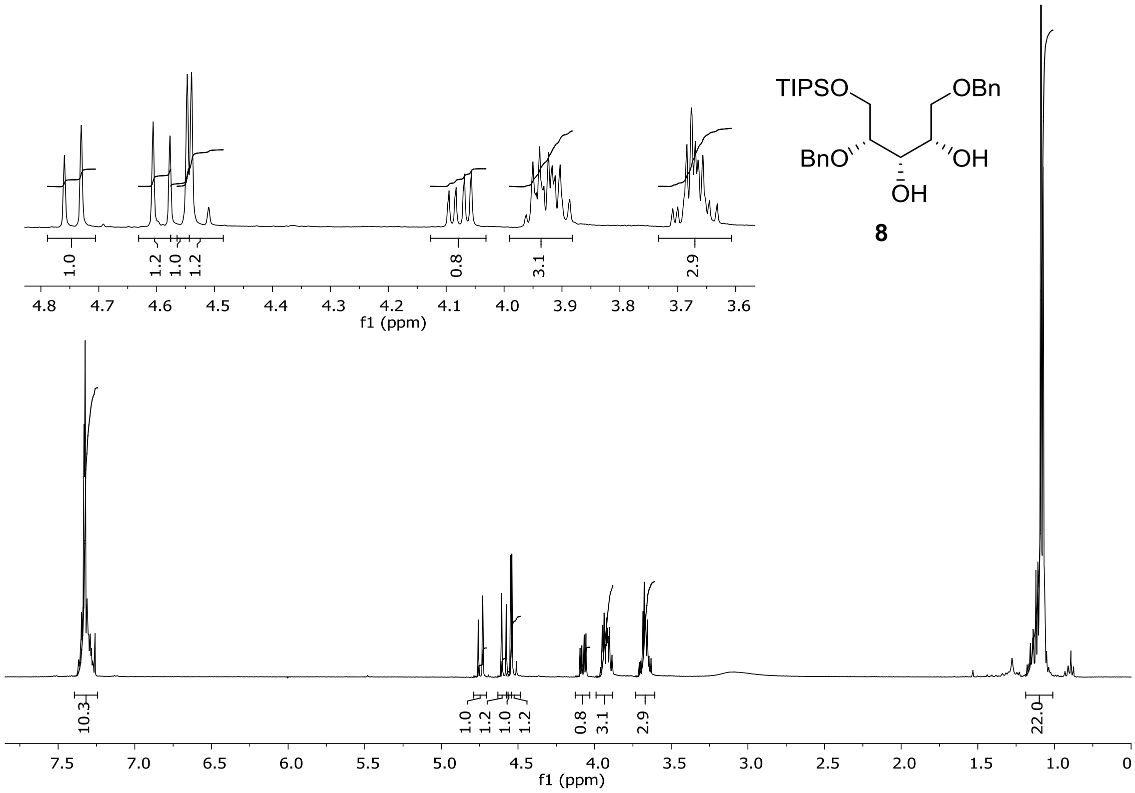

^{13}C-NMR (100 MHz, $CDCl_3$)

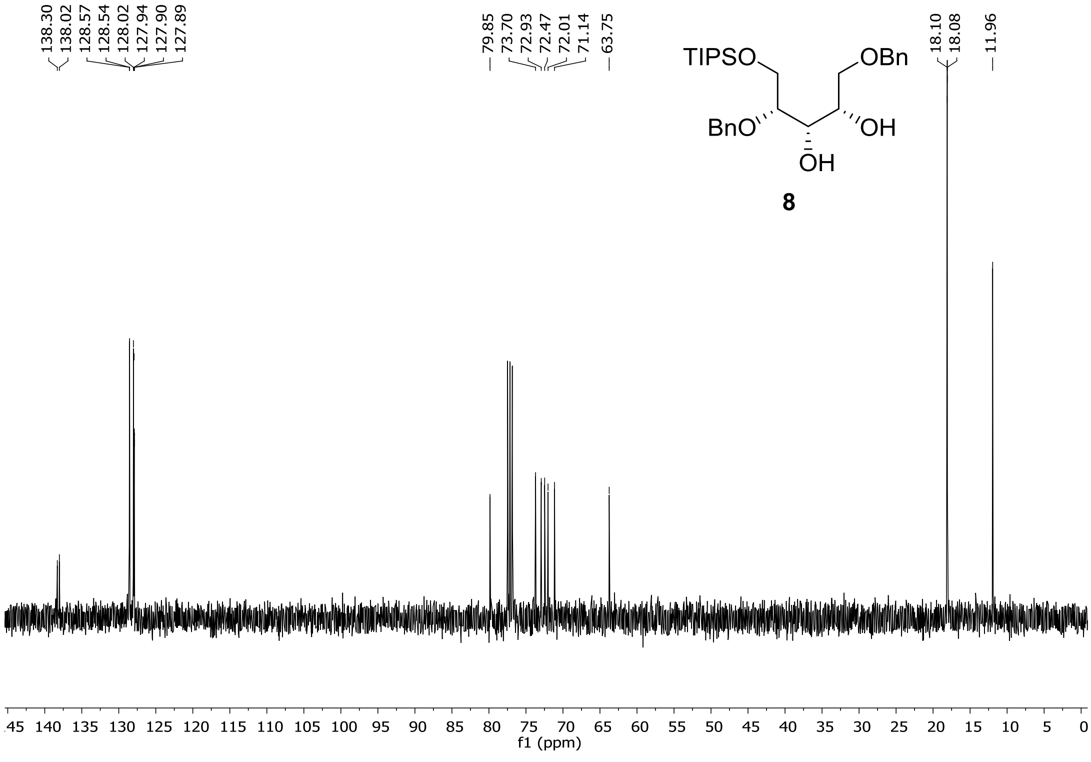

1,4-di-*O*-benzyl-2,3-*O*-benzylidene-5-*O*-triisopropylsilyl-D-ribitol (9 Major):

^{1}H-NMR (400 MHz, CDCl$_3$)

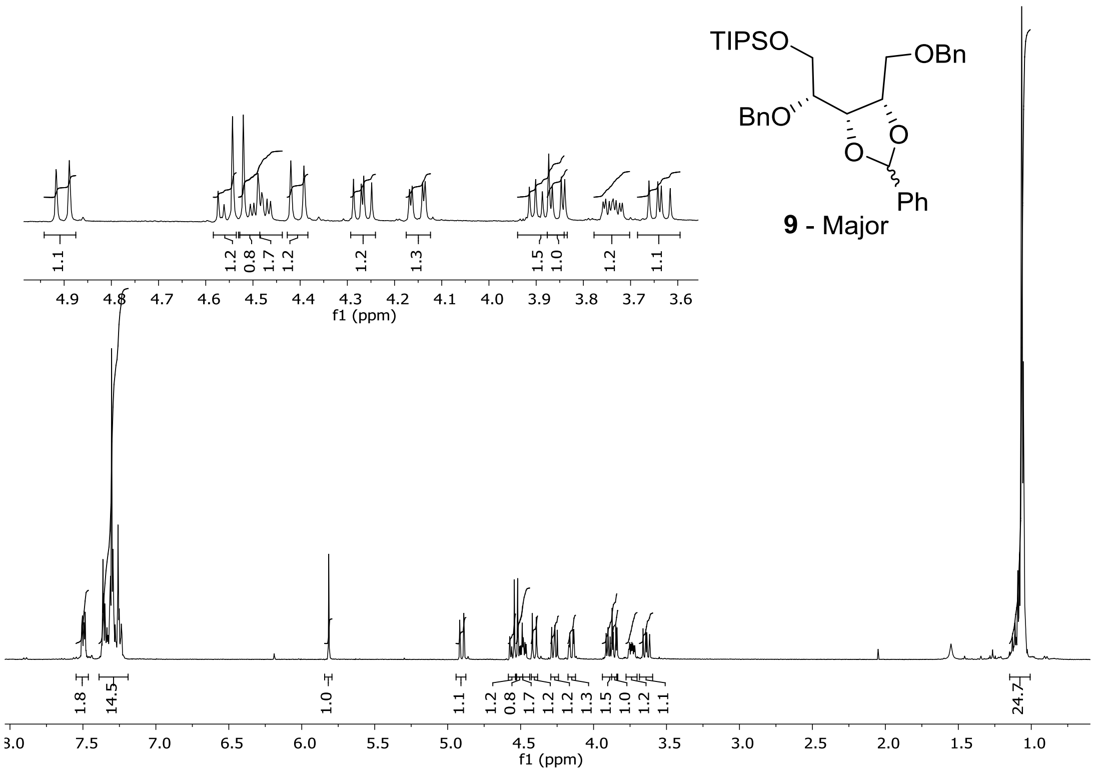

^{13}C-NMR (100 MHz, CDCl$_3$)

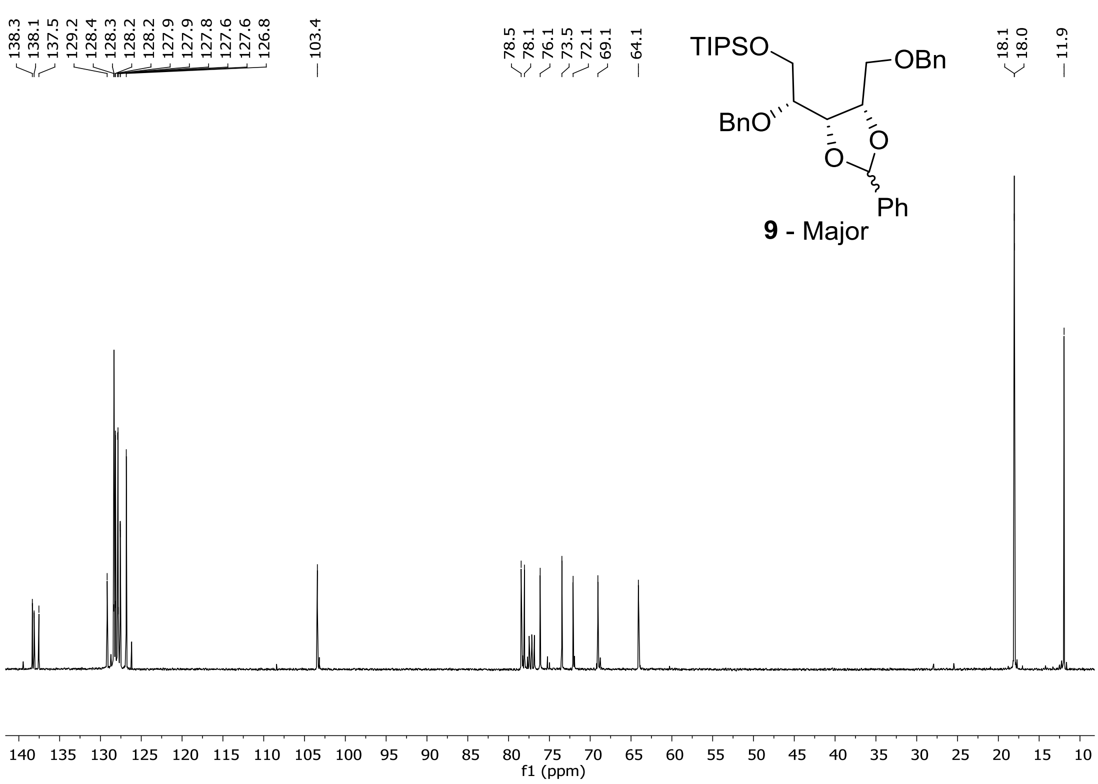

1,4-di-*O*-benzyl-2,3-*O*-benzylidene-5-*O*-triisopropylsilyl-D-ribitol (9 Minor):

^{1}H-NMR (400 MHz, $CDCl_3$)

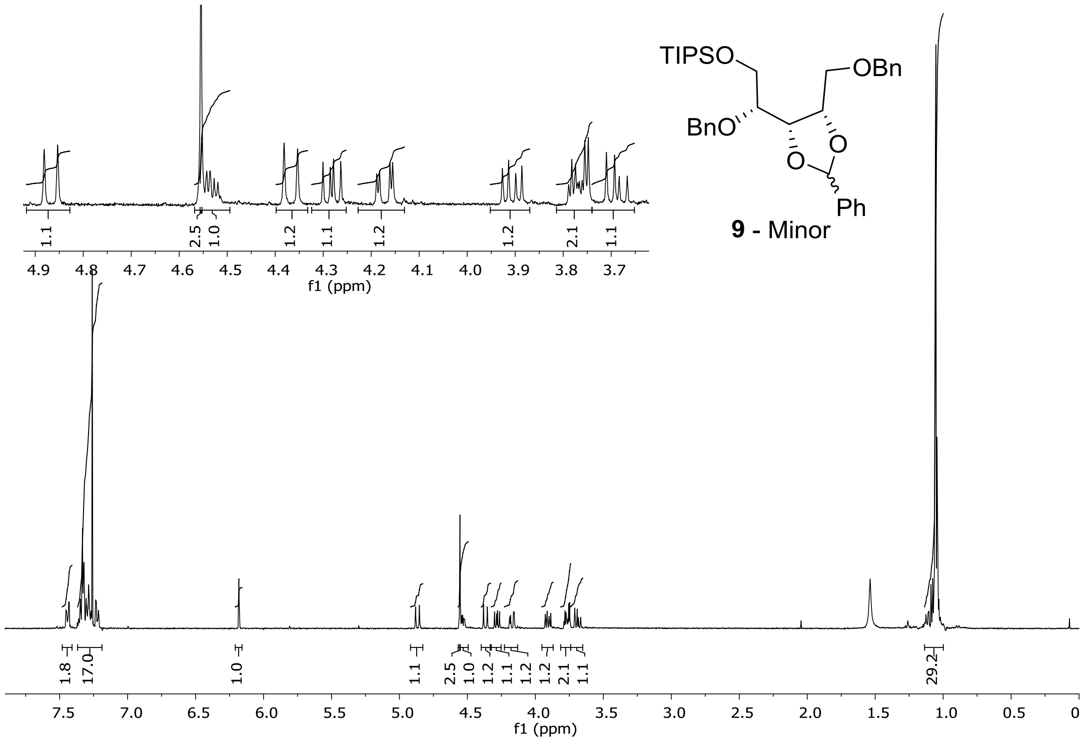

^{13}C-NMR (100 MHz, $CDCl_3$)

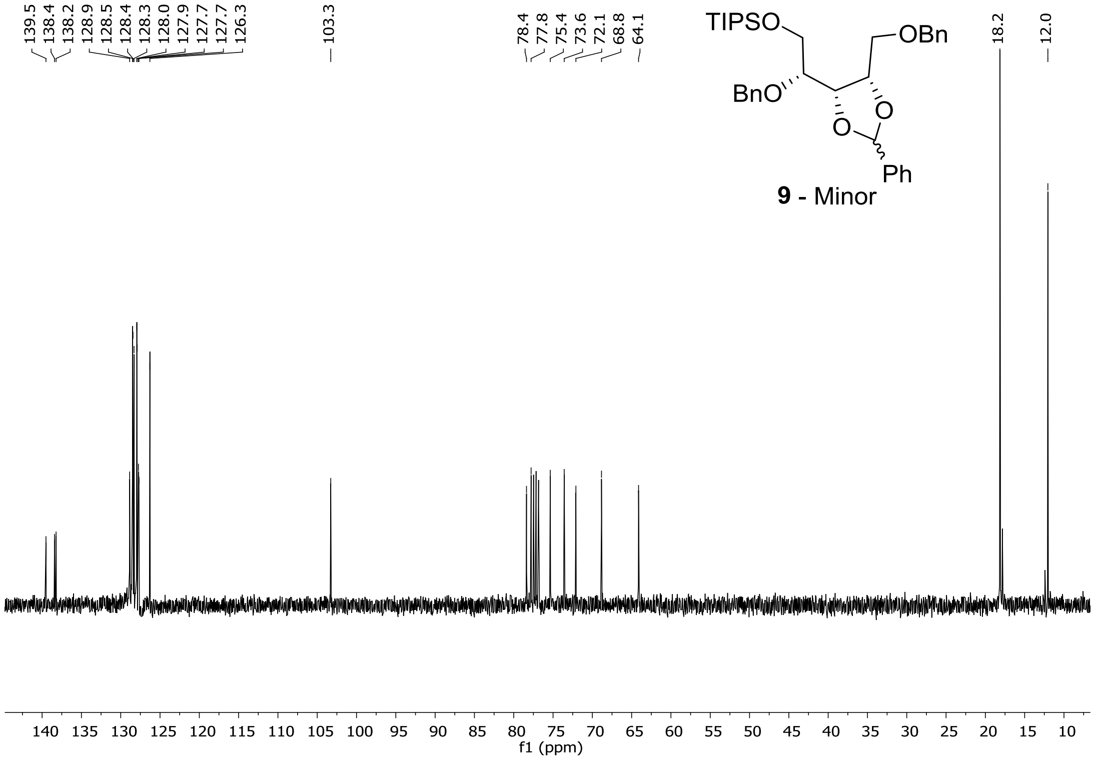

1,3,4-tri-*O*-benzyl-5-*O*-triisopropylsilyl-D-ribitol (10):

^{1}H-NMR (400 MHz, $CDCl_3$)

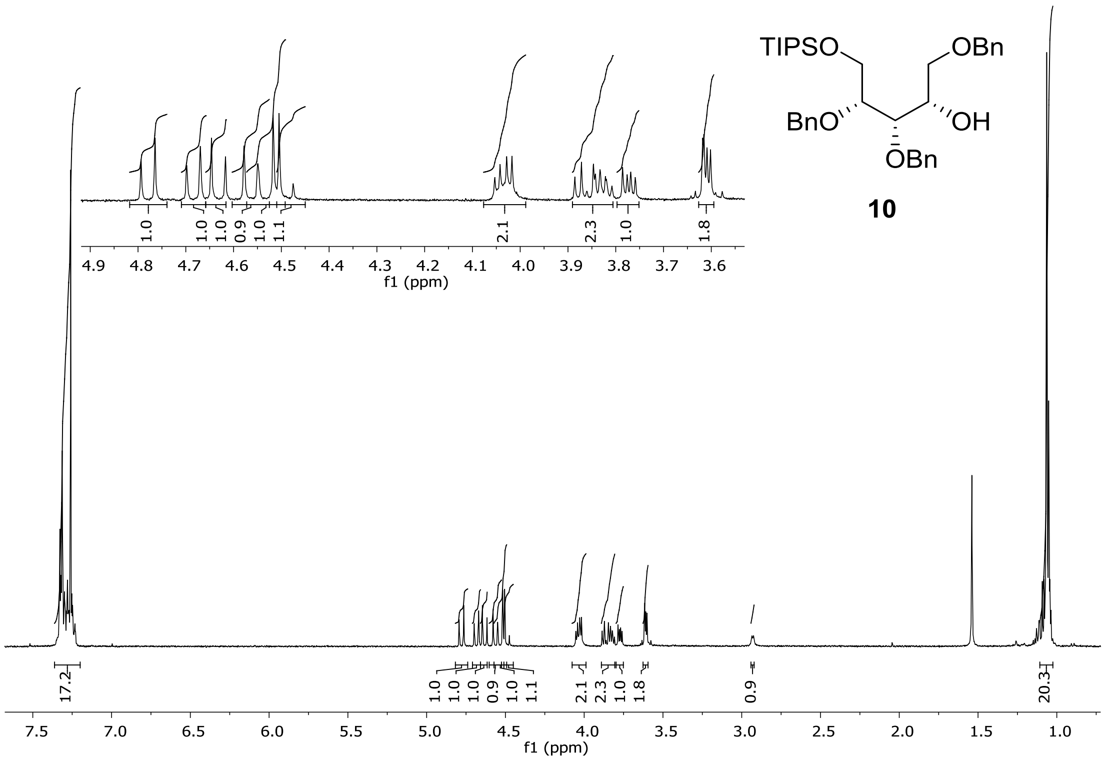

^{13}C-NMR (100 MHz, $CDCl_3$)

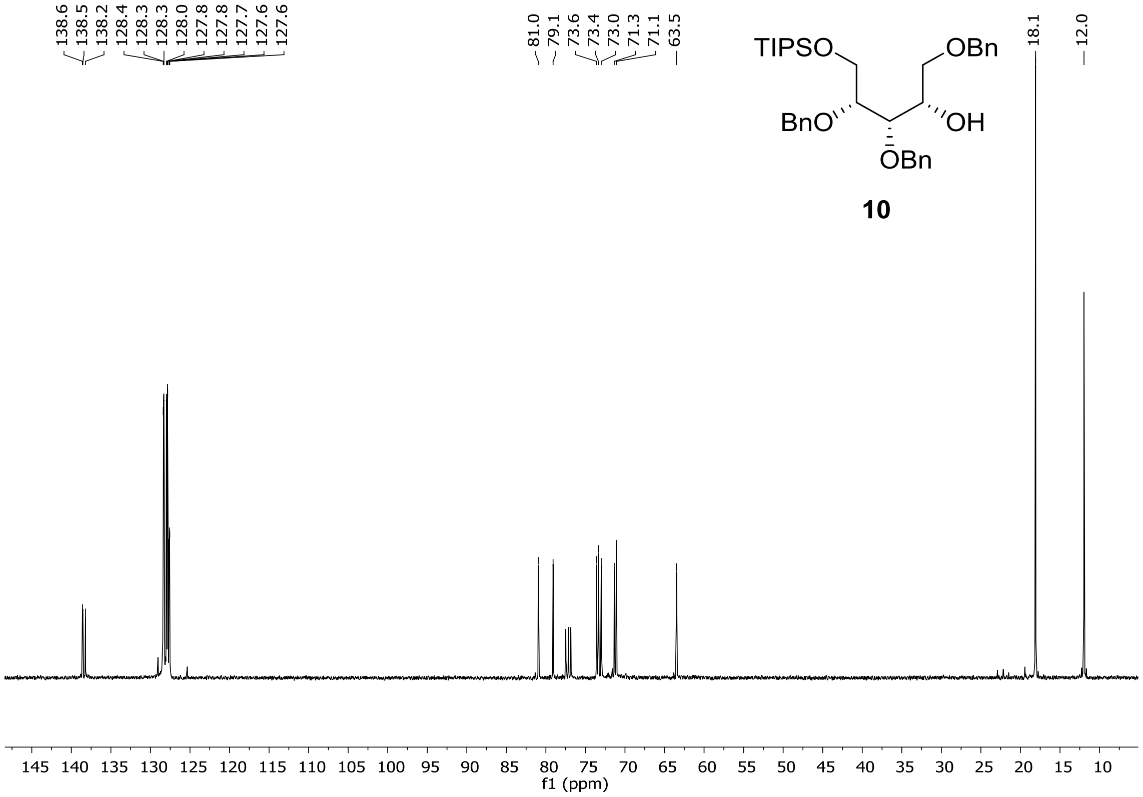

1,3,4-tri-*O*-benzyl-D-ribitol (11):

^{1}H-NMR (400 MHz, $CDCl_3$)

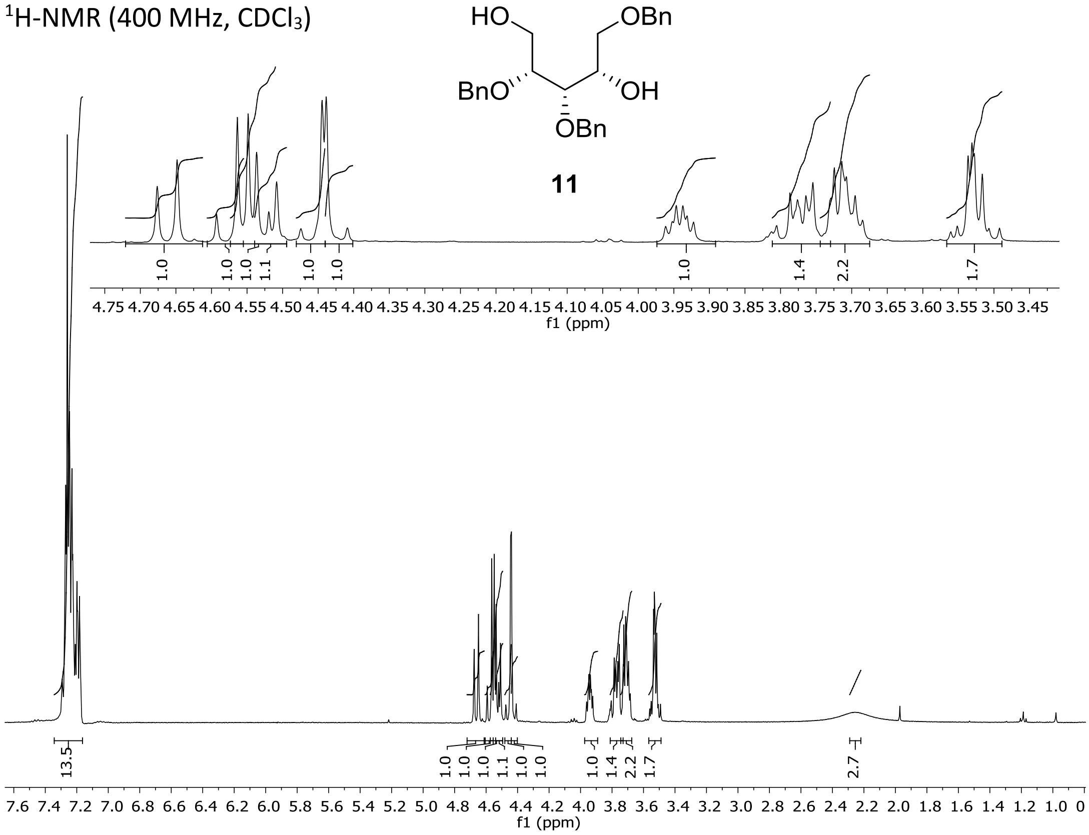

^{13}C-NMR (100 MHz, $CDCl_3$)

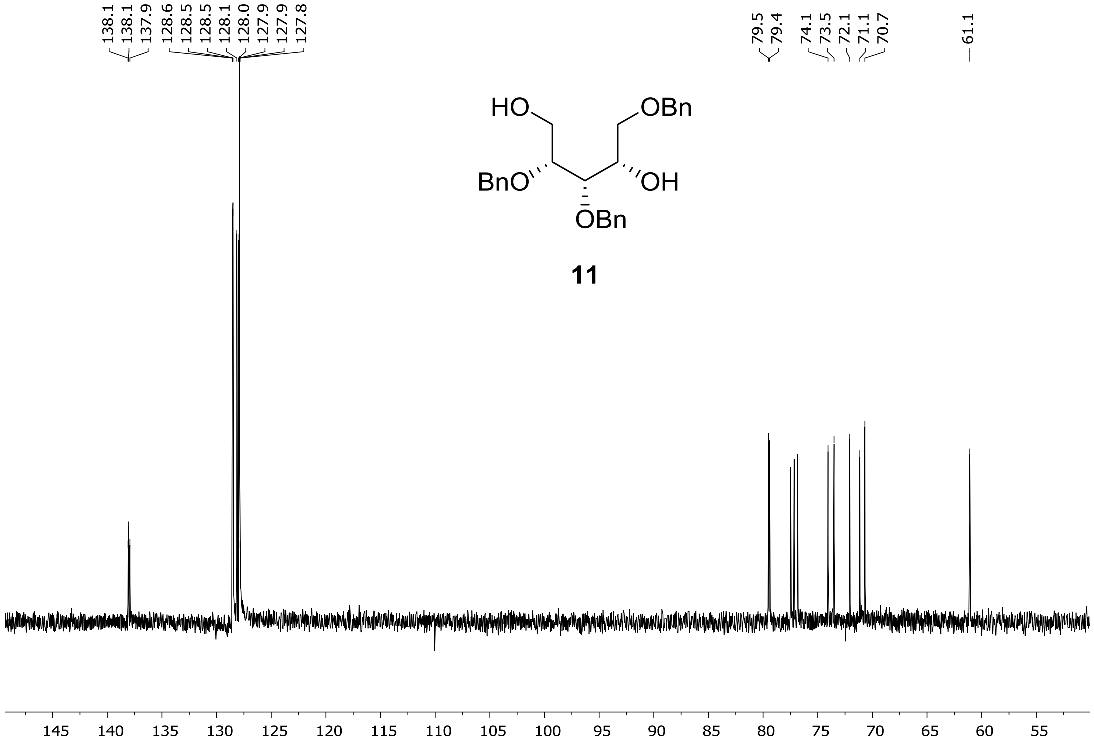

1,3,4-tri-*O*-benzyl-5-*O*-pivaloyl-D-ribitol (12):

^{1}H-NMR (400 MHz, $CDCl_3$)

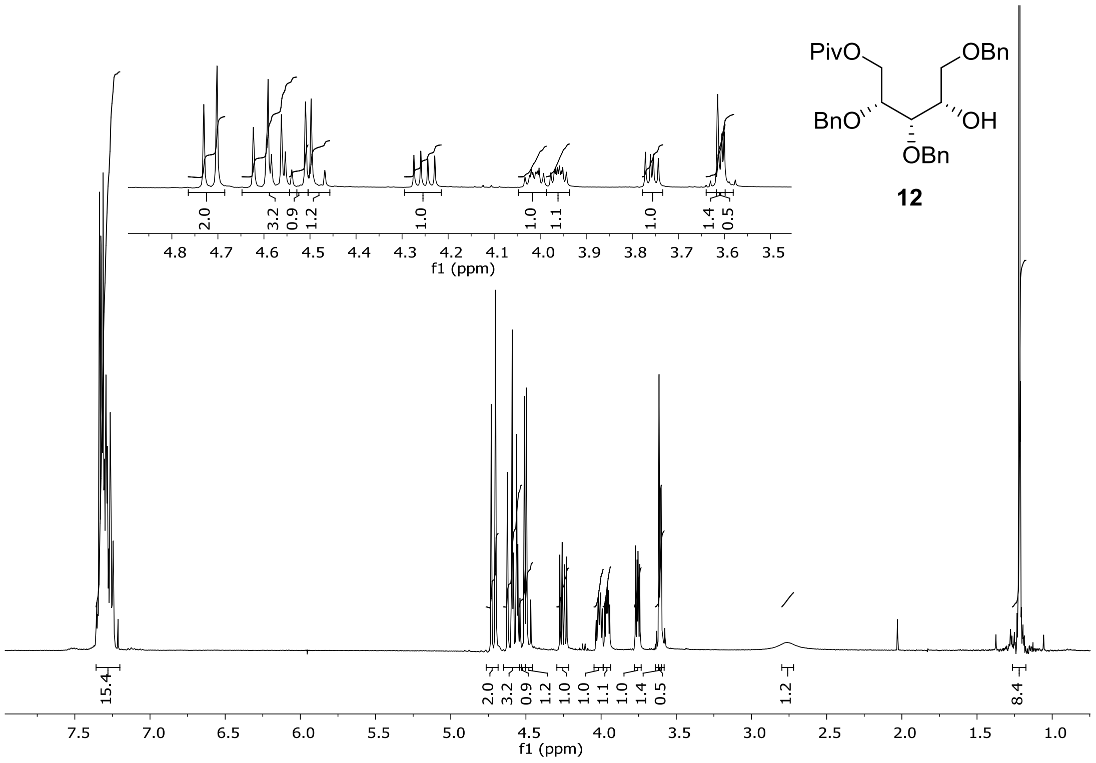

^{13}C-NMR (100 MHz, $CDCl_3$)

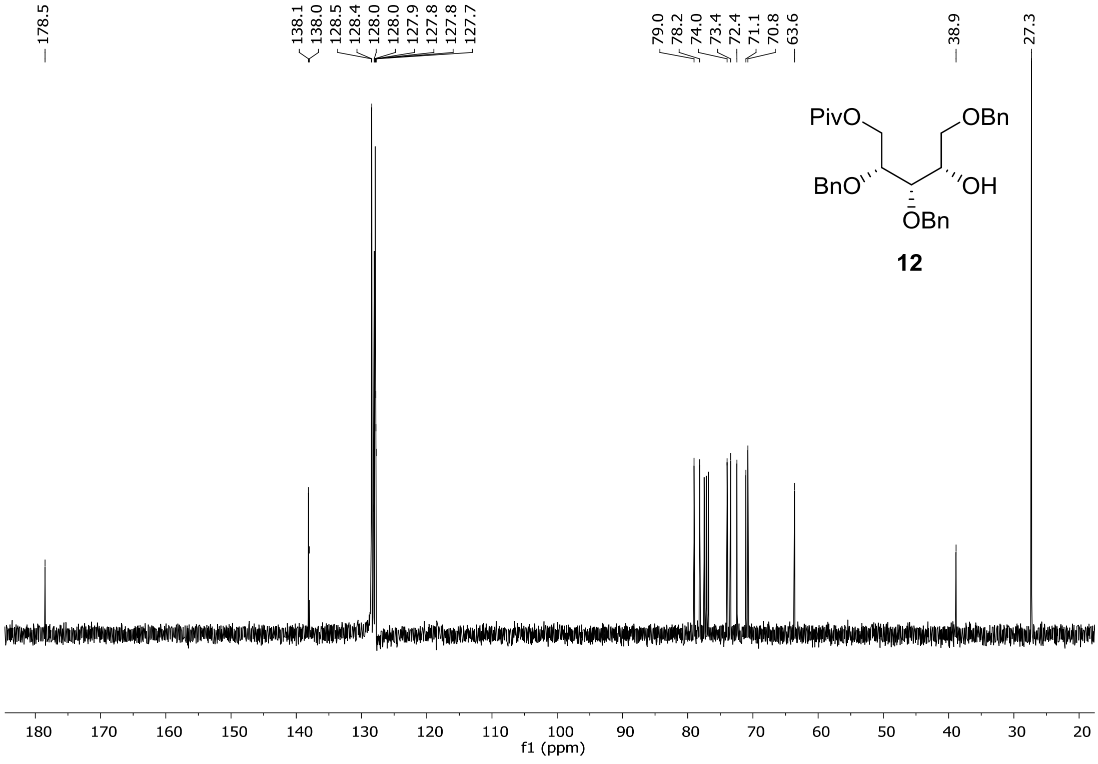

1,3,4-tri-*O*-benzyl-2,5-di-*O*-pivaloyl-D-ribitol (30):

^{1}H-NMR (400 MHz, $CDCl_3$)

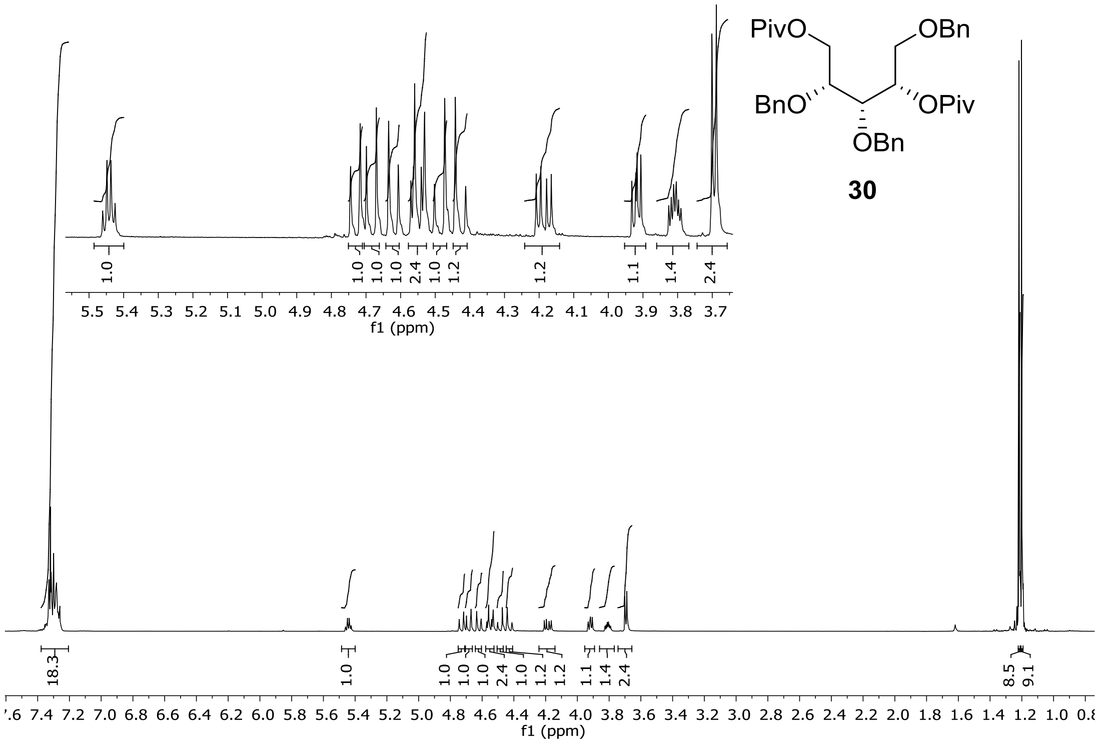

^{13}C-NMR (100 MHz, $CDCl_3$)

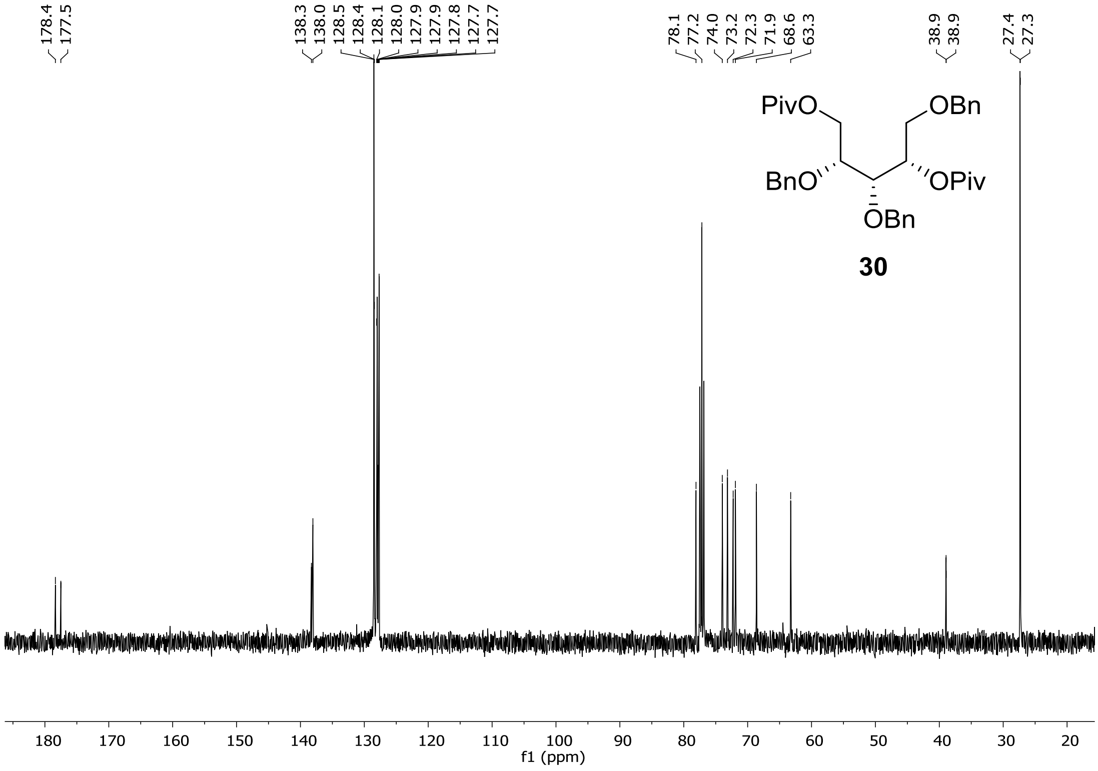

1,3,4-tri-*O*-benzyl-5-*O*-pivaloyl-2-*O*-triflyl-D-ribitol (13):

^{1}H-NMR (400 MHz, $CDCl_3$)

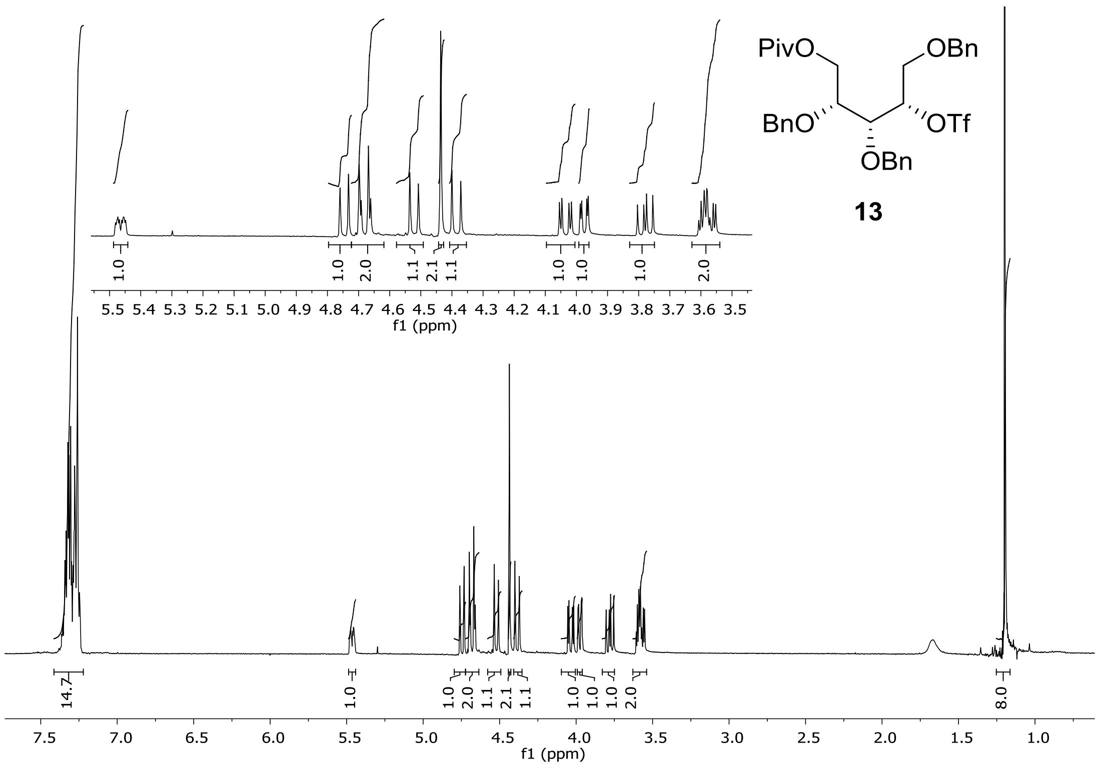

^{13}C-NMR (100 MHz, $CDCl_3$)

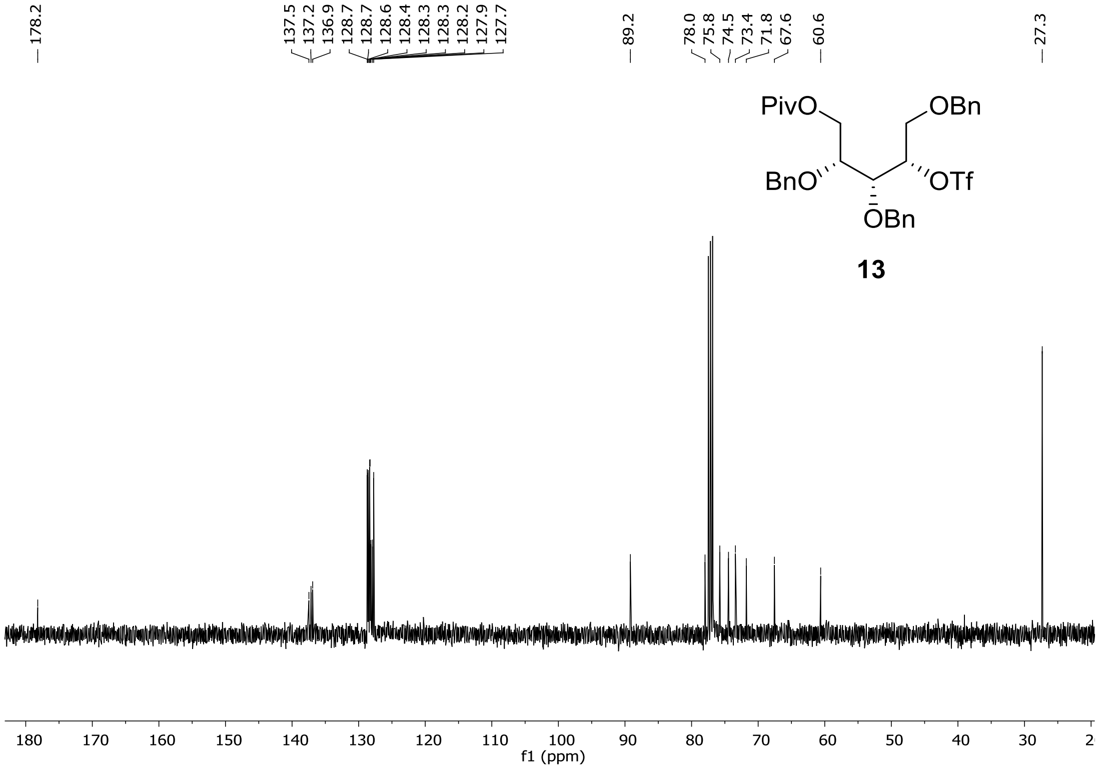

^{19}F-NMR (400 MHz, $CDCl_3$)

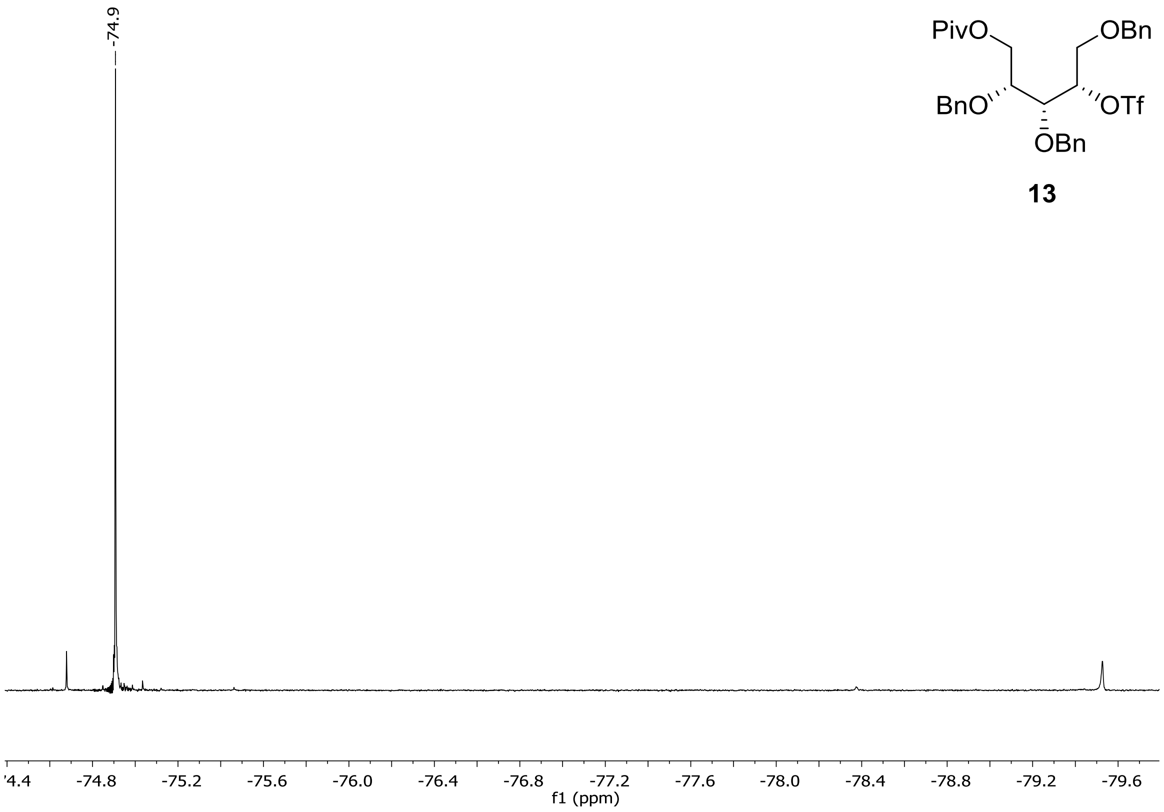

13

1,3,4-tri-*O*-benzyl-2-cyano-2-deoxy-5-*O*-pivaloyl-D-arabinitol (15):

^{1}H-NMR (400 MHz, $CDCl_3$)

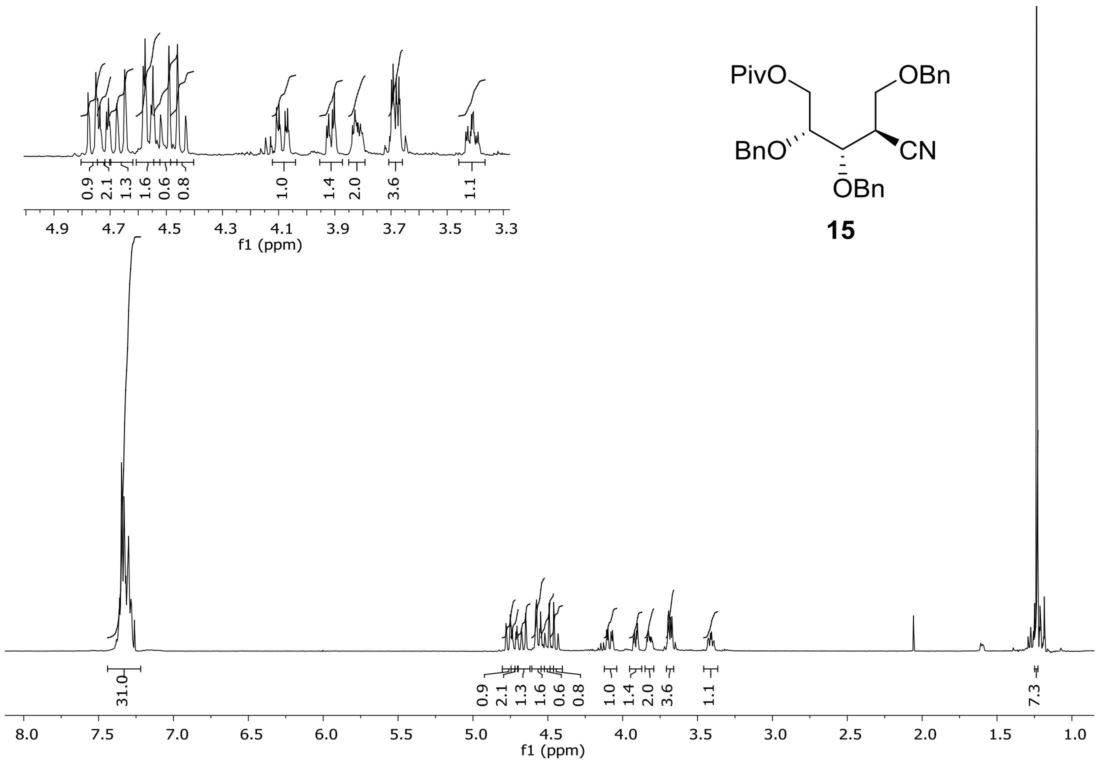

^{13}C-NMR (100 MHz, $CDCl_3$)

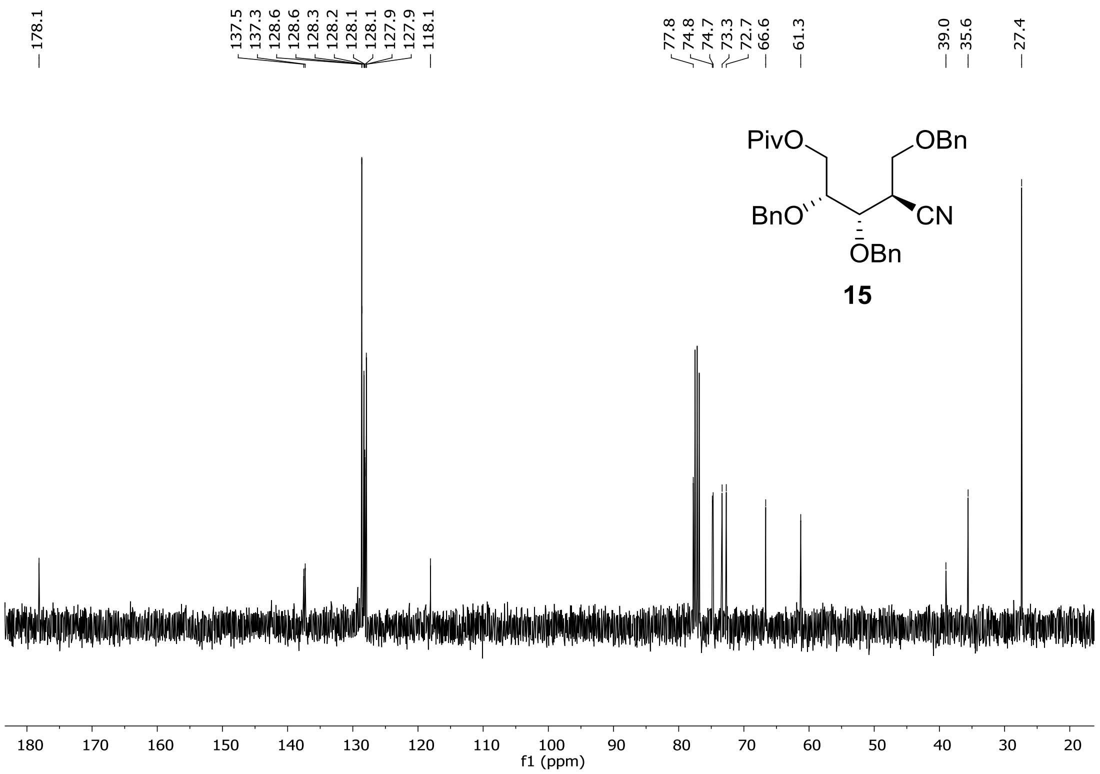

(4*R*,3*S*,*E*)1,3,4-tri-*O*-benzyloxy-5-*O*-pivaloyl-pent-1-ene (14):

^{1}H-NMR (400 MHz, $CDCl_3$)

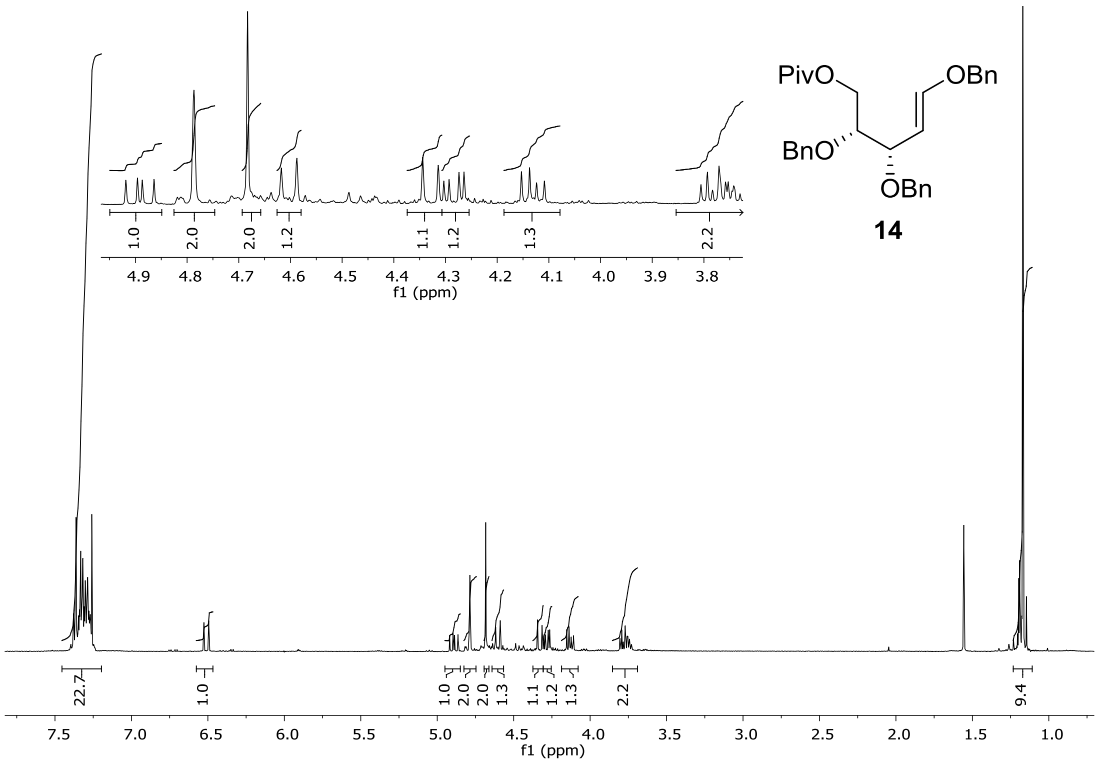

^{13}C-NMR (100 MHz, $CDCl_3$)

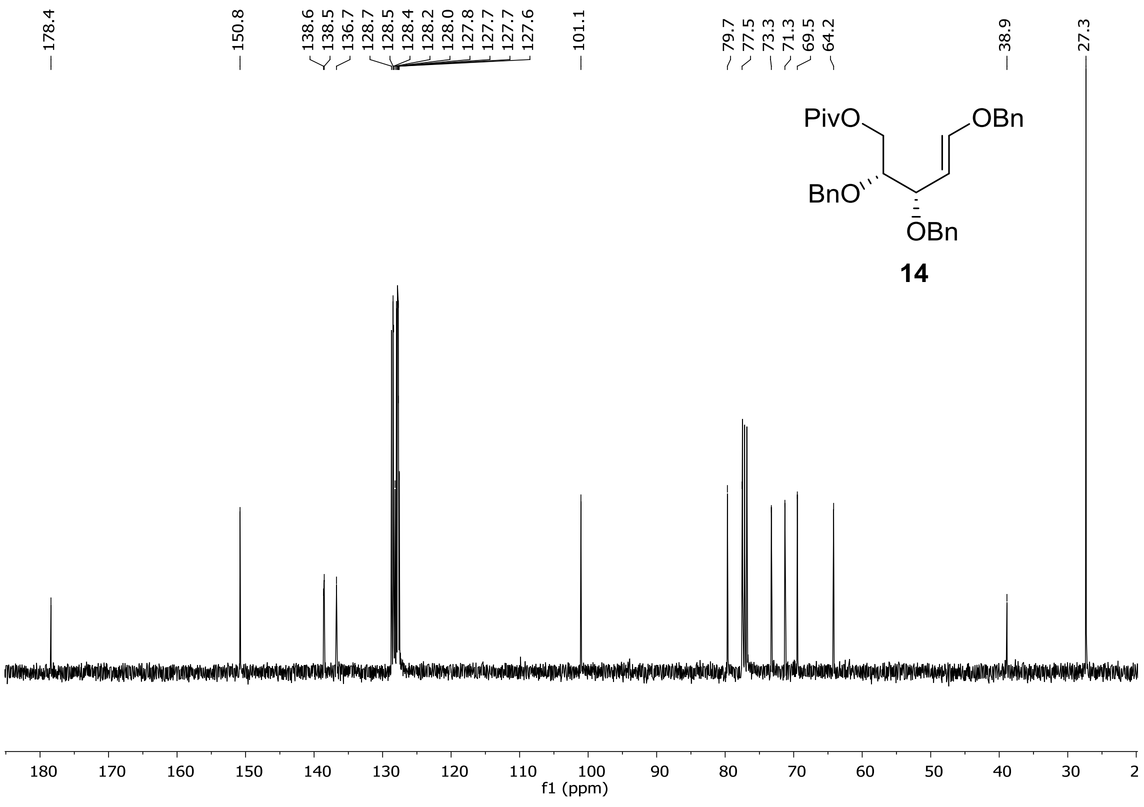

2,3,5-*O*-benzyl-4-(((*tert*-butoxycarbonyl)amino)methyl)-4-deoxy-D-arabinitol (16):

^{1}H-NMR (400 MHz, $CDCl_3$)

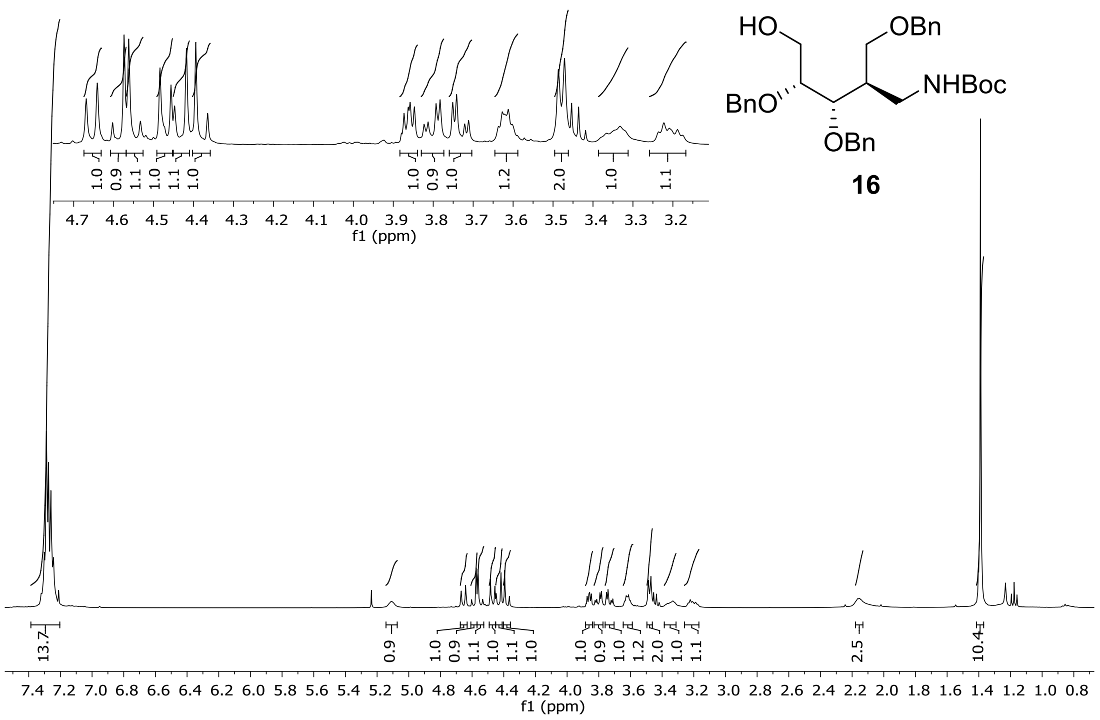

^{13}C-NMR (100 MHz, $CDCl_3$)

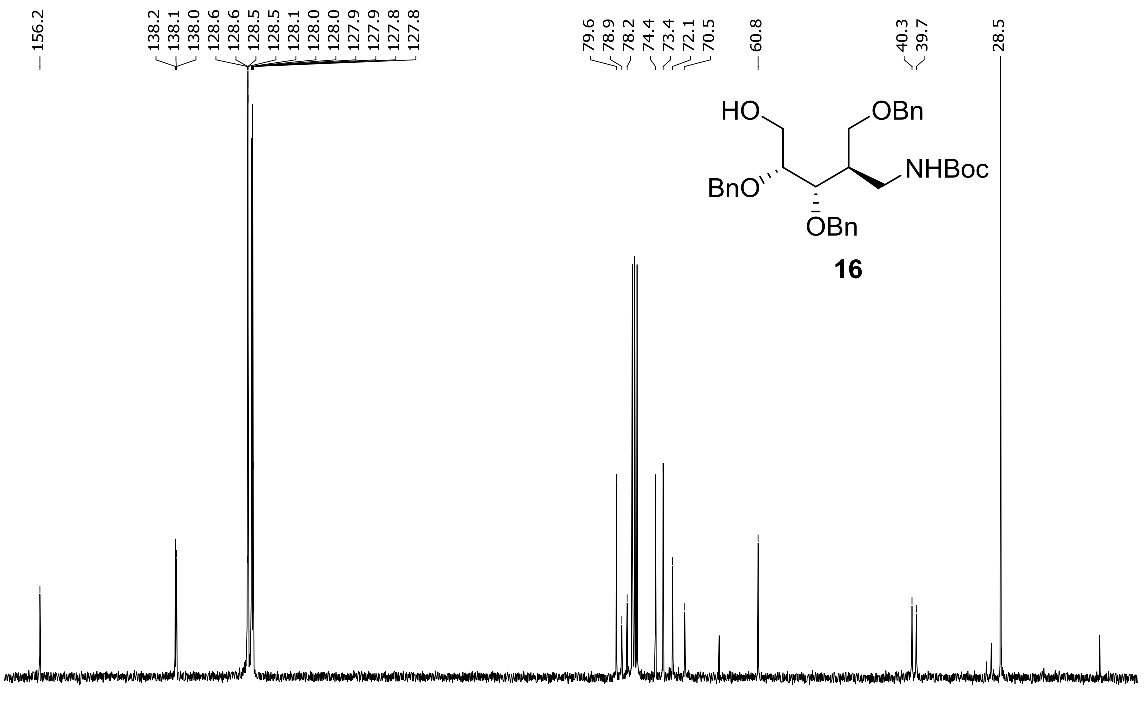

2,3,5-tri-*O*-benzyl-4-(((*tert*-butoxycarbonyl)amino)methyl)-4-deoxy-1-*O*-mesyl-D-arabinitol (27):

^{1}H-NMR (400 MHz, $CDCl_3$)

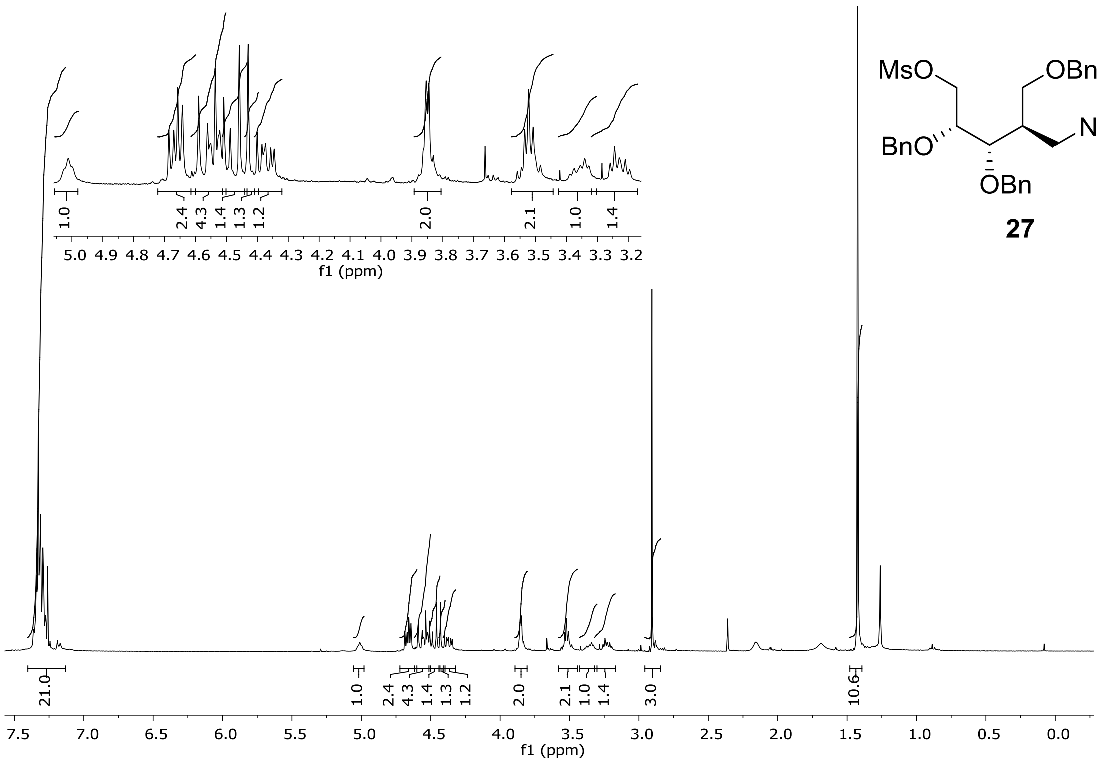

^{13}C-NMR (100 MHz, $CDCl_3$)

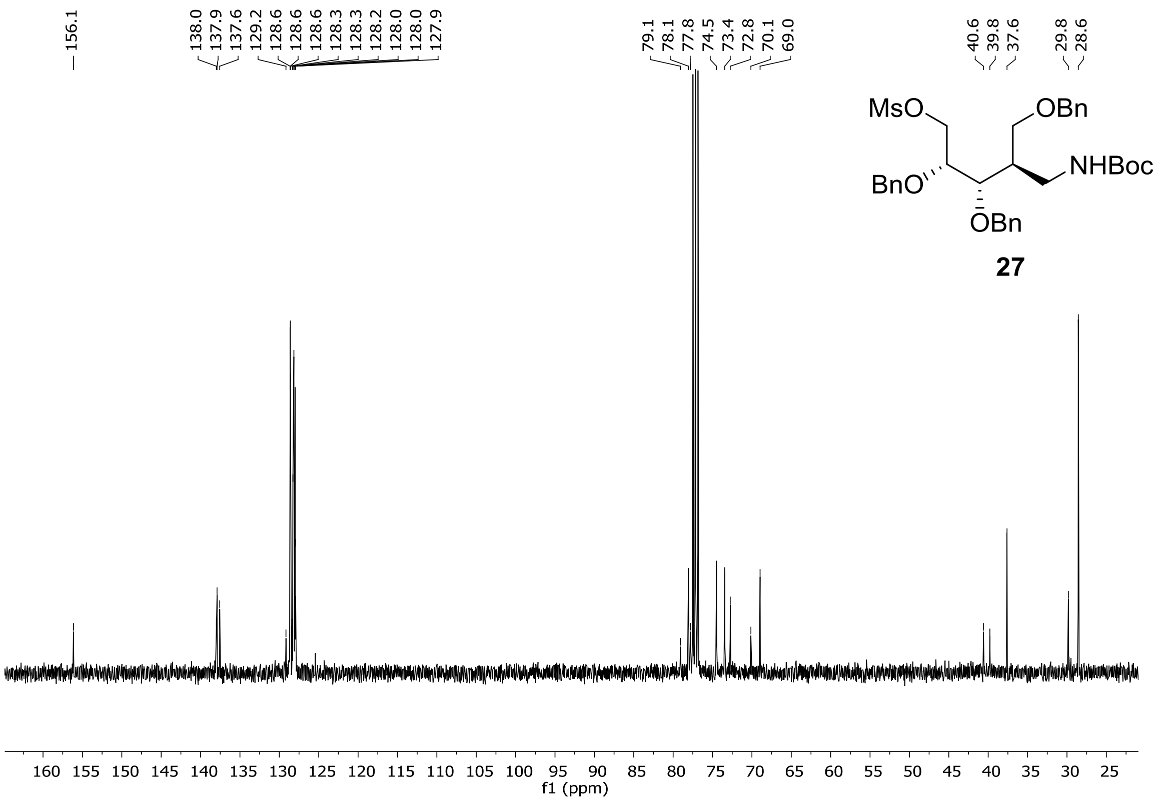

(3*R*,4*S*,5*R*)-3,4-di-*O*-benzyl-5-(benzyloxymethyl)piperidine (17):

^{1}H-NMR (400 MHz, $CDCl_3$)

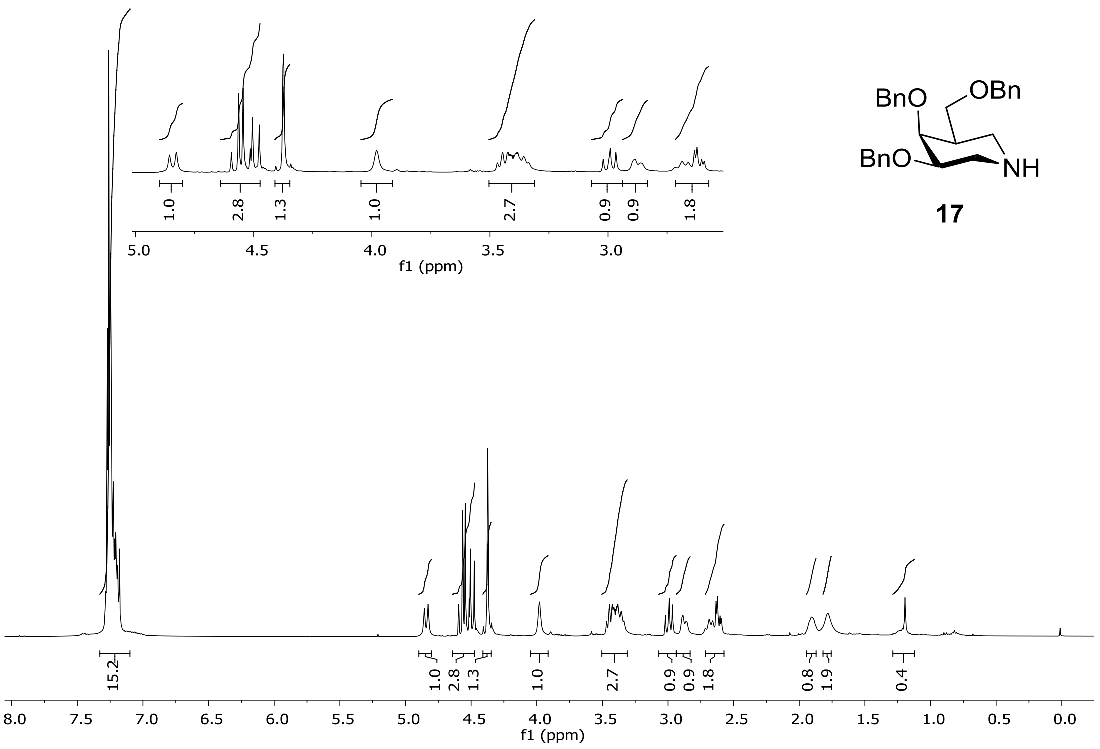

17

^{13}C-NMR (100 MHz, $CDCl_3$)

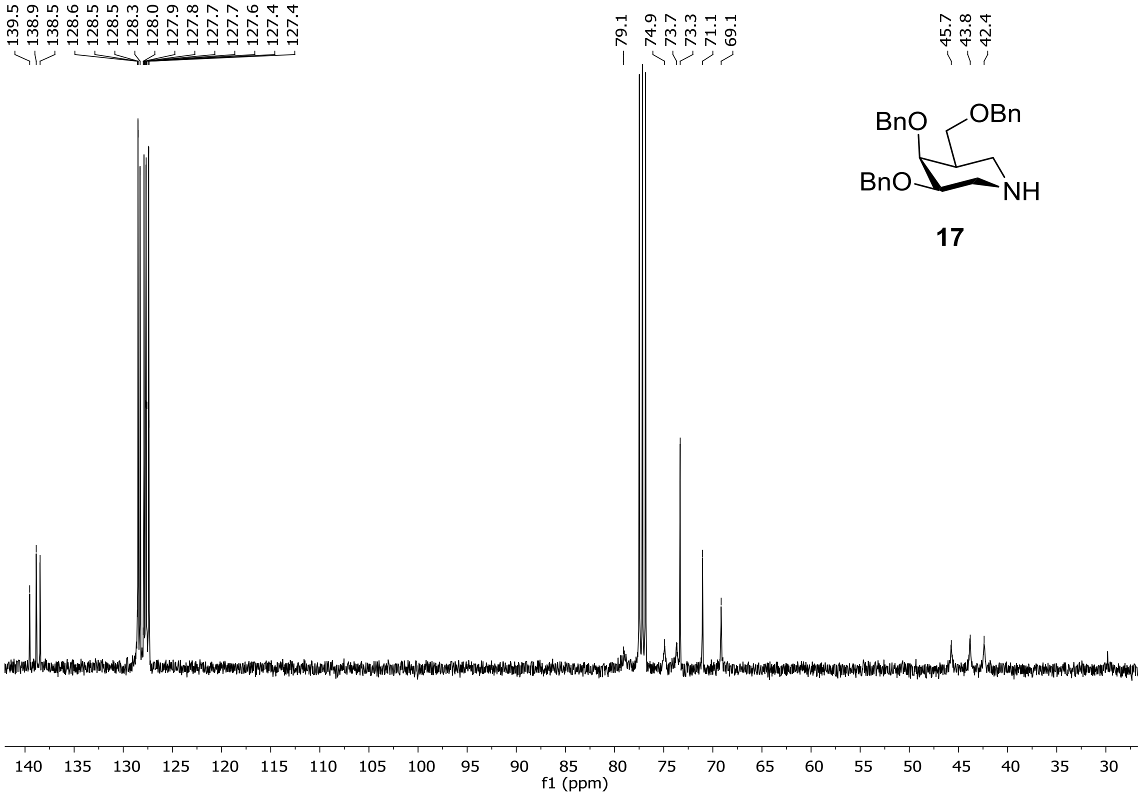

17

Isogalactofagomine(1):

^{1}H-NMR (400 MHz, D_2O)

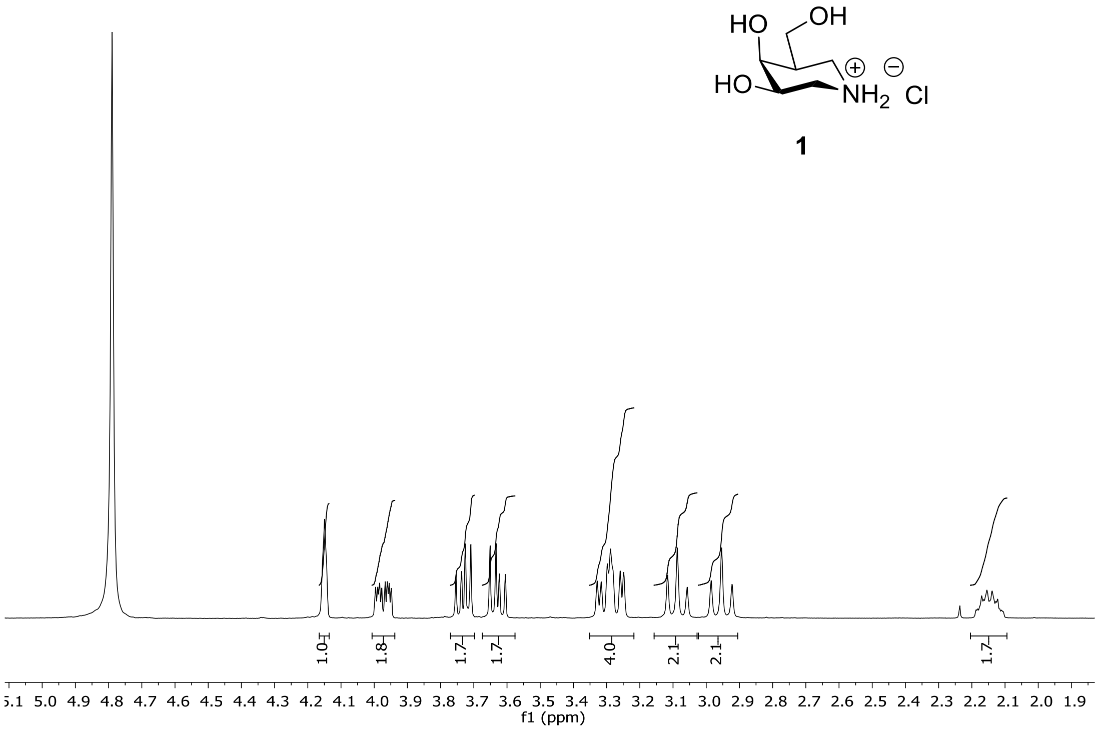

^{13}C-NMR (100 MHz, D_2O)

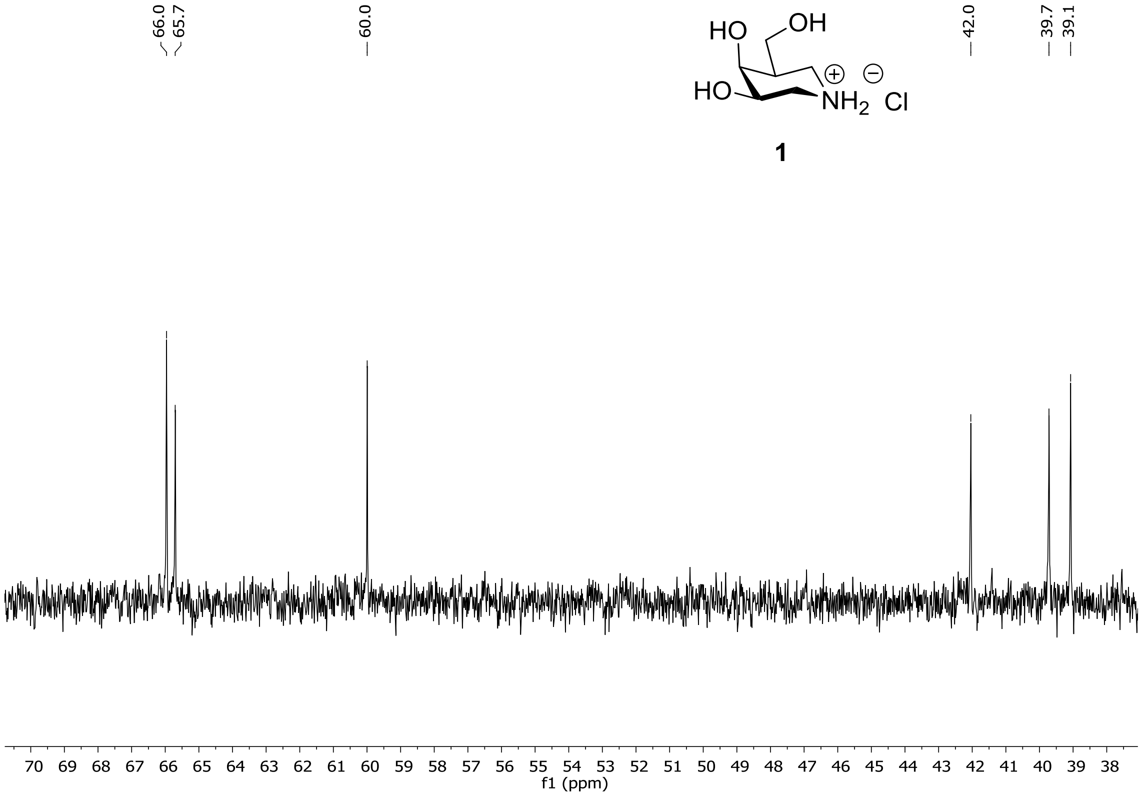

(3*R*,4*S*,5*R*)-1-(*tert*-butoxycarbonyl)- 5-(benzyloxymethyl)-3,4-di-*O*-benzyl-piperidin-2-one (18):

^{1}H-NMR (400 MHz, $CDCl_3$)

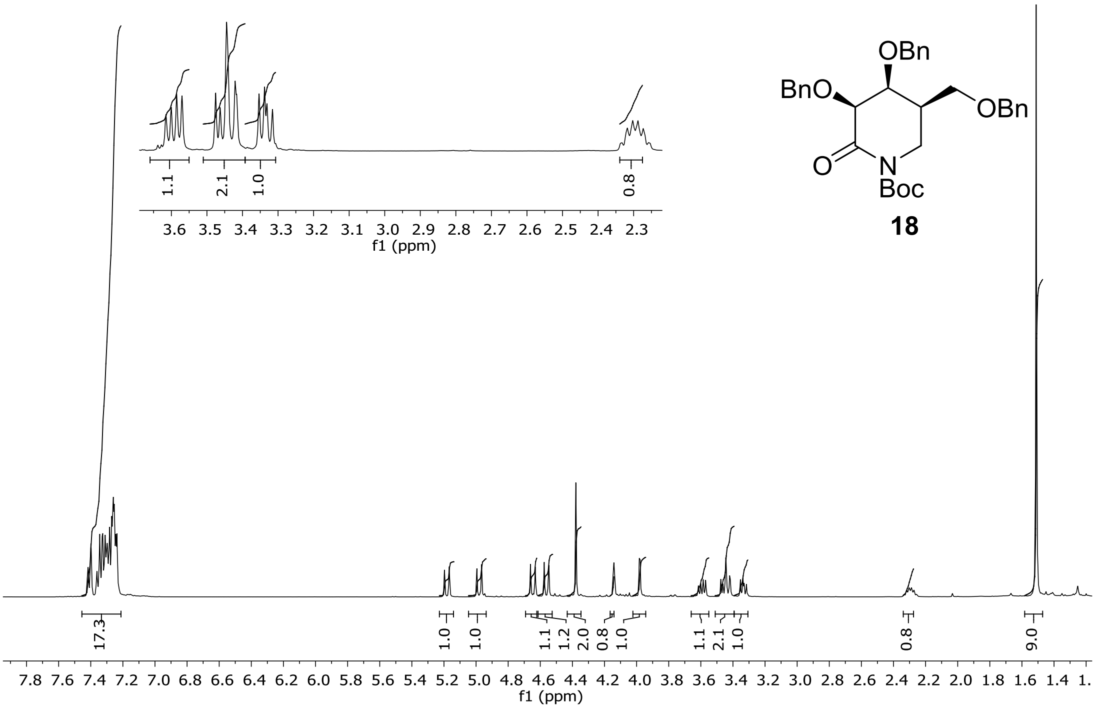

^{13}C-NMR (100 MHz, $CDCl_3$)

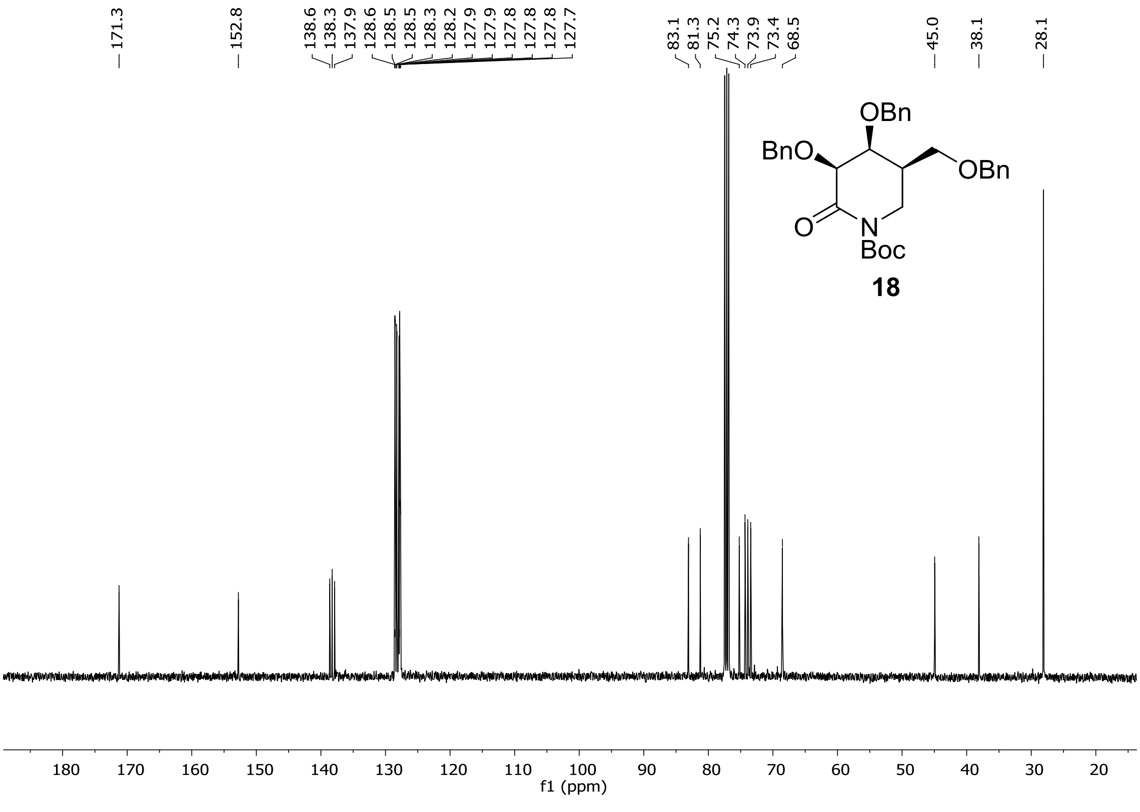

Isogalactofagomine lactam (**3**):

^{1}H-NMR (400 MHz, D_2O)

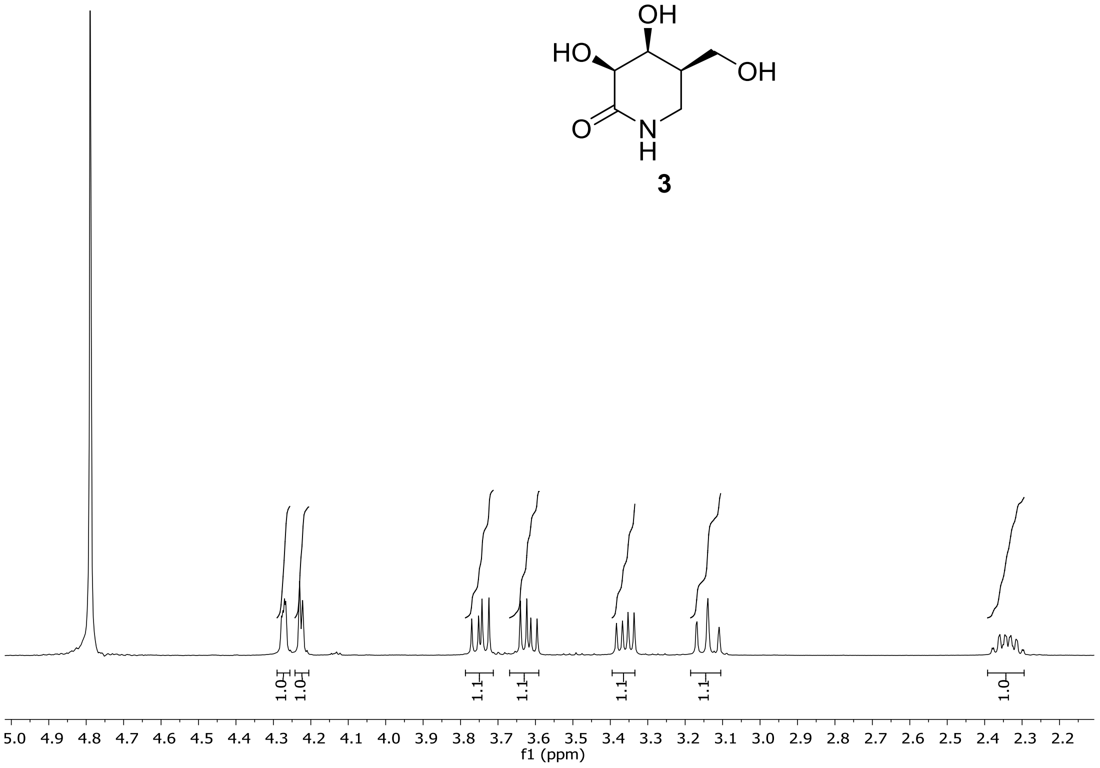

^{13}C-NMR (100 MHz, D_2O)

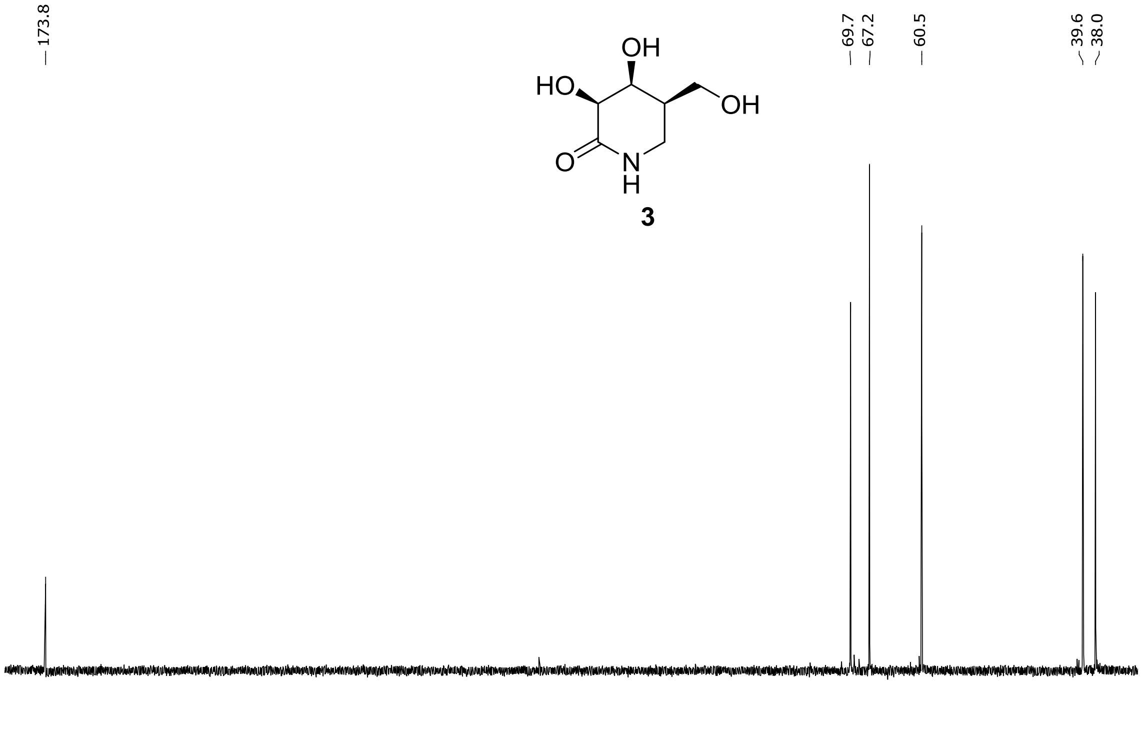

2,3,5-tri-*O*-benzyl-1,4-di-*O*-mesyl-D-ribitol (19):

^{1}H-NMR (400 MHz, $CDCl_3$)

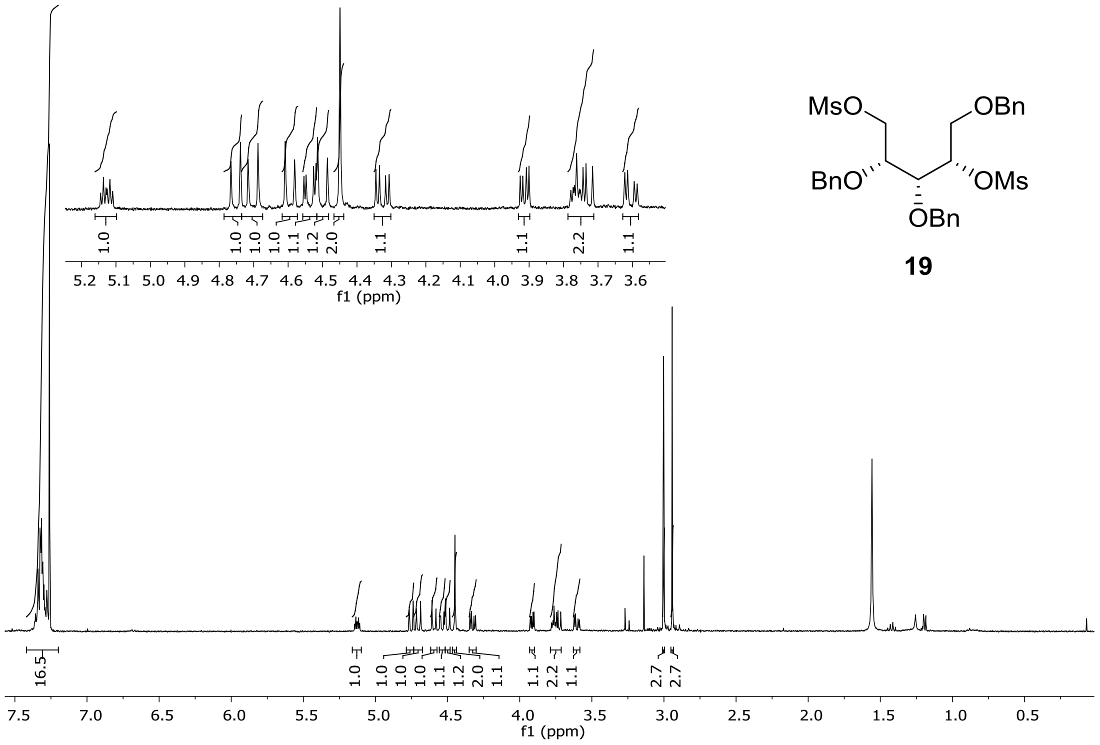

19

^{13}C-NMR (100 MHz, $CDCl_3$)

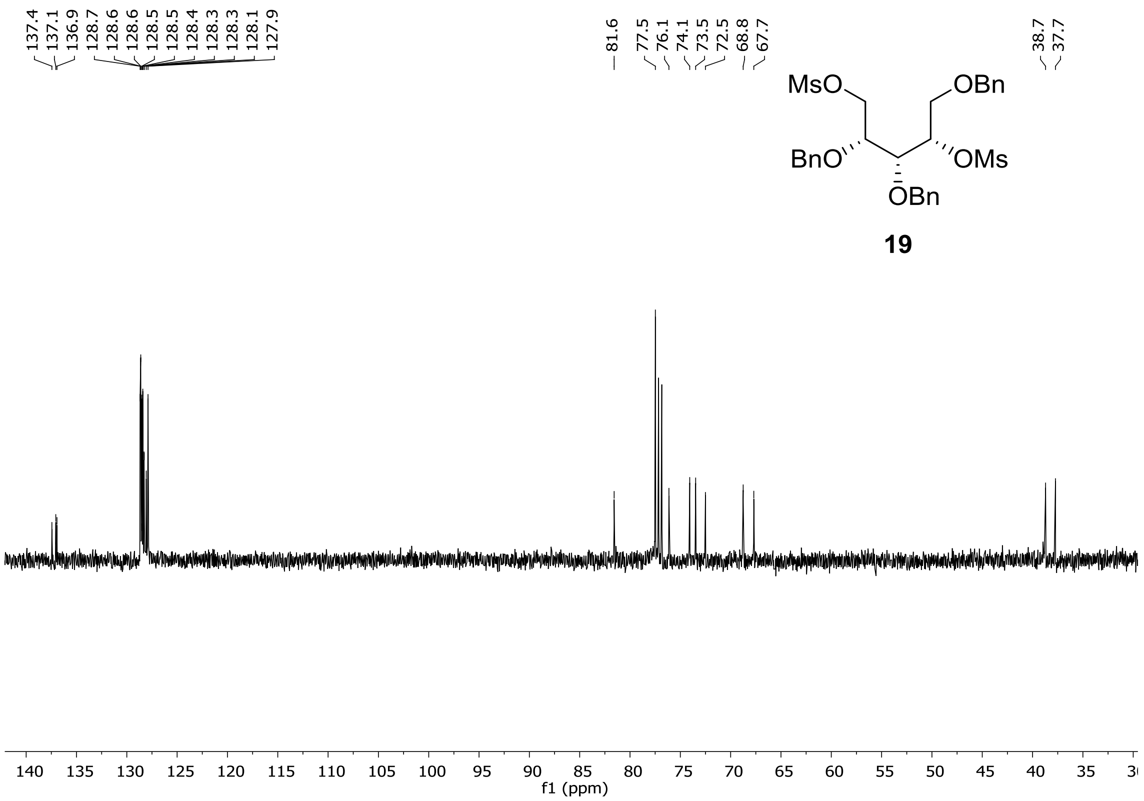

19

(2*R*,3*S*,4*R*)-1-*N*-benzyl-3,4-di-*O*-benzyl-2-((benzyloxy)methyl)pyrrolidine (20):

^{1}H-NMR (400 MHz, $CDCl_3$)

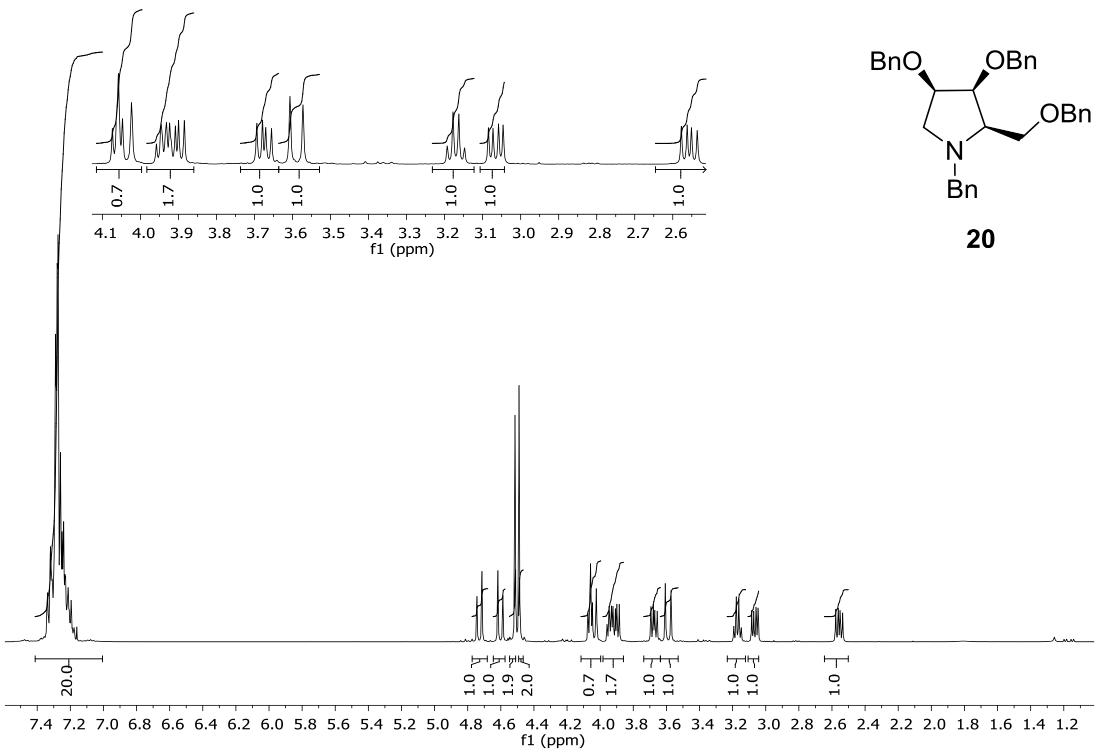

20

^{13}C-NMR (100 MHz, $CDCl_3$)

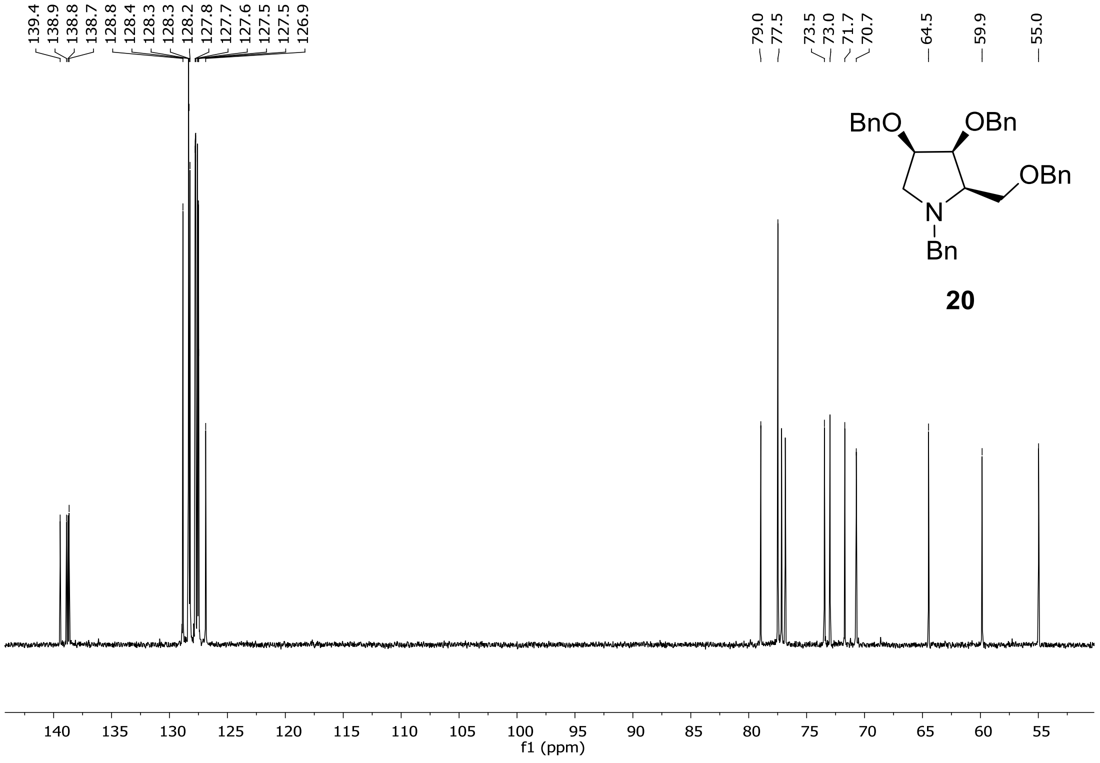

20

1,4-dideoxy-1,4-imino-D-lyxitol (4):

^{1}H-NMR (400 MHz, D_2O)

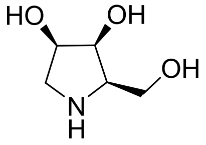

4

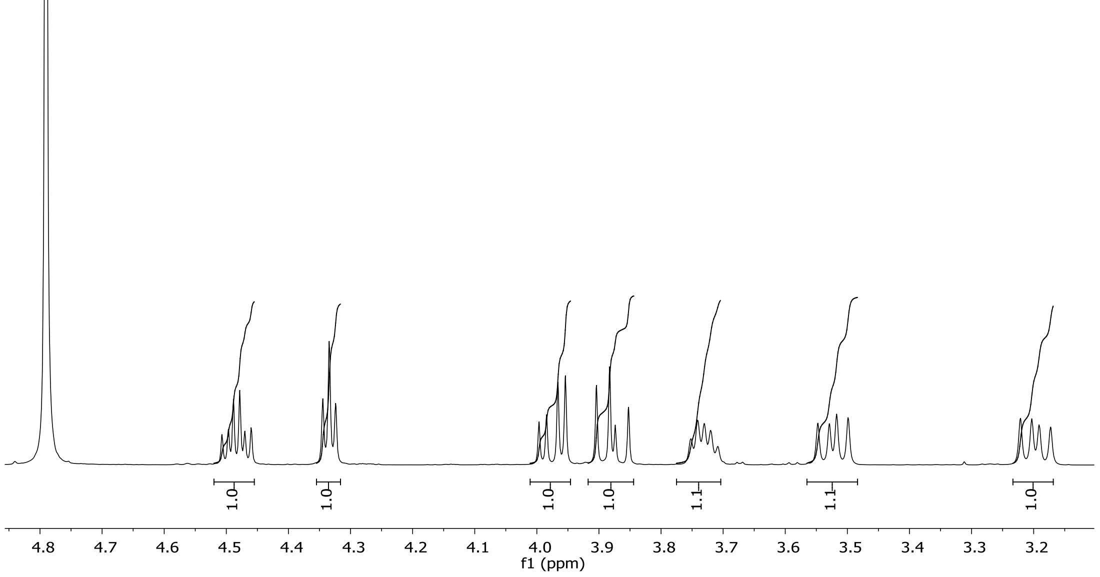

^{13}C-NMR (100 MHz, D_2O)

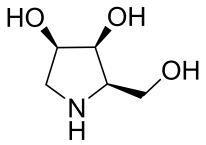

4

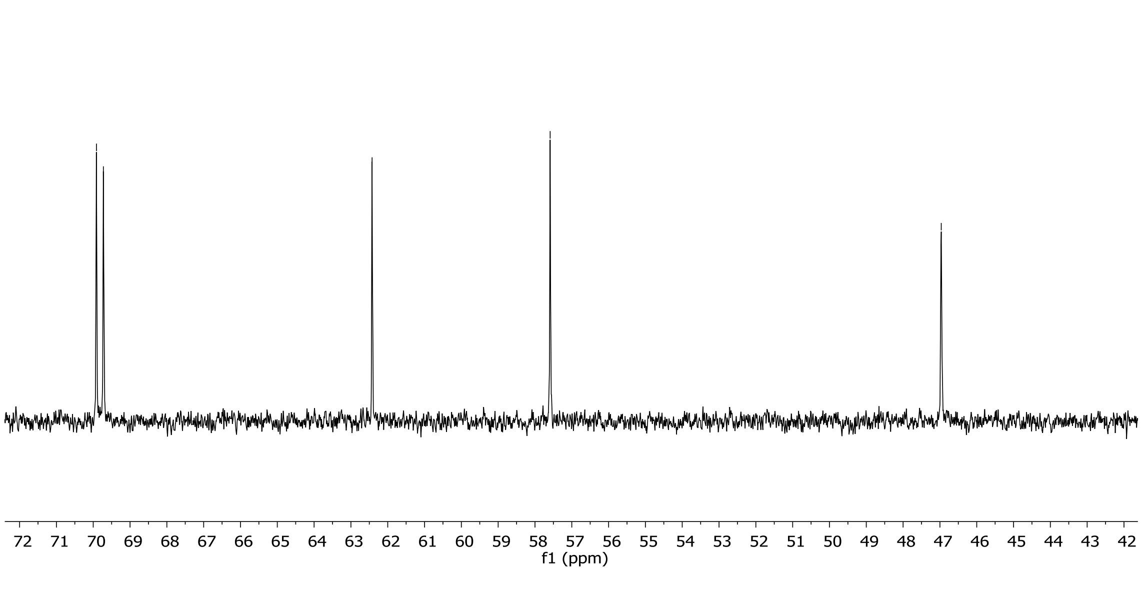

2,3,5-tri-*O*-benzyl-L-ribono-1,4-lactone (21):

^{1}H-NMR (400 MHz, $CDCl_3$)

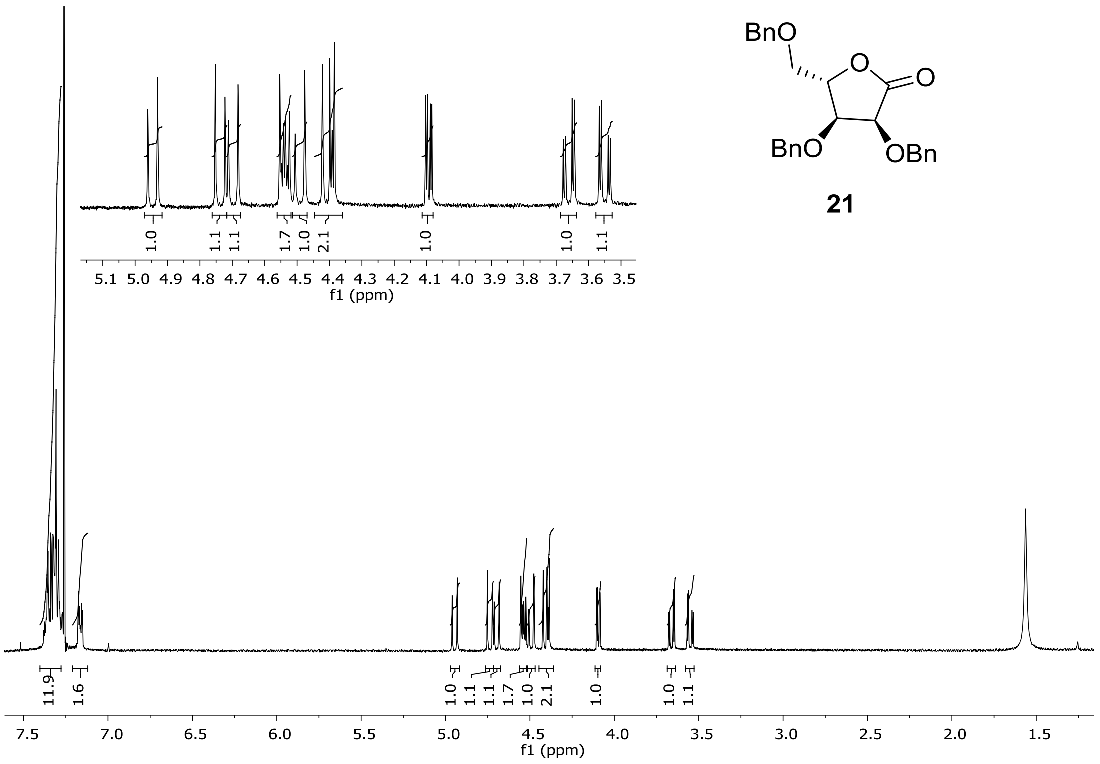

21

^{13}C-NMR (100 MHz, $CDCl_3$)

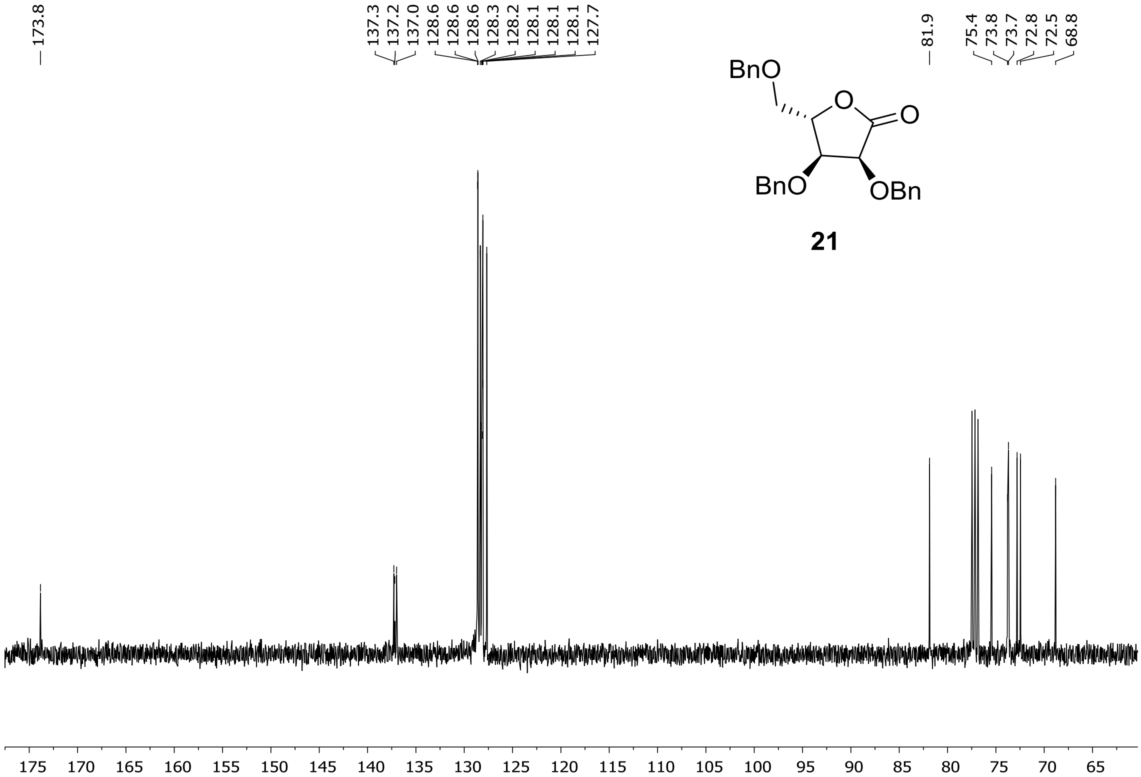

21

2,3,5-tri-*O*-benzyl-L-ribofuranose (22):

^{1}H-NMR (400 MHz, $CDCl_3$)

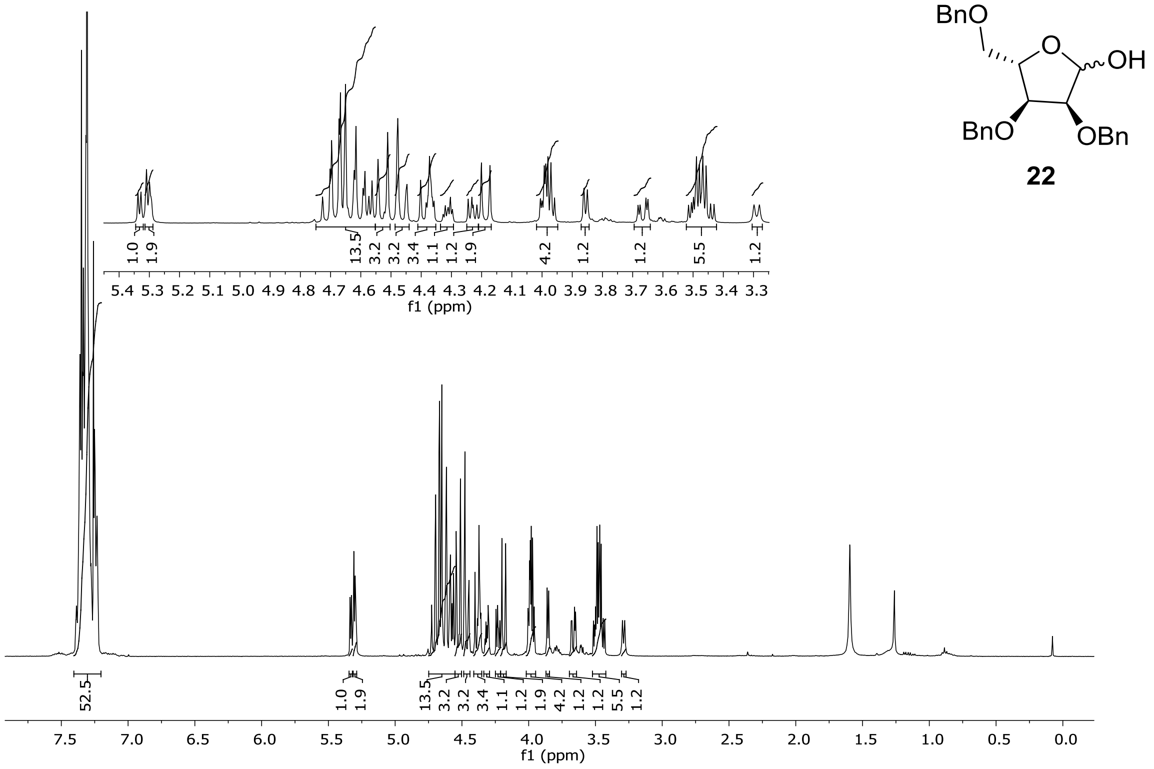

22

^{13}C-NMR (100 MHz, $CDCl_3$)

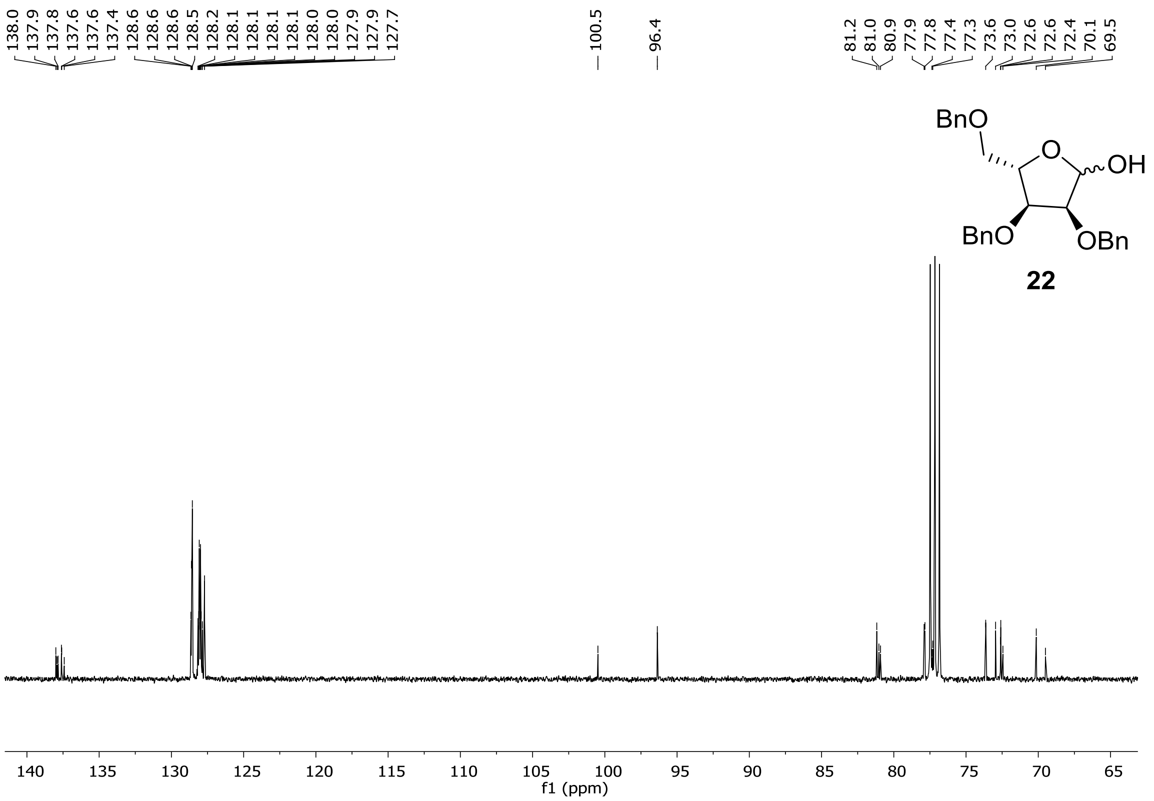

22

1-(2-*tert*-butyloxycarbonyl)hydrazino-1-deoxy-2,3,5-tri-*O*-benzyl-L-ribitol (23):

^{1}H-NMR (400 MHz, $CDCl_3$)

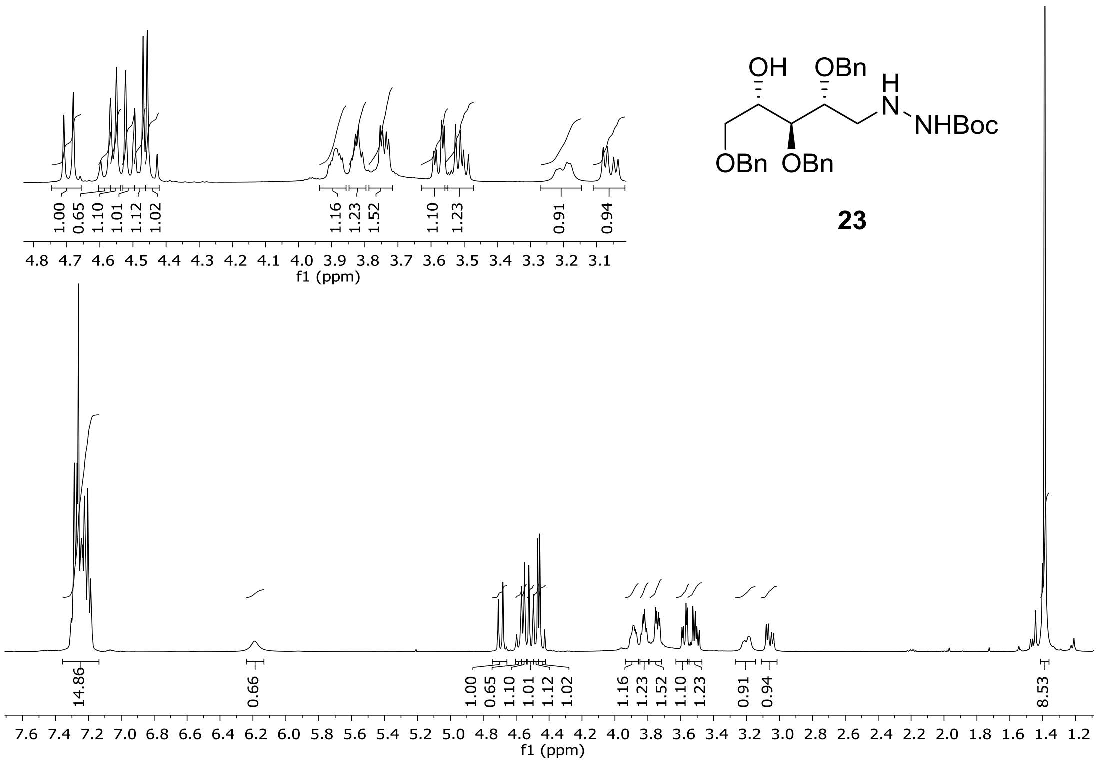

^{13}C-NMR (100 MHz, $CDCl_3$)

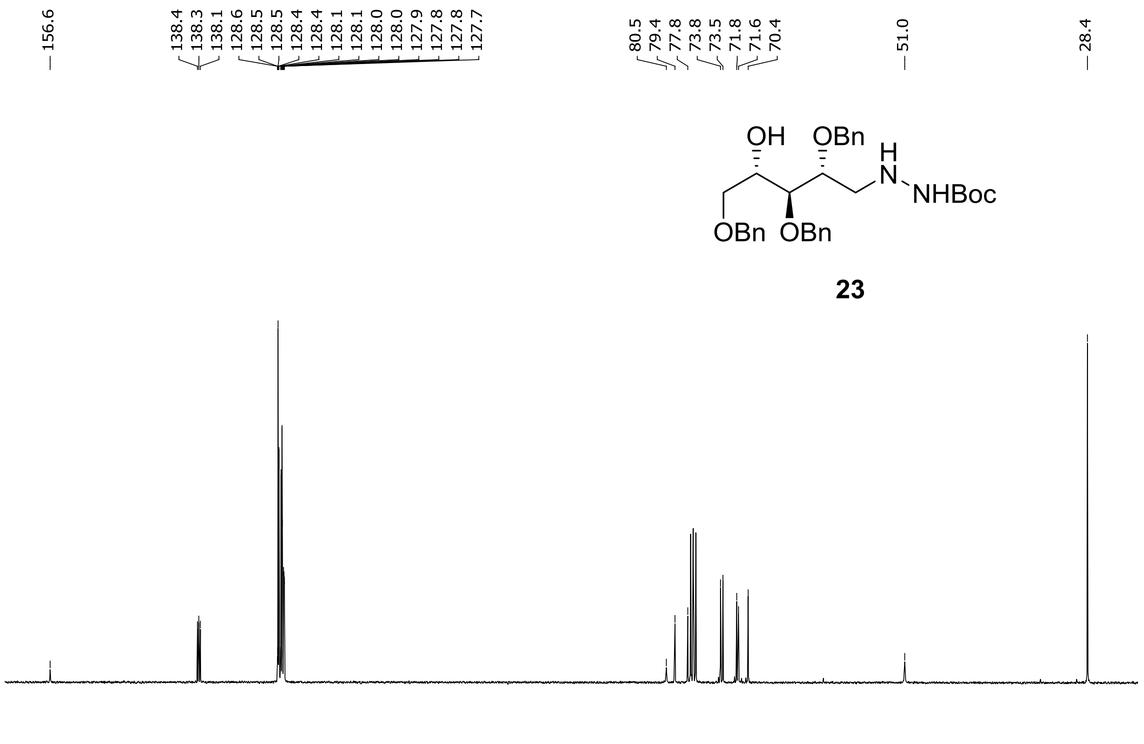

1-(1-Acetyl-2-*tert*-butyloxycarbonyl)hydrazino-1-deoxy-2,3,5-tri-*O*-benzyl-L-ribitol (28)

^{1}H-NMR (400 MHz, $CDCl_3$)

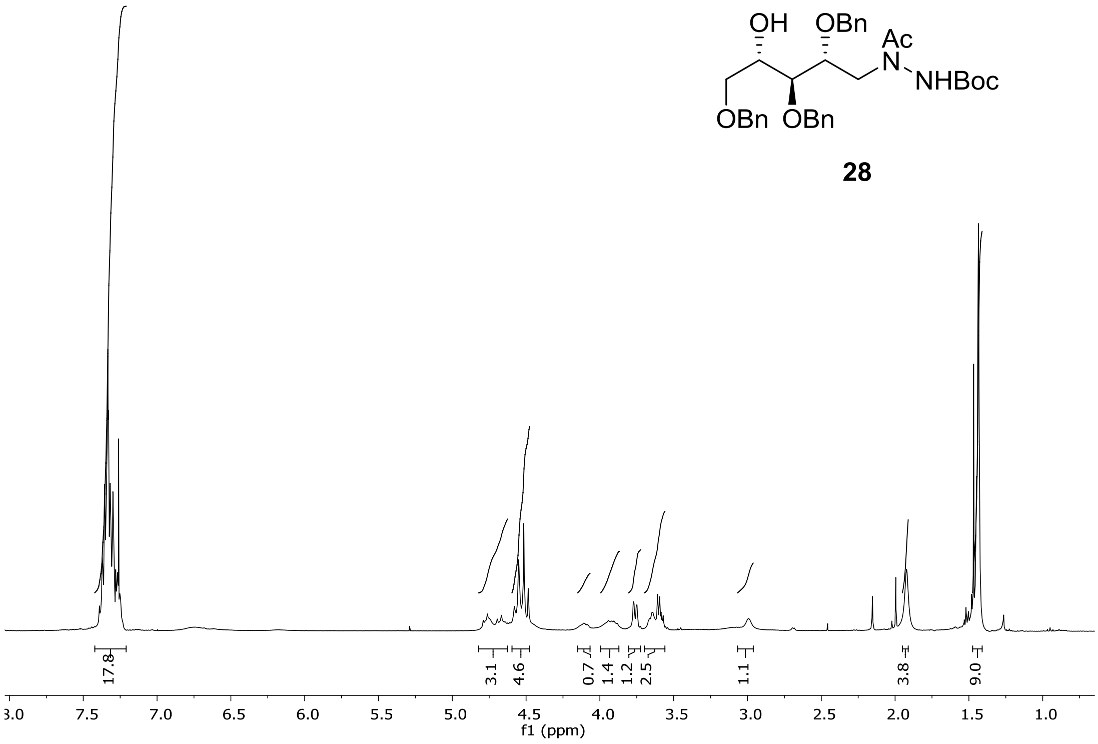

^{13}C-NMR (100 MHz, $CDCl_3$)

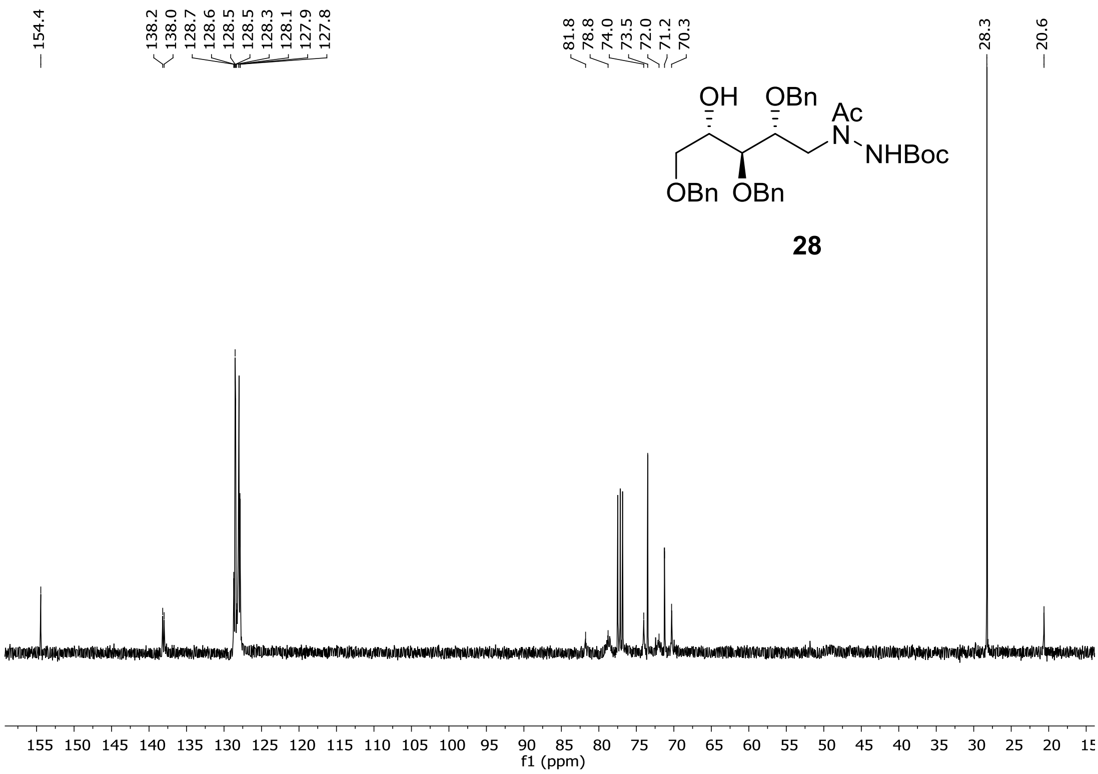

1-(1-Acetyl-2-*tert*-butyloxycarbonyl)hydrazino-1-deoxy-2,3,5-tri-*O*-benzyl-4-methanesulfonyl-L-ribitol (24):

^{1}H-NMR (400 MHz, $CDCl_3$)

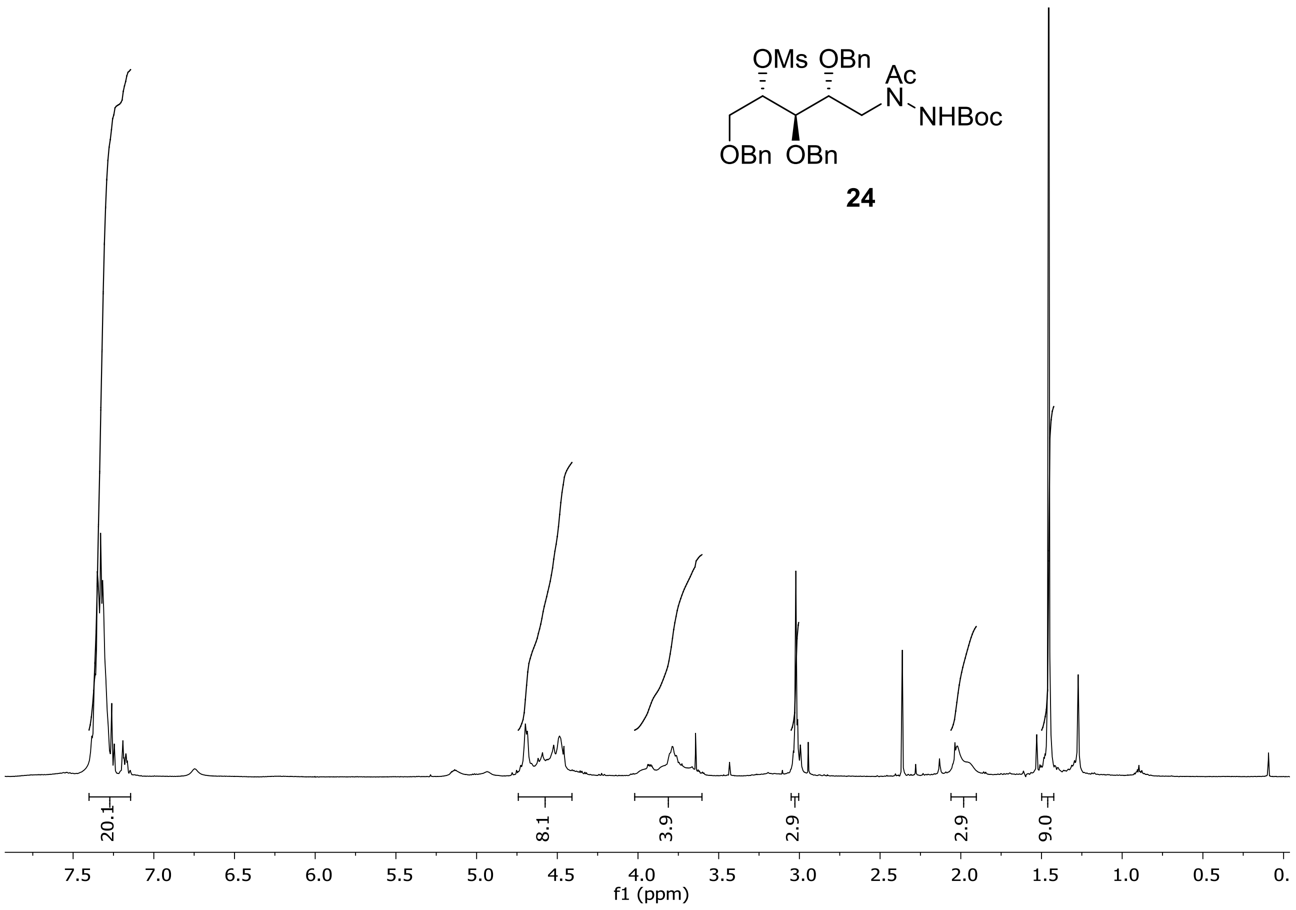

1-(1-Acetyl)hydrazino-1-deoxy-2,3,5-tri-*O*-benzyl-4-methanesulfonyl-L-ribitol (29):

^{1}H-NMR (400 MHz, $CDCl_3$)

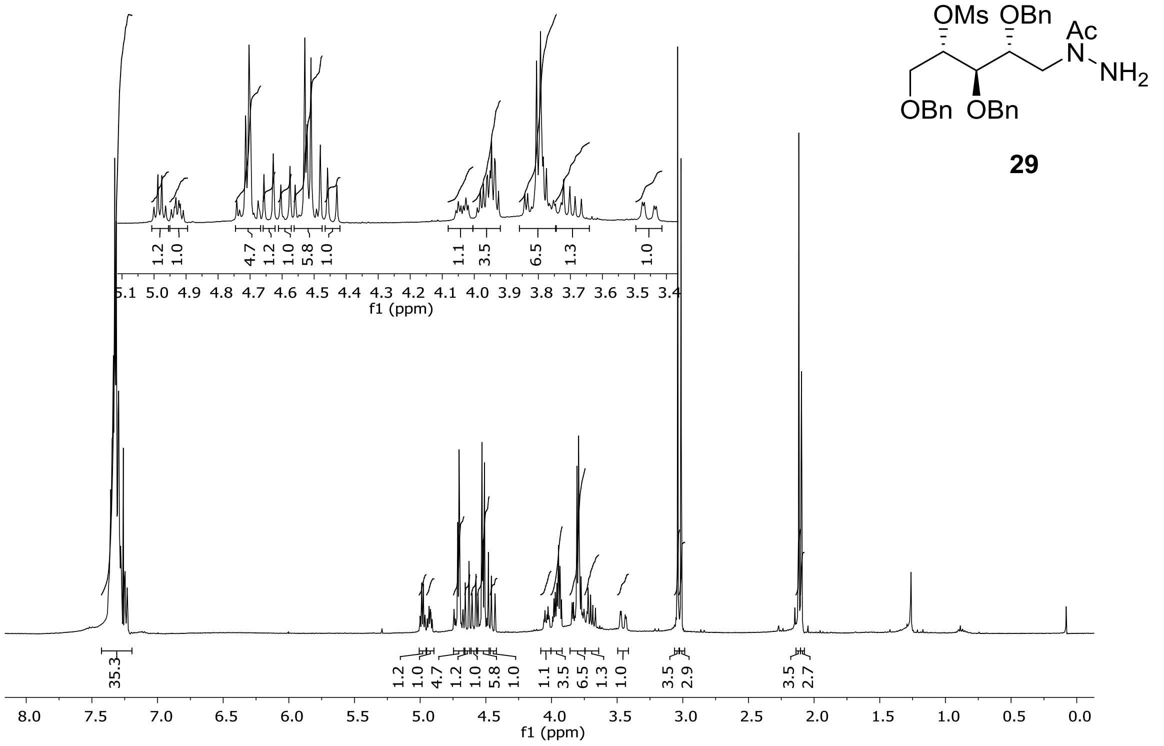

^{13}C-NMR (100 MHz, $CDCl_3$)

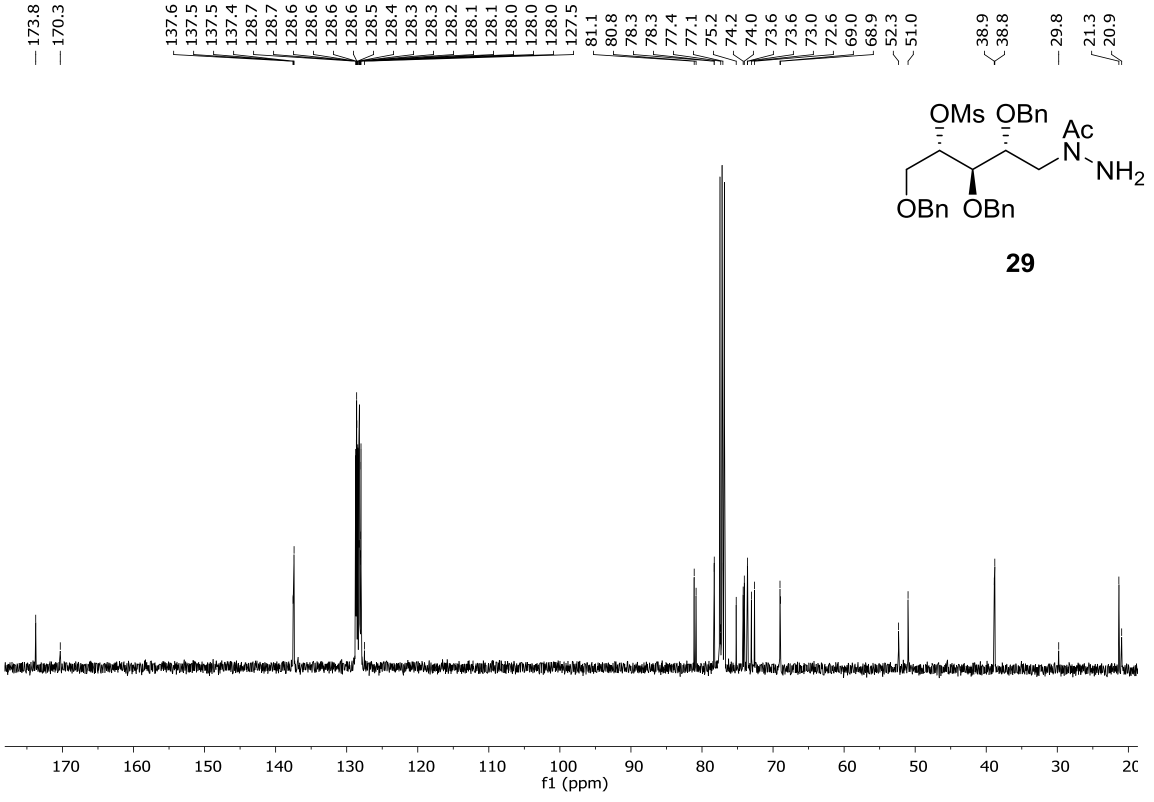

(3*R*,4*S*,5*R*)-1-*N*-acetyl-3-benzyloxymethyl-4,5-di-*O*-benzyl-4,5-dihydroxyhexahydropyrizadine (25):

^{1}H-NMR (400 MHz, $CDCl_3$)

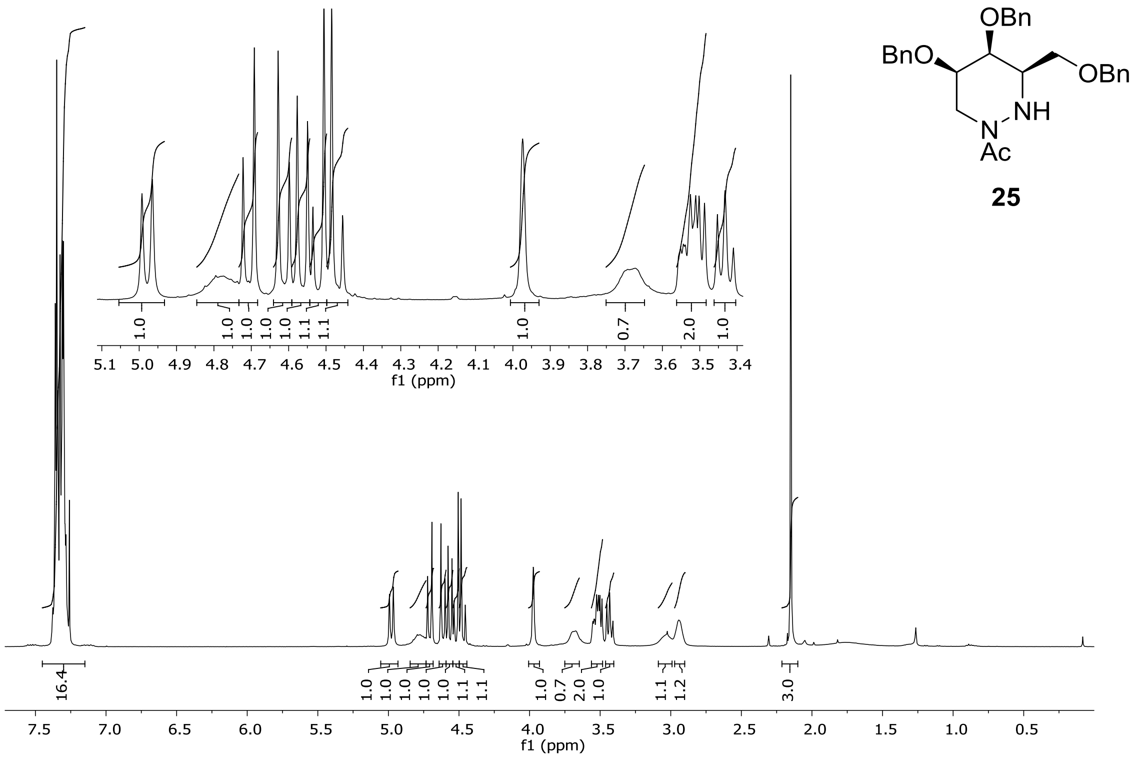

^{13}C-NMR (100 MHz, $CDCl_3$)

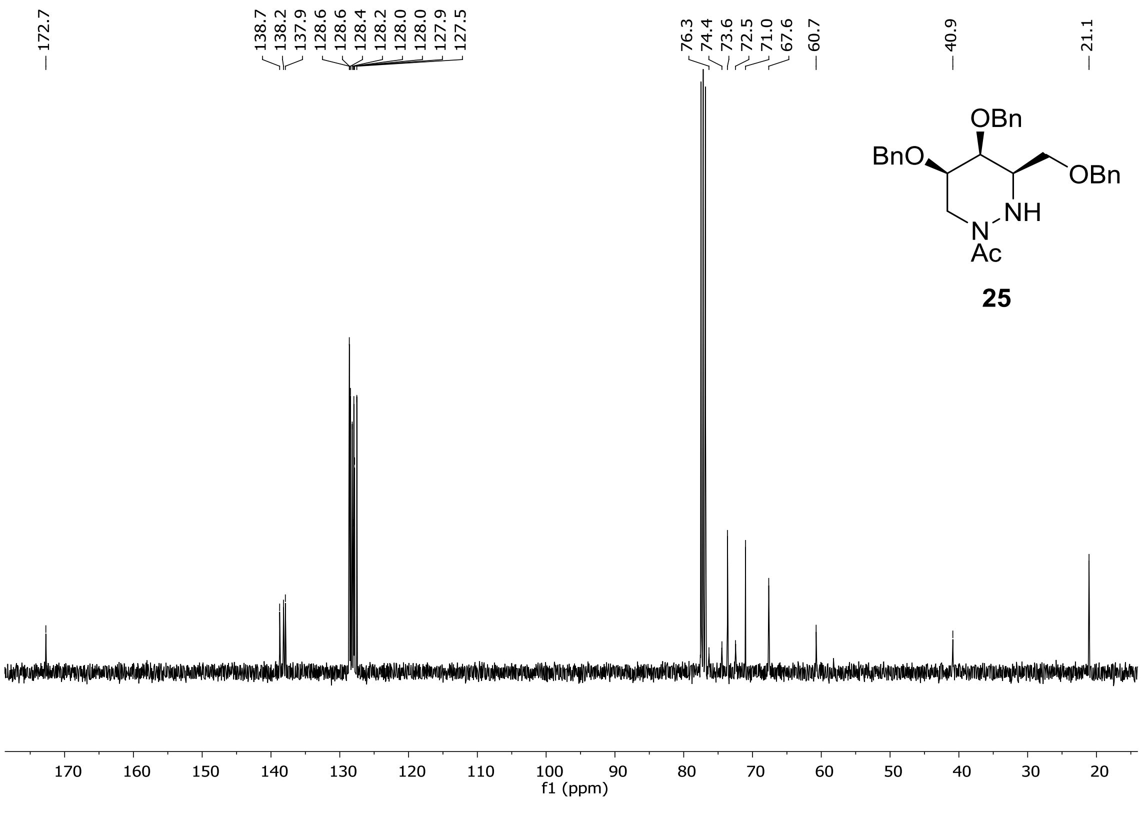

Azagalactofagomine (2):

^{1}H-NMR (400 MHz, D_2O)

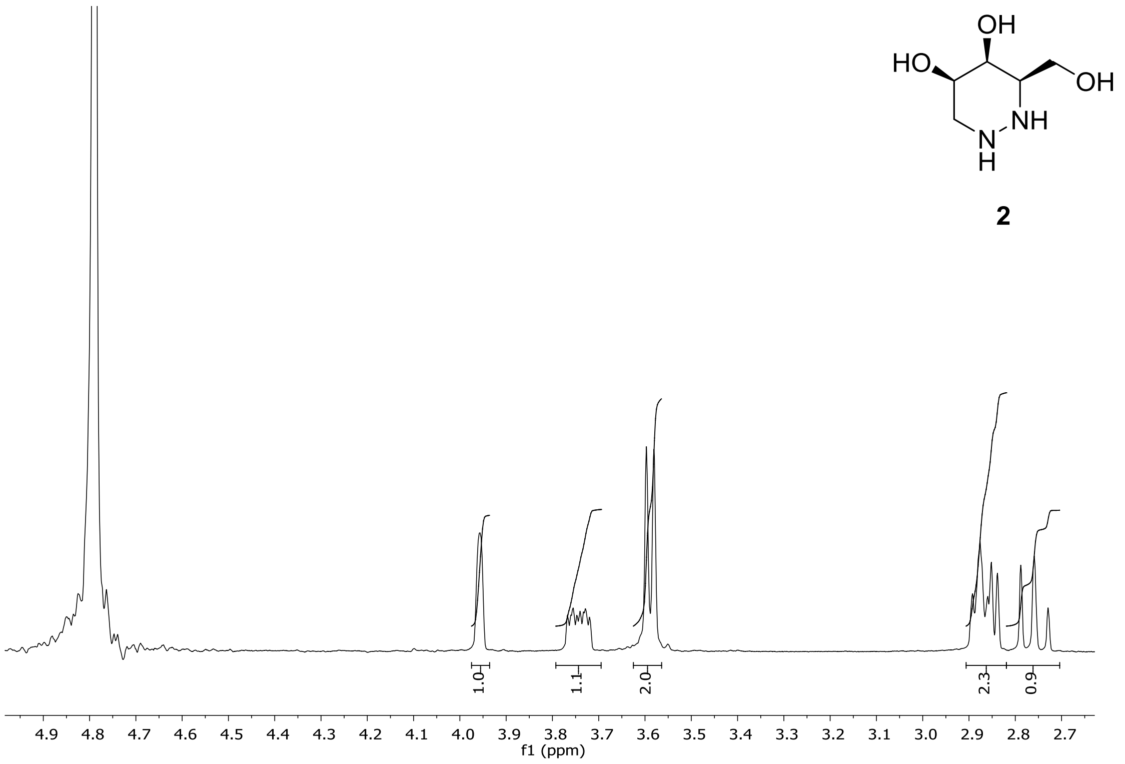

^{13}C-NMR (100 MHz, D_2O)

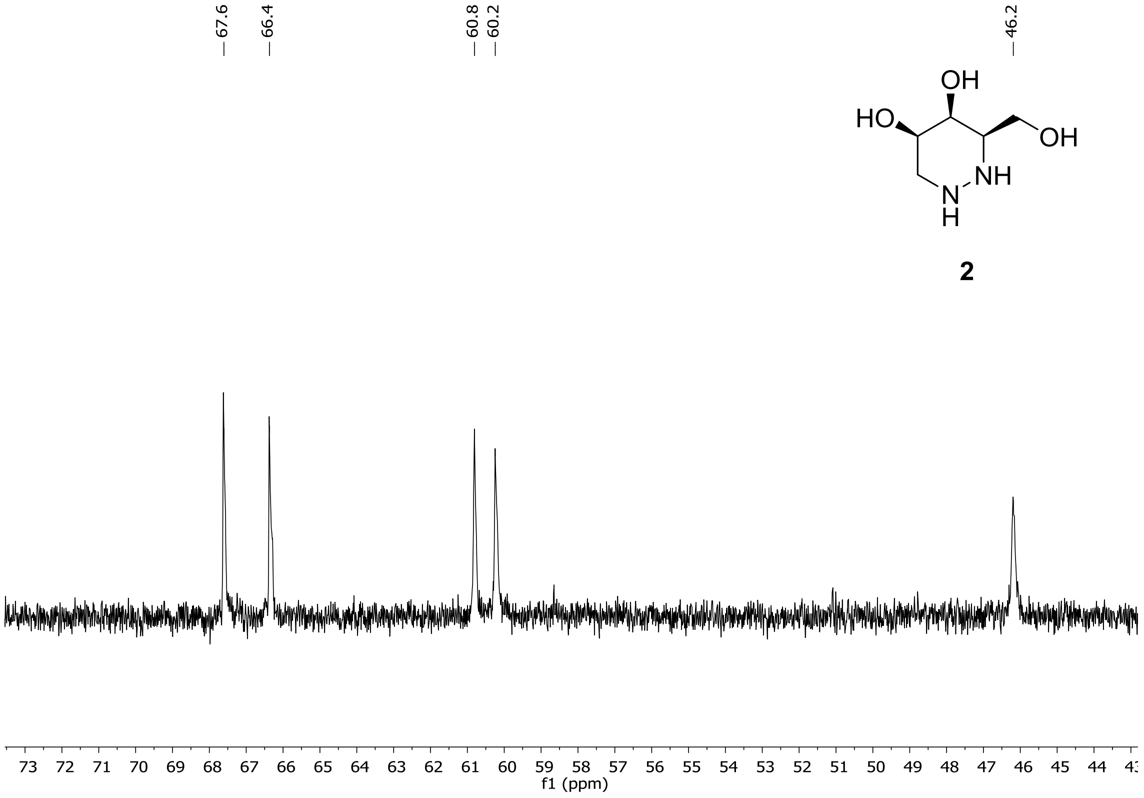

Small-molecule inhibition kinetics

Details of small molecule concentrations used for K_i determination and shown in Figure 3 of the main article are as follows:

(A) DGJ concentrations of 5000 µM (×), 1000 µM (○), 500 µM (◇), 100 µM (▽), 50 µM (△), 10 µM (□) and no inhibitor (●).

(B) IGF concentrations of 10.0 µM (×), 5.0 µM (○), 1.0 µM (◇), 0.5 µM (▽), 0.1 µM (△) and no inhibitor (●).

(C) DIL concentrations of 200 µM (×), 100 µM (○), 50 µM (◇), 25 µM (▽), 10 µM (□) and no inhibitor (●).

(D) AGF concentrations of 1000 µM (×), 750 µM (○), 500 µM (◇), 100 µM (□) and no inhibitor (●).

(E) IGL concentrations 200 µM (×), 100 µM (○), 50 µM (◇), 25 µM (▽), 10 µM (△) and no inhibitor (●).

(F) DGN concentrations of 5000 µM (×), 2500 µM (○), 1250 µM (◇), 625 µM (▽), 312 µM (△) and no inhibitor (●).

Supporting Table S1:

Data collection and refinement statistics

	DGJ	IGF	AGF	IGL	DIL	DGN
**Data collection**						
Space group	*R* 3 2	*R* 3 2	*R* 3 2	*R* 3 2	*R* 3 2	*R* 3 2
Cell dimensions						
a, *b*, *c* (Å)	248.6, 248.6, 77.5	247.2, 247.2, 77.3	249.8, 249.8, 77.8	249.9, 249.9, 77.9	248.8, 248.8, 77.9	248.5, 248.5, 77.7
Resolution (Å)	62.15–2.40 (2.46–2.40)	47.08–2.16 (2.23–2.16)	62.46–2.40 (2.46–2.40)	63.21–2.20 (2.26–2.20)	73.22–2.40 (2.46–2.40)	73.04–2.40 (2.46–2.40)
R_{merge}	0.136 (1.104)	0.165 (1.387)	0.135 (1.496)	0.092 (1.104)	0.115 (1.180)	0.143 (0.645)
$\langle I / \sigma I \rangle$	10.7 (1.9)	12.3 (2.1)	13.2 (1.7)	15.7 (2.0)	15.1 (2.1)	7.0 (1.9)
$CC_{1/2}$	0.996 (0.712)	0.998 (0.593)	0.998 (0.614)	0.999 (0.698)	0.998 (0.671)	0.982 (0.639)
Completeness (%)	99.6 (99.8)	99.7 (100.0)	99.9 (99.8)	99.9 (99.9)	99.7 (99.3)	97.9 (98.9)
Multiplicity	8.6 (8.9)	15.1 (14.6)	10.5 (10.6)	8.6 (8.5)	10.4 (10.4)	5.1 (5.3)
**Refinement**						
Resolution (Å)	62.15–2.40 (2.47–2.40)	47.08–2.16 (2.20–2.16)	62.46–2.40 (2.46–2.40)	63.21–2.20 (2.25–2.20)	73.22–2.40 (2.47–2.40)	73.04–2.40 (2.47–2.40)
No. reflections	35589 (2744)	47992 (2661)	36134 (2736)	46932 (2742)	35733 (2727)	34998 (2727)
R_{work} / R_{free}	0.171/ 0.209	0.190/0.215	0.159/0.201	0.165/0.198	0.153/0.194	0.178/0.210
Ramachandran favoured (%)	96.1	95.2	96.7	96.6	96.6	95.8
Ramachandran outliers (%)	0.5	0.2	0.3	0.3	0.3	0.6
No. atoms						
Protein	5098	5234	5132	5123	5140	5260
Carbohydrate	98	98	98	98	98	98
Ligand	11	10	10	11	9	12
Ca^{2+} ion	1	1	1	1	1	1
Water	212	214	197	196	181	223
B-factors						
Protein	51.6	47.7	47.7	47.2	46.6	48.2
Carbohydrate	60.1	59.8	58.1	62.8	56.6	65.6
Ligand	35.8	26.8	30.2	40.9	33.4	36.3
Ca^{2+} ion	51.3	42.7	46.3	57.2	53.2	61.5
Water	48.2	40.3	41.7	42.7	44.0	43.3
r.m.s. deviations						
Bond lengths (Å)	0.003	0.004	0.003	0.005	0.007	0.007
Bond angles (°)	0.802	0.775	0.775	0.932	0.977	1.266

Values in parentheses are for highest-resolution shell. One crystal was used per structure.

Figure S1-S5

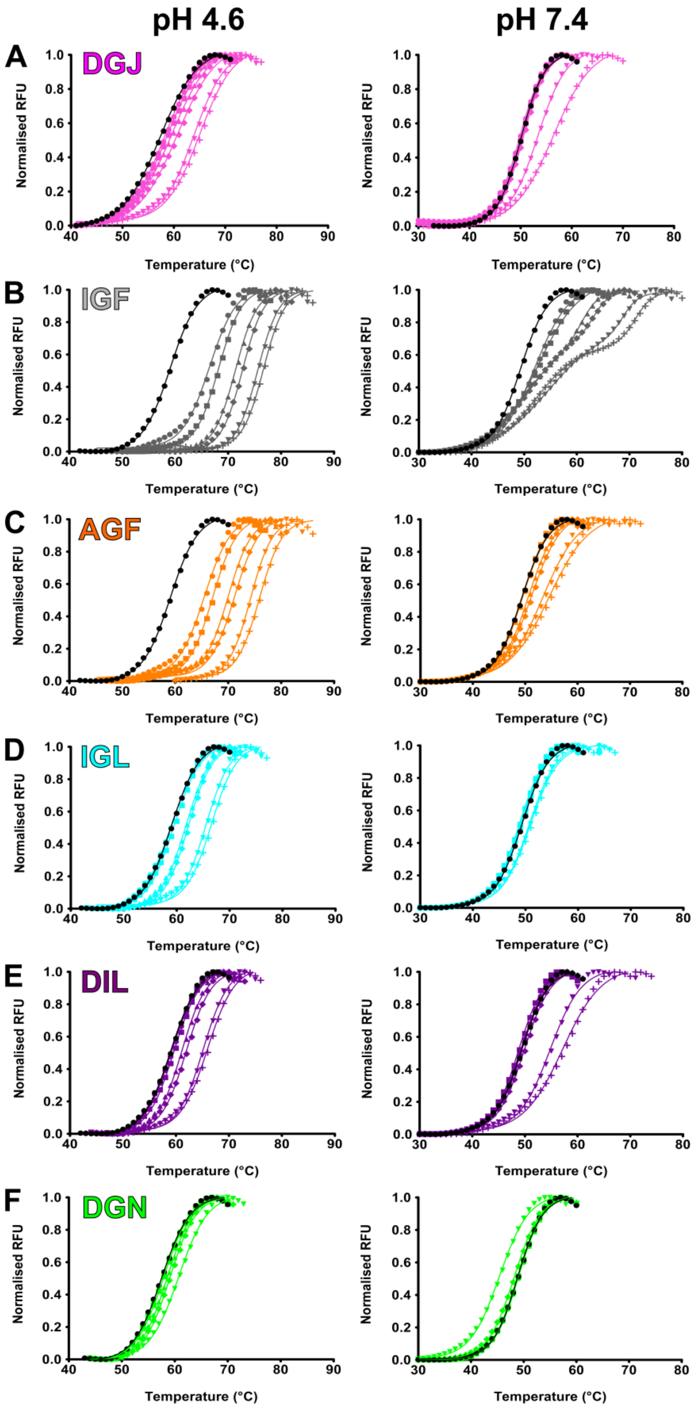

Figure S1. Thermal stabilization of GALC by galacto-configured azasugars is dose-dependent. DSF experiments were performed in sodium acetate, pH 4.6 and PBS, pH 7.4 with 5.0 µg mGALC and compounds (A) DGJ, (B) IGF, (C) AGF, (D) IGL (E) DIL and (F) DGN. Overlaid melt curves of mGALC alone (black) and with each compound (colored) at concentrations of 50 µM (●), 100 µM (■), 500 µM (▲), 1.0 mM (◆), 5.0 mM (▼) and 10 mM (✚).

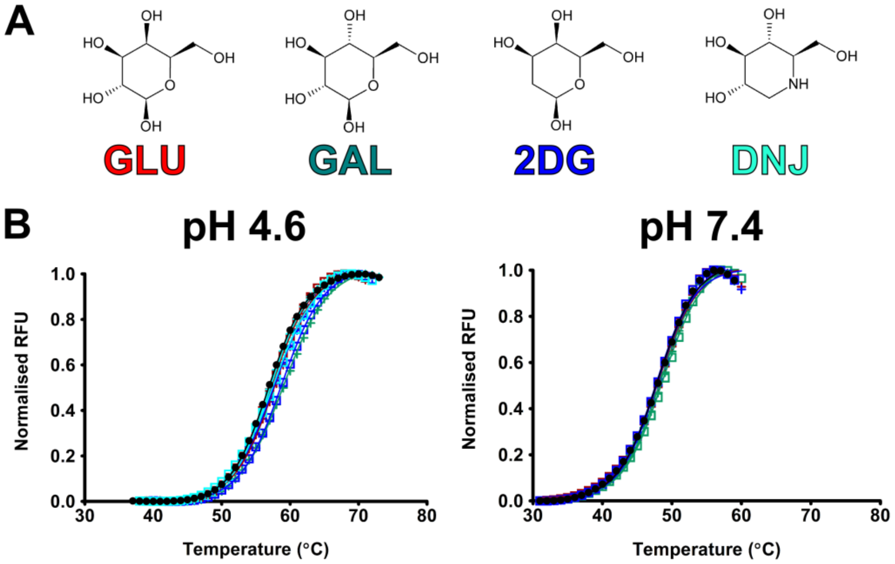

Figure S2. Negative controls for DSF assays. (A) Skeletal formulae of GLU, GAL, 2DG and DNJ, color coded as indicated. Experiments were performed in sodium acetate, pH 4.6 and PBS, pH 7.4 with 5.0 µg mGALC. (B) Overlaid melt curves of mGALC alone (black) and with each compound (colored) at concentrations of 10 mM (+) and 50 mM (☐).

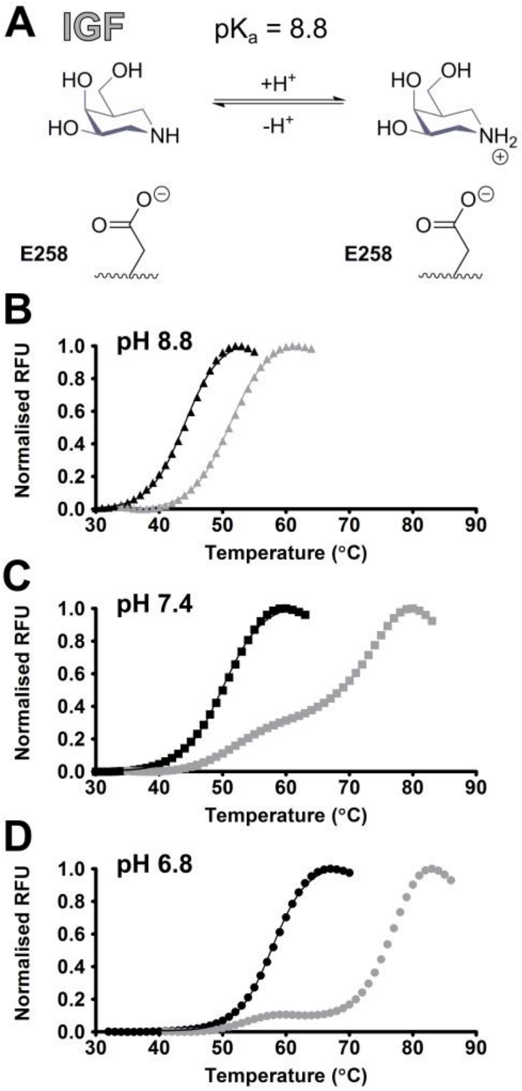

Figure S3. Biphasic IGF-mediated stabilization of GALC is pH-dependent. (A) Schematic of IGF-GALC interaction in two possible binding modes, dependent on protonation state. (B-D) Melt curves of GALC alone (black) and with 10 mM IGF (grey) in phosphate buffer at pH 8.8 (B), 7.4 (C) and 6.8 (D).

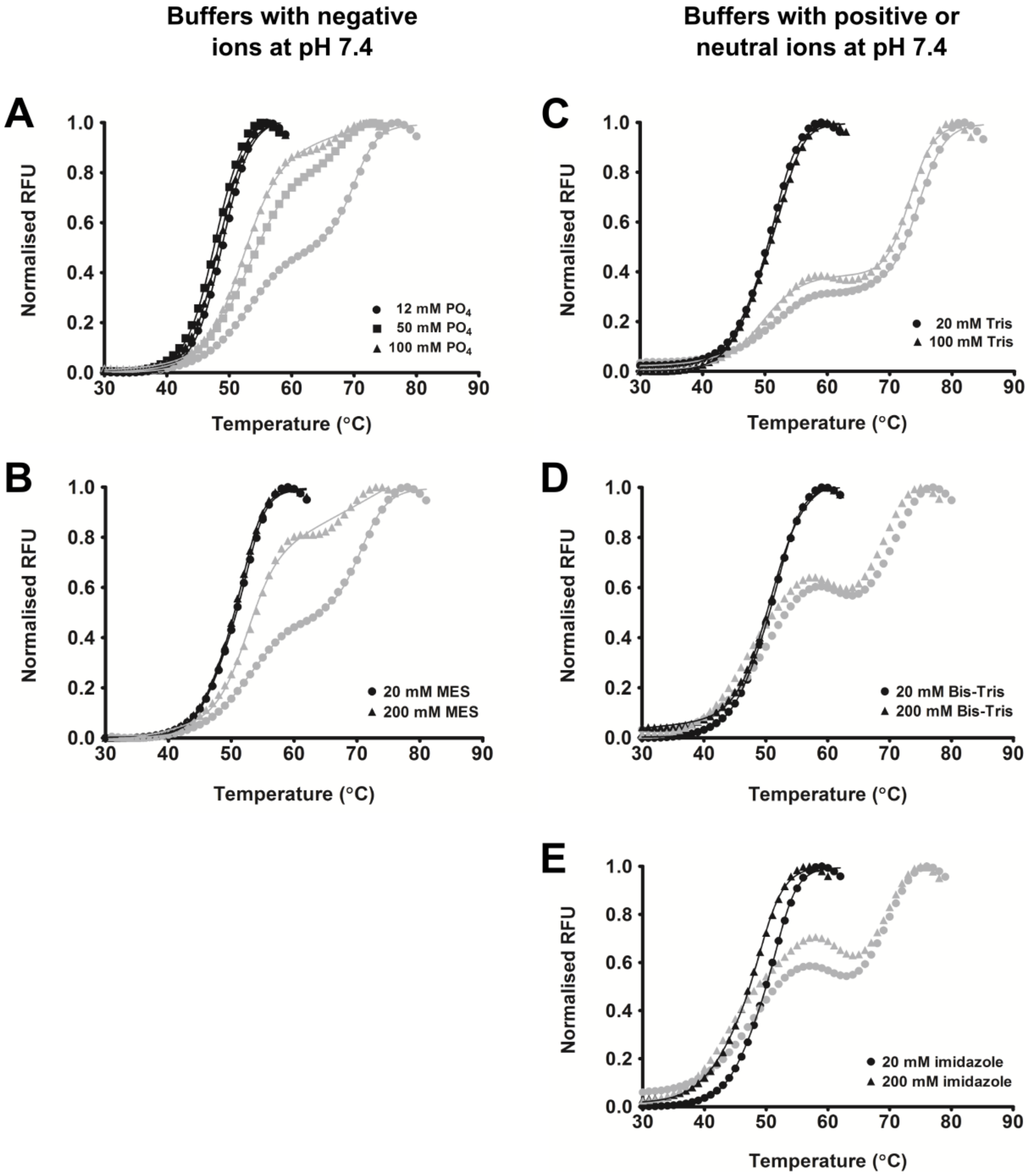

Figure S4. IGF-mediated stabilization of GALC is buffer dependent. Experiments were conducted with mGALC alone (black) and with 10 mM IGF (grey, as above) in buffers with negative ions at pH 7.4 (A) phosphate, (B) MES; and buffers with positive or neutral ions at pH 7.4 (C) Tris, (D) Bis-Tris, (E) Imidazole. Several buffer concentrations were used, as indicated in the legend.

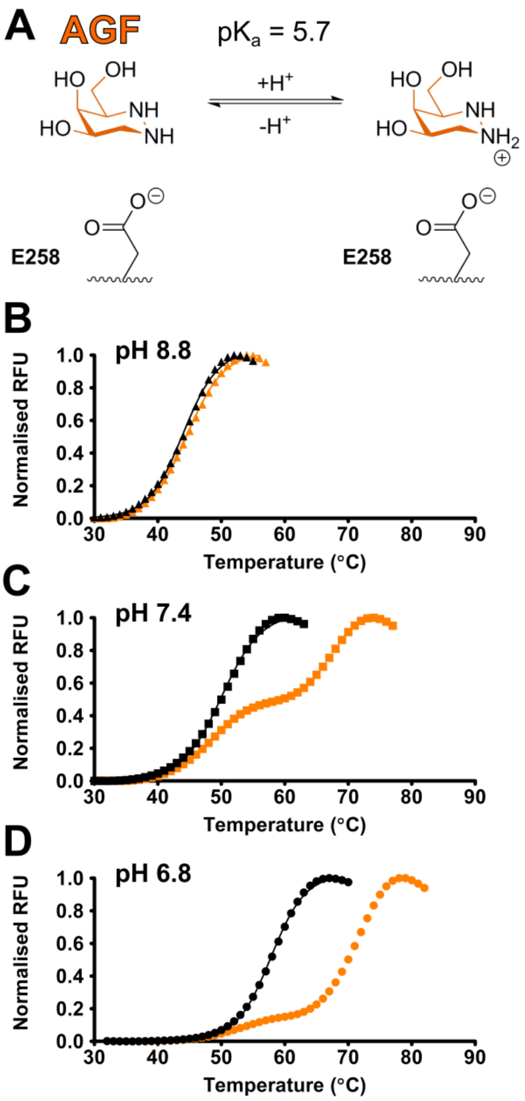

Figure S5. Biphasic AGF-mediated stabilization of GALC is pH-dependent. (A) Schematic of AGF-GALC interaction in two possible binding modes, dependent on protonation state. (B-D) Melt curves of GALC alone (black) and with 10 mM AGF (orange) in Tris buffer at pH 8.8 (B), 7.4 (C) and 6.8 (D). The biphasic melt curves were only observed with AGF when Tris buffer was used.

References

[1] Pedersen, D. S.; Rosenbohm, C. *Synthesis* **2001**, 2431.

[2] Ichikawa, Y.; Igarashi, Y.; Ichikawa, M.; Suhara, Y. *J. Am. Chem. Soc.* **1998**, *120*, 3007

[3] Søhoel, H.; Liang, X.; Bols, M. *J. Chem. Soc. Perkin Trans. 1,* **2001**, 1584.

[4] Dangerfield, E. M.; Gulab, S. A.; Plunkett, C. H.; Timmer, M. S. M.; Stocker, B. L. *Carbohydr. Res.* **2010**, *345*, 1360.

[5] Alessandrini, L.; Casati, S.; Ciuffreda, P.; Ottria, R.; Santaniello, E. *J. Carbohydr. Chem.* **2008**, *27*, 332.

[6] Jensen, H. H.; Bols, M. *J. Chem. Soc. Perkin Trans. 1* **2001**, 905.
